# Supplementary material for: DeepBacs for multi-task bacterial image analysis using open-source deep learning approaches
Source: Commun Biol. 2022 Jul 9;5:688. doi: 10.1038/s42003-022-03634-z (PMC9271087; doi:10.1038/s42003-022-03634-z)
Supplement: Supplementary file 2 — Supplementary Information [file 42003_2022_3634_MOESM2_ESM.pdf]

Supplementary information

DeepBacs for multi-task bacterial image analysis using open-source deep learning approaches

Christoph Spahn[1,2 *], Estibaliz Gómez-de-Mariscal[3], Romain F. Laine[4,5,6], Pedro M. Pereira[7], Lucas von Chamier[5], Mia Conduit[8], Mariana G. Pinho[7], Guillaume Jacquemet[9,10,11], Séamus Holden[8], Mike Heilemann[2 *] and Ricardo Henriques[3,5,6 *]

[1] Department of Natural Products in Organismic Interaction, Max Planck Institute for Terrestrial Microbiology, Marburg, Germany

[2] Institute of Physical and Theoretical Chemistry, Goethe-University Frankfurt, Frankfurt, Germany

[3] Instituto Gulbenkian de Ciência, 2780-156 Oeiras, Portugal

[4] Current address: Micrographia Bio, Translation and Innovation hub 84 Wood lane, W120BZ, London, UK

[5] MRC-Laboratory for Molecular Cell Biology, University College London, London, UK

[6] The Francis Crick Institute, London, UK

[7] Instituto de Tecnologia Química e Biológica António Xavier, Universidade Nova de Lisboa, Oeiras, Portugal.

[8] Centre for Bacterial Cell Biology, Newcastle University Biosciences Institute, Faculty of Medical Sciences, Newcastle upon Tyne NE24AX, United Kingdom

[9] Turku Bioscience Centre, University of Turku and Åbo Akademi University, Turku, Finland

[10] Faculty of Science and Engineering, Cell Biology, Åbo Akademi University, Turku, Finland

[11] Turku Bioimaging, University of Turku and Åbo Akademi University, Finland

Supplementary Notes

Supplementary Note 1 - Prediction quality assessment metrics

Testing the performance of trained DL models is crucial to obtain reliable results. For this purpose, different metrics are commonly used, depending on the data type and DL task. All metrics used in this study are described in detail in the Supplementary Information of the ZeroCostDL4Mic publication [1].

For segmentation tasks, we employed the Intersection-over-Union (IoU), Recall and Precision metrics.

IoU, also known as the Jaccard index, reports on the overlap between ground truth (GT) and predicted masks (P) and is calculated according to equation 1. A value of one thus represents perfect overlap, declining towards 0 with decreasing overlap.

$$IoU\ (GT, P) = \frac{|GT \cap P|}{|GT \cup P|} \qquad \text{(Eq. 1)}$$

While the IoU metric gives access to the performance of semantic segmentation (i.e. classification in the background and foreground pixels), it does not report on the quality of instance segmentation. A dataset might have a high IoU, but individual instances may not be resolvable (see CARE and pix2pix predictions of dense regions in Figure S1). Instead, we use the *Recall* and *Precision* metrics (Eq. 2 and 3, respectively), whose calculation is based on the true positives (TP), false negatives (FN) and false positives (FP) determined using the test dataset.

$$Recall = \frac{TP}{TP + FN} \qquad \text{(Eq. 2)}$$

$$Precision = \frac{TP}{TP + FP} \qquad \text{(Eq. 3)}$$

Recall thus describes the sensitivity of the analysis or prediction, while *Precision* describes the specificity.

In object detection, model performance is assessed using the Average Precision (AP). AP is calculated via so-called Precision-Recall-Curves. These curves are generated by applying different thresholds for object detection, followed by the calculation of Precision and Recall values for each threshold. Lowering the detection threshold leads to the detection of more

instances (Recall) but typically leads to a reduced Precision, as also more false positives are detected. The AP value is finally received by determining the area under the curve. *mean Average Precision* (mAP) represents the mathematical mean of the AP values of all classes present in the test dataset.

The quality of image denoising was quantified using the structural similarity index (SSIM) [2] and peak signal-to-noise ratio (PSNR). SSIM describes the similarity of two images A and B based on mean pixel intensities (μ_A, μ_B), pixel intensity variation (μ_A^2, μ_B^2) and pixel intensity covariance (σ_{AB}) (Equ. 4).

$$SSIM\,(A,B) \;=\; \frac{(2\mu_A\mu_B \;+\; C_1)(2\sigma_{AB} \;+\; C_2)}{(\mu_A^2 \;+\; \mu_B^2 \;+\; C_1)\,(\mu_A^2 \;+\; \mu_B^2 \;+\; C_2)} \qquad \text{(Eq. 4)}$$

Constants C_1 and C_2 were introduced for stability and are defined according to Equ. 5 and 6.

$$C_1 \;= (K_1 L)^2 \qquad \text{(Eq. 5)}$$

$$C_2 \;= (K_2 L)^2 \qquad \text{(Eq. 6)}$$

L represents the dynamic range of the images (maximal number of grey values), and K_1 and K_2 are constants that were set to 0.01 and 0.03 as suggested in the original publication (Wang *et al.*, 2004).

PSNR was originally introduced to compare the preservation of image quality in compressed images. However, PSNR can also be used to quantify denoising performance when ground-truth data is available. Its calculation is based on the mean squared error between two images, A (high SNR) and B (low SNR) (MSE, Eq. 7), and follows Eq. 8.

$$MSE \;= \frac{1}{n}\sum_{i,j}^{n}(A_{ij} \;-\; B_{i,j})^2 \qquad \text{(Eq. 7)}$$

with *n* representing the total number of pixels in the image and *i*, *j* the individual pixels.

$$PSNR \;= 20\; log_{10}\,(L) \;-\; 10\; log_{10}\,(MSE) \qquad \text{(Eq. 8)}$$

Supplementary Note 2 - Selecting a network for bacterial segmentation

Different DL networks were developed to perform semantic/instance segmentation of bioimages. The performance of these networks can vary strongly on the type of data (e.g. strains or microscopy technique) used.

The following considerations can help to decide for a particular network:

(i) Estimate the axial ratio of bacteria in the images. Small axial ratios (in our experience 1 -3; e.g. for slowly growing rod-shaped cells, stationary phase cells or cocci/ovococci) are ideal targets for StarDist independent of cell density.

(ii) Cells with axial ratios above ~4-5 or non-convex cells (e.g. fast-growing rod-shaped cells such as *E. coli* or *B. subtilis*) can be segmented with U-Net, CARE and pix2pix but require additional post-processing (thresholding and particle analysis). Examples of such processes are background subtraction, specific thresholding algorithms, pixel-based actions (e.g. dilation or erosion) active contour-based algorithms [3], or marker-based watershed segmentation. The workflow for each model differs slightly (see Supplementary Figure 1), which also influences the time required for data preparation, up-/downloading, and post-processing.

(iii) Prediction of multiple labels increases segmentation performance for densely growing cells. In this case, we suggest using the multi-label U-Net network that is trained on semantic images labelled for cell cytosol and boundaries.

(iv) To quickly get an impression of network suitability, we recommend transfer-learning using a few annotated images and a model pre-trained on images with similar parameters (pixel size, bacterial shape, image modality). Network performance should be evaluated using test data. Metrics such as IoU, Precision or Recall can be used for this purpose (see Supplementary Note 1) and are automatically calculated in the QC section of ZeroCostDL4Mic notebooks.

(v) There are many other segmentation approaches available that can be tested. For phase contrast images, for example, the MatLab-based SuperSegger [4] or Oufti [5] can be employed. DeepLearning networks such as DeepCell [6] or MISIC [7] can be used to segment and track cells at high density in phase contrast and for MISIC also in bright field images. Their implementation, however, might require more computational expertise or support from developers.

Supplementary Figures

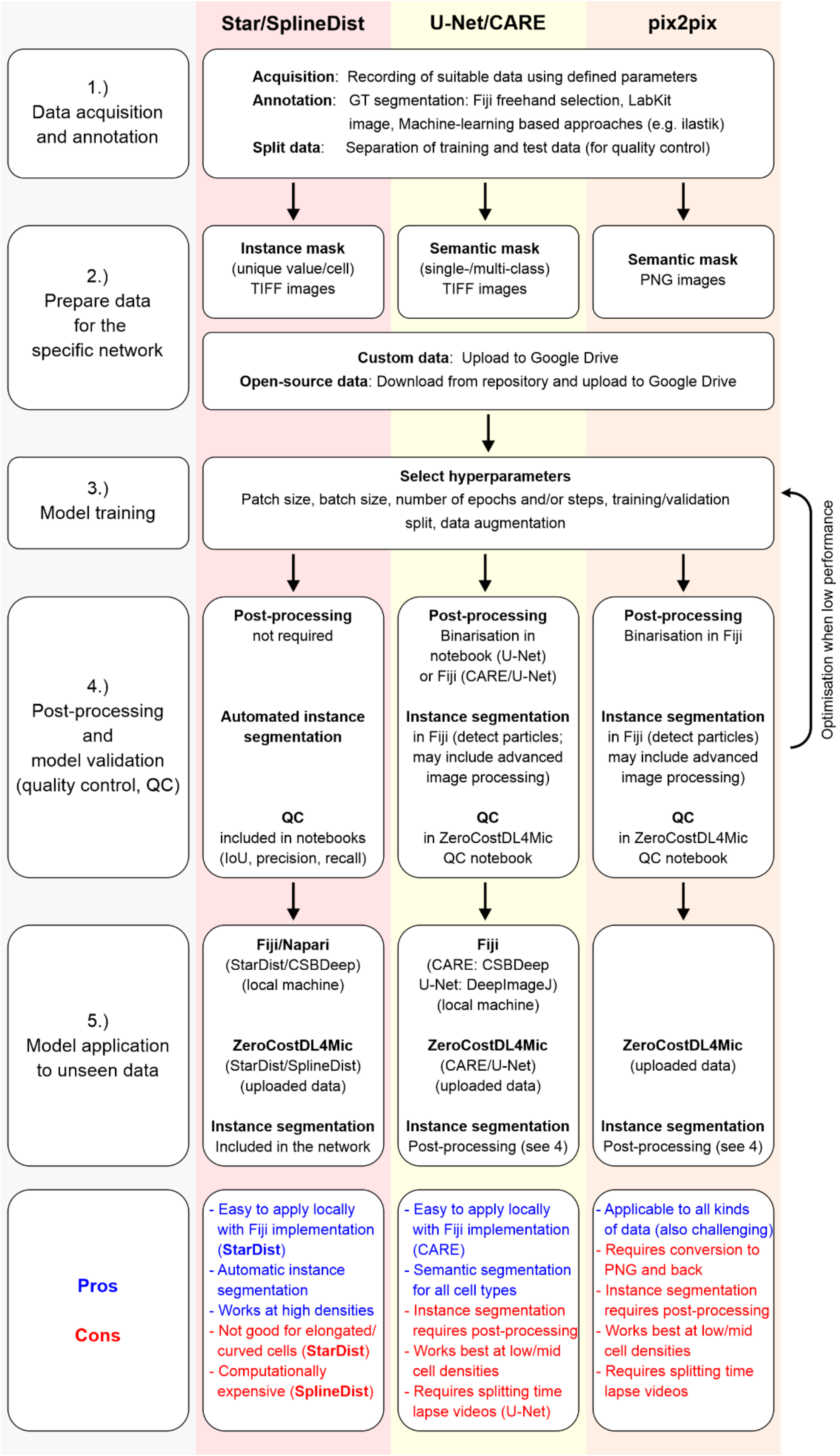

***Supplementary Figure 1**: Segmentation workflows using cloud implementations of different DL networks.*

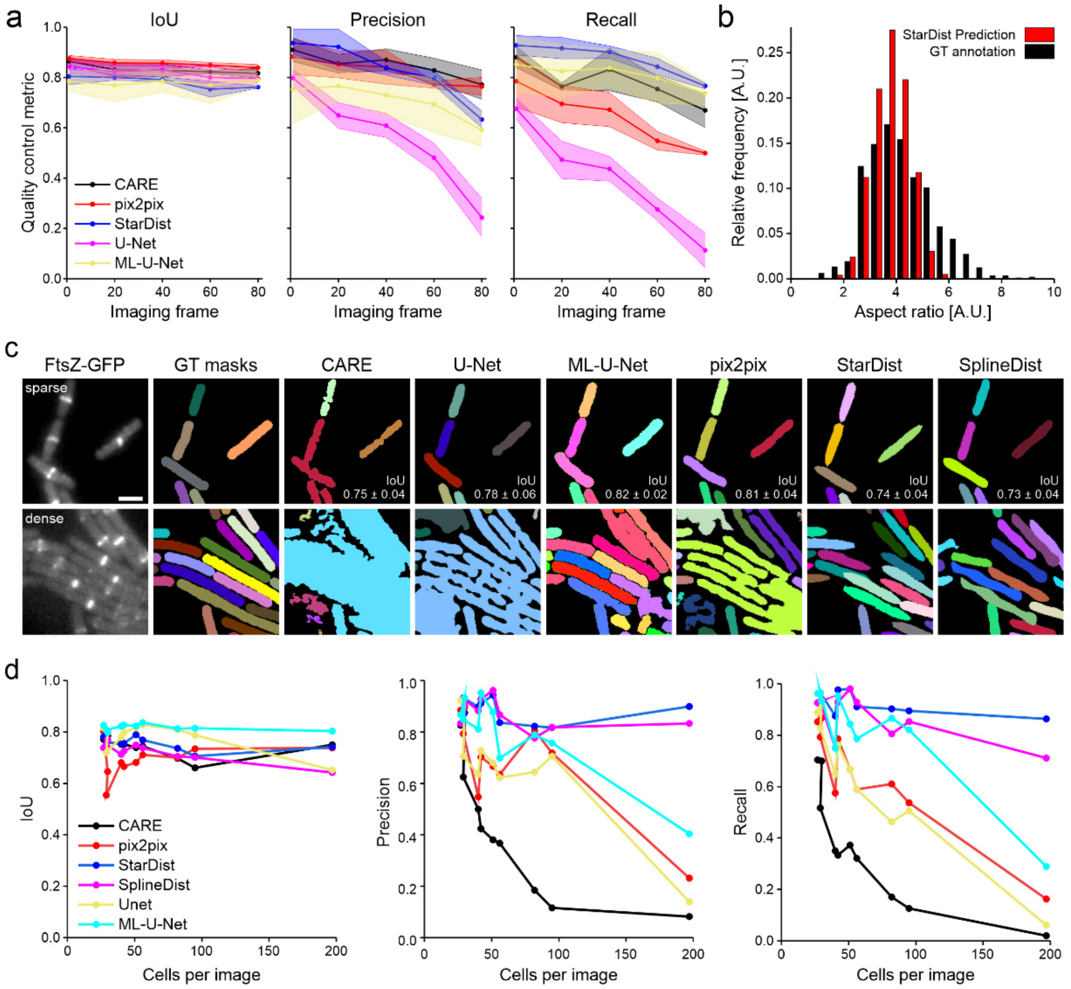

Supplementary Figure 2*: Performance of brightfield and fluorescence image segmentation using different deep learning approaches.* ***a*** *Intersection over Union (IoU), precision and recall of instance segmentations of brightfield time-lapse images of growing E. coli cells generated with different networks.* ***b*** *Axial ratio analysis of StarDist instance segmentation of E. coli bright field images.* ***c*** *Segmentation of FtsZ-GFP fluorescence images of living B. subtilis. IoU values were determined for the test data set (10 image pairs).* ***d*** *Intersection over Union (IoU), precision and recall of instance segmentations of living, GFP-FtsZ expressing, B. subtilis cells generated with different networks. Scale bar in* ***c*** *is 2 µm*

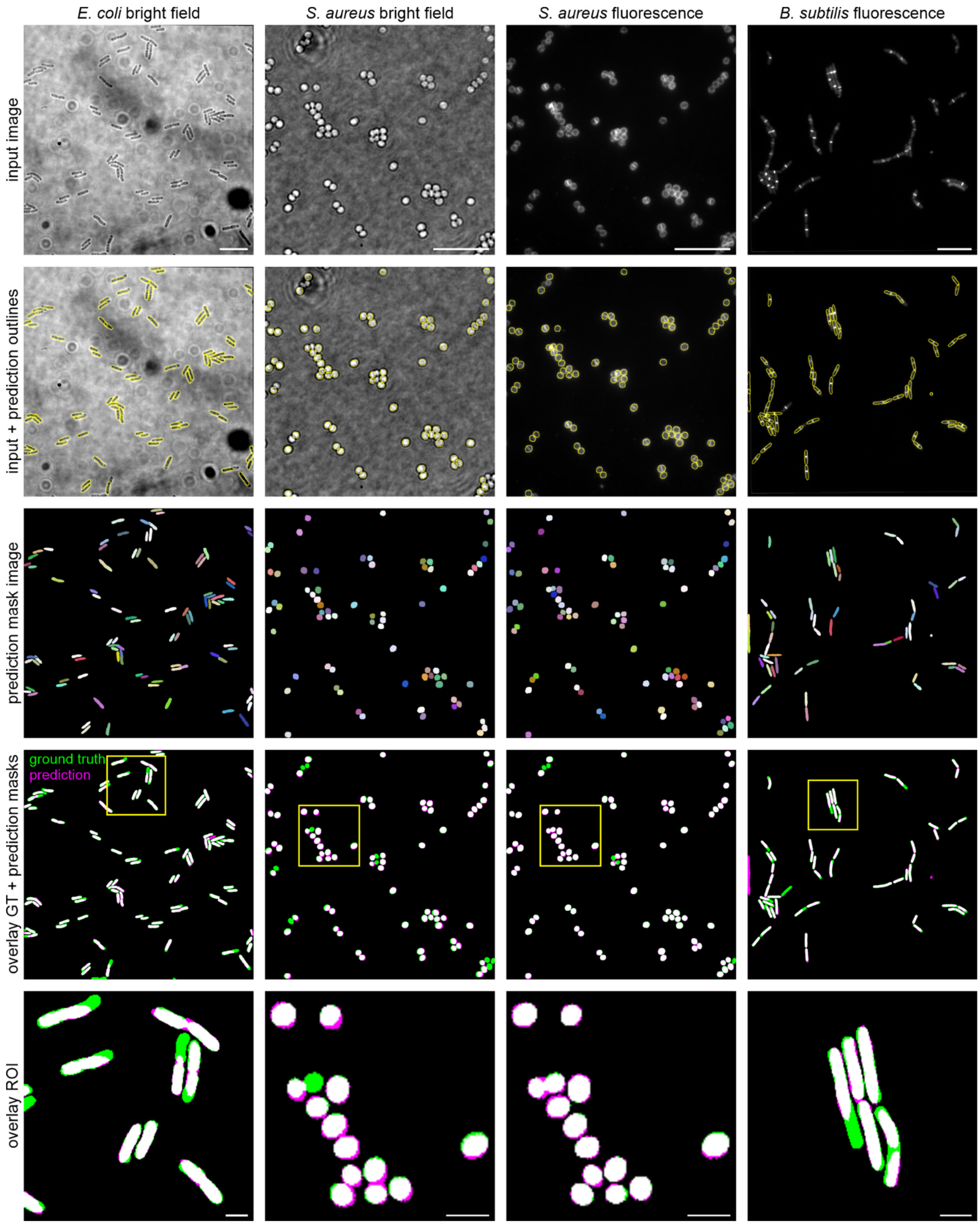

Supplementary Figure 3: *StarDist "master model" for segmentation of the four datasets provided in this work. The lowest panel shows insets of ground truth (GT) -prediction overlays marked by yellow rectangles in the overview image (second-last row). Scale bars are 10 µm (overviews) and 2 µm (ROIs).*

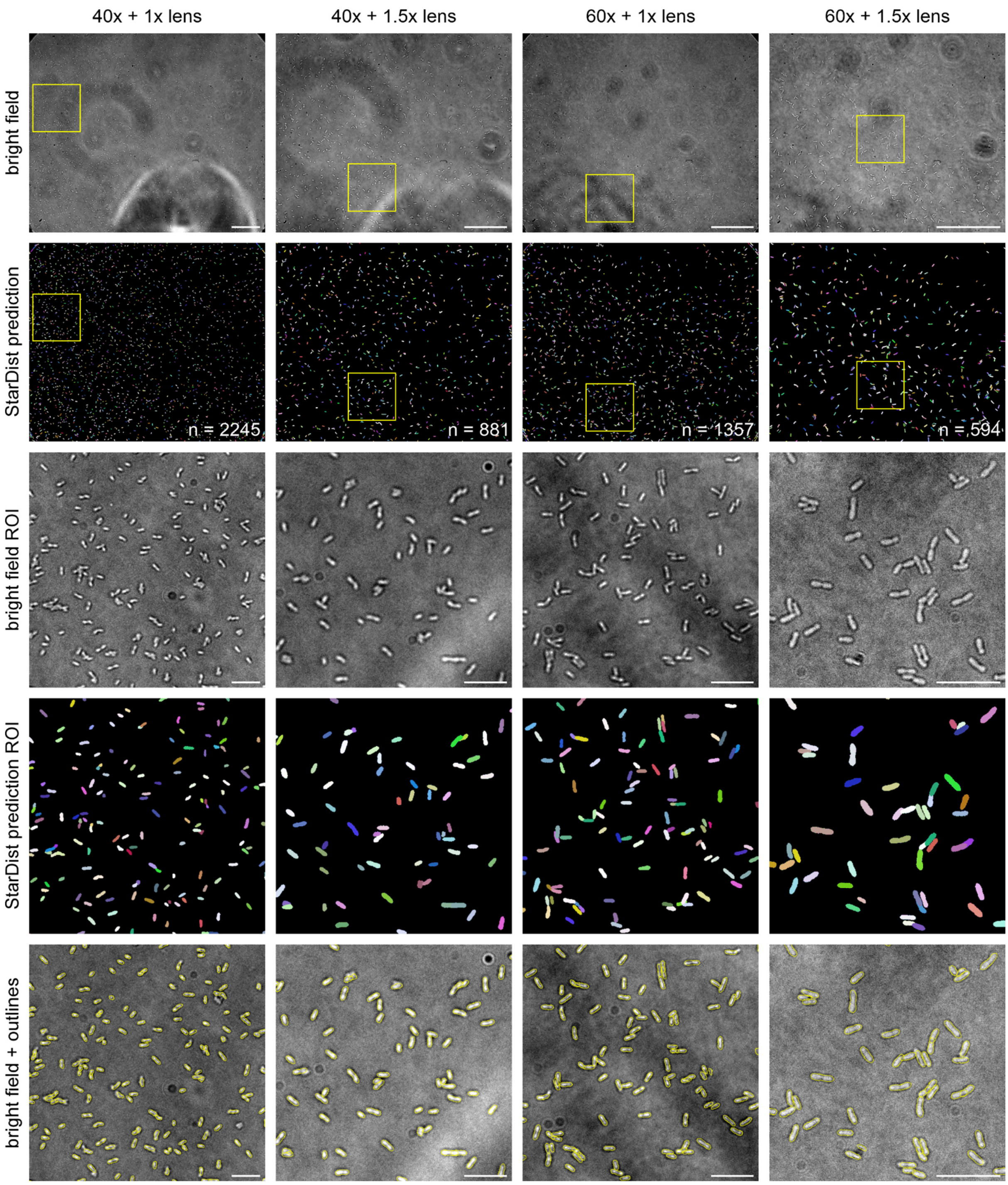

Supplementary Figure 4*: Agrobacterium tumefaciens large field of view StarDist segmentations. A 2D StarDist model was trained on pooled bright field data (512 x 512 px patches) acquired with different objectives and at different magnifications. This allows predicting a high cell number in large images also at challenging bright field conditions. Scale bars are 20 µm (overview images) and 10 µm (ROIs).*

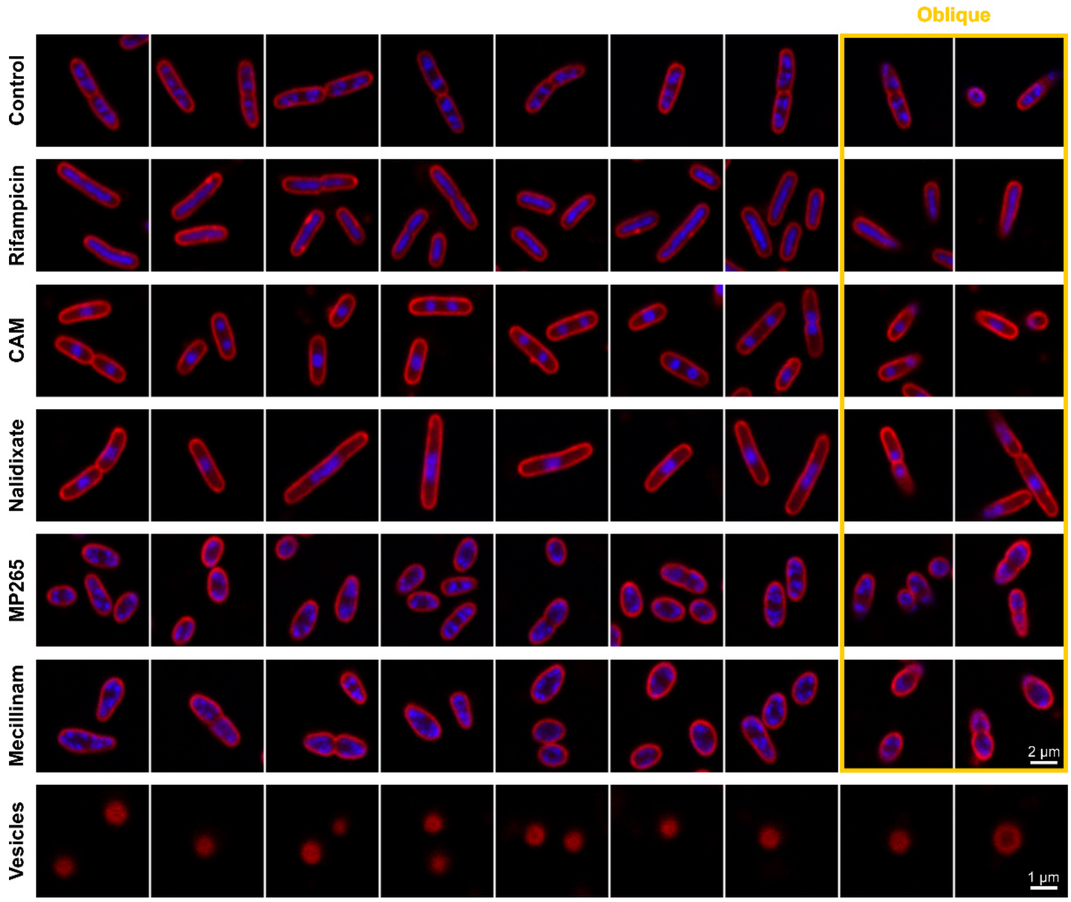

Supplementary Figure 5*: Examples for the different classes used in DL-assisted antibiotic phenotyping. Oblique cells represent incompletely attached cells and are thus included in all antibiotic treatments and in the control condition.*

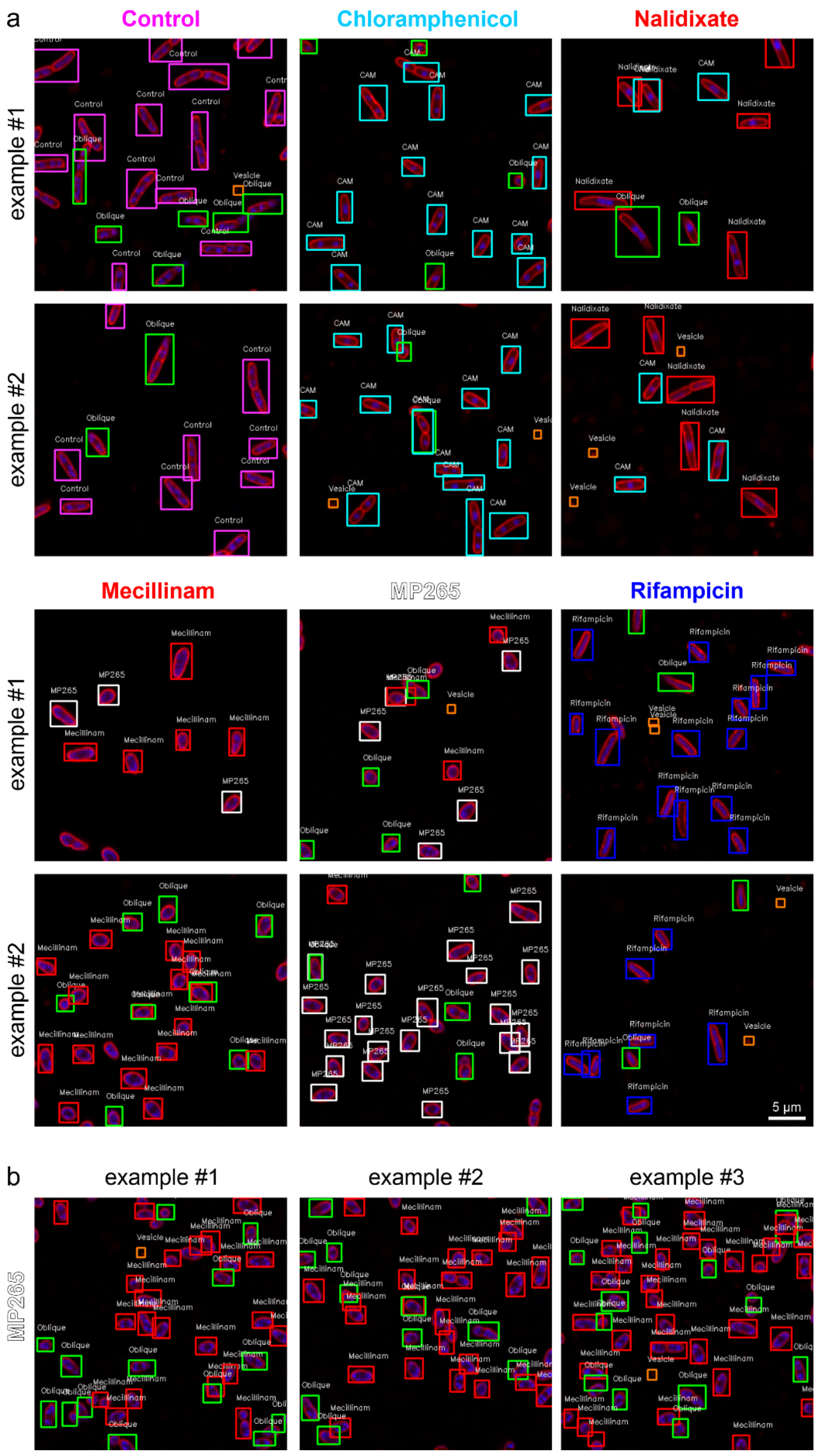

Supplementary Figure 6*: Antibiotic profiling model predictions.* ***a*** *Representative regions of interest showing object detection results on the single-treatment test dataset.* ***b*** *Application of a YOLOv2 model to identify shared mode-of-actions. The model was trained on a dataset without MP265-treated cells. Application of the model to images with MP265-treated cells predicts Mecillinam treatment (red bounding box) due to a similar phenotype.*

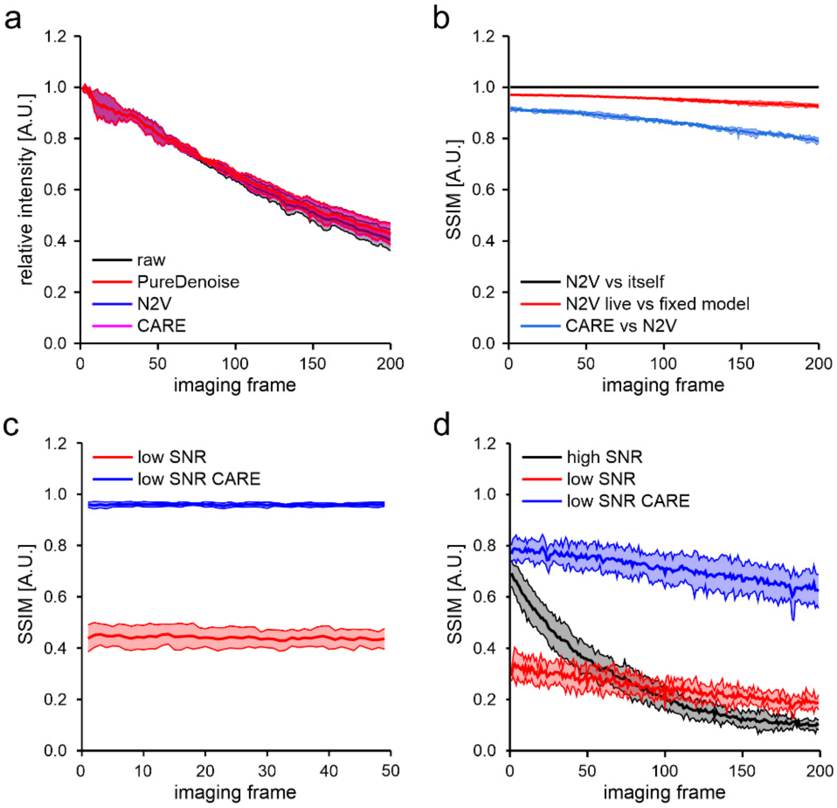

***Supplementary Figure 7**: Controls for DL-based image denoising. **a** Intensity over time for raw and denoised data. Denoising does not affect the relative intensities. **b** SSIM analysis of live-cell time series denoised with different approaches. Perfect similarity is given by a value of 1, as shown for SSIM analysis using the same time series (black line). A high similarity is observed between N2V models trained either on the fixed-cell dataset or on live-cell time series image frames. Time series denoised with CARE and N2V models also exhibit high SSIM values. Decreasing SSIM over imaging time probably arises from a better performance of supervised training (CARE) for images with lower SNR values, occurring at the end of the measurements due to photobleaching. **c** SSIM over time (subsequent image frames) of fixed cell time series, imaged under low SNR conditions (red) and denoised using CARE (blue). **d** SSIM over time (subsequent image frames) for high SNR (black, high laser power and 4x line averaging), low SNR (red, low laser power without averaging) and low SNR time series denoised with CARE (blue). Note that the low- and high-SNR data were determined from unpaired time series. Data represent the average value of at least 3 measurements and the shaded areas the respective standard deviation.*

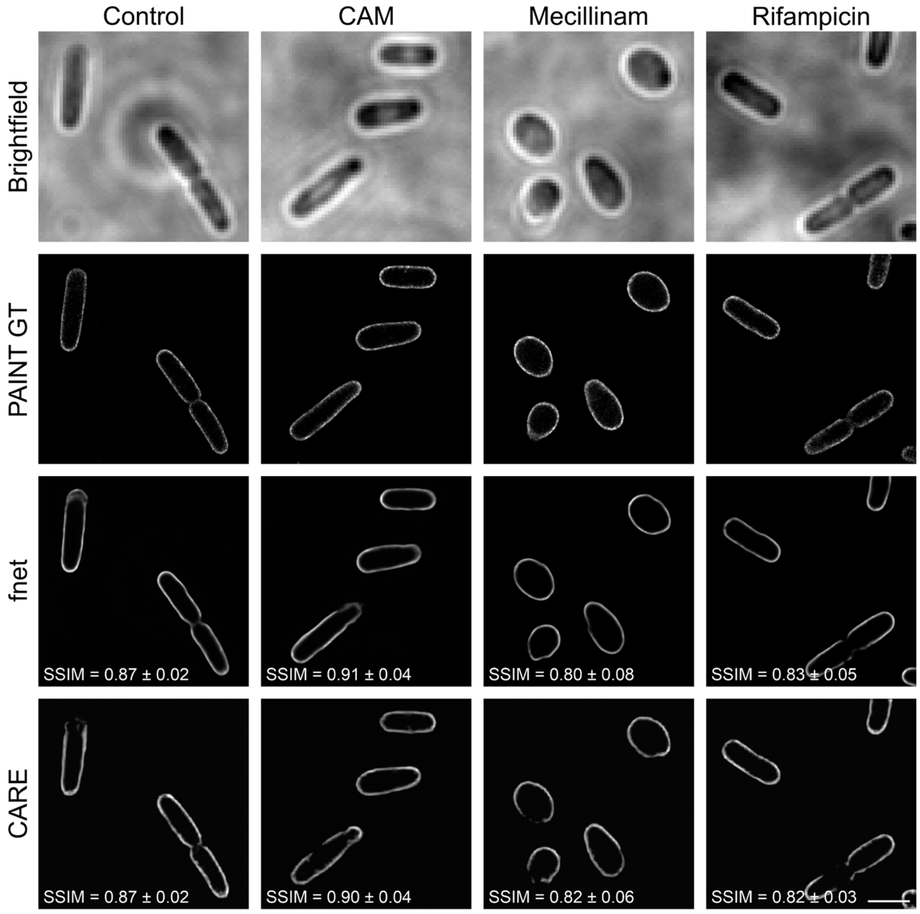

Supplementary Figure 8: *Artificial labelling of E. coli membranes under different treatments. PAINT super-resolution images were used for model training. fnet and CARE models can also be used to predict membranes from cells that were treated with different antibiotics. SSIM = structural similarity. Scale bar is 2 µm.*

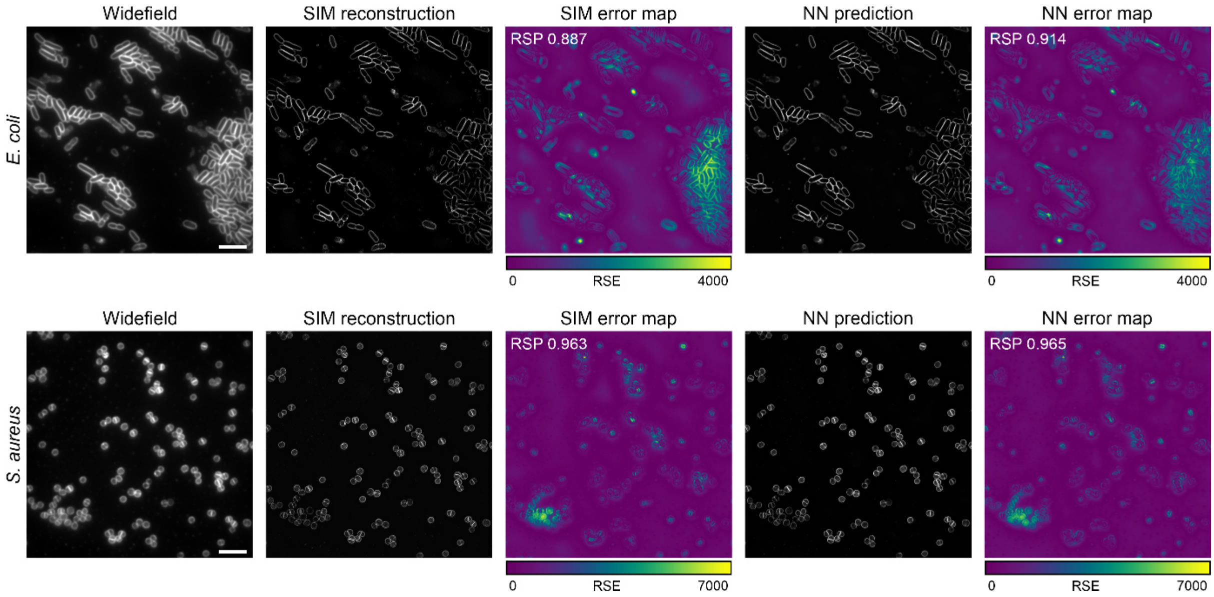

Supplementary Figure 9*: SQUIRREL analysis [8] of SIM reconstructions and SIM images predicted by the NN (CARE). Error maps show the resolution scaled error (RSE) calculated between the blurred super-resolution image and the widefield image. The resolution-scaled Pearson coefficient (RSP) represents a normalised quality metric, providing high values and thus high-quality reconstructions for both SIM images and NN predictions. Scale bars are 5 µm.*

Supplementary Tables

Supplementary Table 1: Dataset sizes and imaging modalities of the dataset provided in this study. Different test datasets were provided for 3b (stitched/individual images). BF: Bright field; WF: Widefield; Fl.: Fluorescence; SR: Super-resolution; PAINT: Point accumulation for imaging in nanoscale topography; SIM: structured illumination microscopy; FoV: Field of view.

Figure	Task	Species	Training images	Test images	Image size (px)	Image size (µm)	∅ objects/image	Imaging modality
2b	Segmentation	*S. aureus*	28	5	256 x 256	20.45 x 20.45	45 ± 34	BF / Fl. (WF)
2c	Segmentation	*E. coli*	19	15	1024 x 1024	81.05 x 81.05	81 ± 24	BF (WF)
2d	Segmentation	*B. subtilis*	80	10	1024 x 1024	66.56 x 66.56	53 ± 31	Fl. (WF)
2e	Segmentation	*E. coli*	33	12	512 x 512	54.27 x 54.27	58 ± 41	BF (WF)
3a	Object detection	*E. coli*	25	15	512 x 512	81.05 x 81.05	52 ± 10	BF (WF) (large FoV)
3b	Object detection	*E. coli*	153	32/50	400 x 400	33.6 x 33.6	15 ± 8	Fl. (confocal)
4a	Denoising	*E. coli*	28	2	512 x 512	23.11 x 23.11	26 ± 19	Fl. (confocal)
4e	Denoising	*E. coli*	30	5	512 x 512	23.11 x 23.11	15 ± 8	Fl. (confocal)
4h	Denoising	*B. subtilis*	3	-	1024 x 1024	66.56 x 66.56	110 ± 13	Fl. (WF)
5a	Artificial labelling	*E. coli*	56	8	512 x 512	27.14 x 27.14	15 ± 7	BF / Fl (WF)
5a	Artificial labelling	*E. coli*	33	20	512 x 512	10.01 x 10.01	1 - 4	BF / SR (PAINT)
6a	SR prediction	*E. coli*	55	5	512 x 512	40.96 x 40.96	~ 50 - 300	SR (SIM)
6b	SR prediction	*S. aureus*	94	5	512 x 512	40.96 x 40.96	96 ± 52	SR (SIM)

Supplementary Table 2: List of training/test data and models shared via Zenodo

Figure	Task	Species	DOI	Type
2b	Segmentation	*S. aureus*	10.5281/zenodo.5550933	Training/test data
2c	Segmentation	*E. coli*	10.5281/zenodo.5550935	Training/test data
2d	Segmentation	*B. subtilis*	10.5281/zenodo.5550968	Training/test data
2d	Segmentation	*B. subtilis*	10.5281/zenodo.5639253	Training/test data + Multilabel-U-Net model
2e	Segmentation	*E. coli*	10.5281/zenodo.6400327	Trainin/test data + StarDist 2D model
S2	Segmentation	Mixed	10.5281/zenodo.5551009	Training/test data + StarDist 2D model
3a	Object detection	*E. coli*	10.5281/zenodo.5551016	Training/test data + YOLOv2 model
3b	Object detection	*E. coli*	10.5281/zenodo.5551057	Training/test data + YOLOv2 model
4a	Denoising	*E. coli*	10.5281/zenodo.5551112	Training/test data + CARE 2D model
4e	Denoising	*E. coli*	10.5281/zenodo.6460867	Training/test data + CARE 2D model
4h	Denoising	*B. subtilis*	10.5281/zenodo.5551135	Training/test data
5a	Artificial labelling	*E. coli*	10.5281/zenodo.5551123	Training/test data + fnet/CARE models
6a	SR prediction	*E. coli*	10.5281/zenodo.5551153	Training/test data + CARE model
6b	SR prediction	*S. aureus*	10.5281/zenodo.5551141	Training/test data + CARE model

Supplementary Table 3: Training parameters for segmentation networks

Organism	Mode	Network	Images (train/test)	Epochs	Steps	Image size	Patch size	Batch size	LR	% Valid.	Augmentation	Misc.	Train time
S. aureus	Brightfield	StarDist	28/5	400	22	256 x 256 px²	256 x 256 px²	4	0.0003	20	4x (flip/rot.)	grid 1, 32 rays	24 min 53 sec
	Fluorescence	StarDist	28/5	400	24	256 x 256 px²	256 x 256 px²	4	0.0003	20	4x (flip/rot.)	grid 1, 32 rays	53 min 27 sec
E. coli	Brightfield	U-Net	19/15	100	30	1024 x 1024 px²	512 x512 px²	2	0.0003	10	Flip/Shear		23 min
		ML-U-Net	19/15	500	17	1024 x 1024 px²	512 x512 px²	4	0.0003	10	4x (all options)	2 pooling steps	1 h 8 min
		CARE	19/15	100	300	1024 x 1024 px²	128 x 128 px²	8	0.0004	20	4x (flip/rot.)		42 min 16 sec
		StarDist	19/15	200	30	1024 x 1024 px²	512 x512 px²	2	0.0003	20	4x (flip/rot.)	grid 1, 80 rays	45 min 23 sec
		pix2pix	19/15	500	n.a.	1024 x 1024 px²	256 x 256 px²	1	0.0002	n.a.	4x (flip/rot.)		47 min 57 sec
B. subtilis	Fluorescence	U-Net	80/10	100	29	1024 x 1024 px²	512 x 512 px²	10	0.001	10	4x (flip/rot.)		47 min
		ML-U-Net	80/10	200	83	1024 x 1024 px²	256 x 256 px²	8	0.0005	10	4x (all options)	3 pooling steps	1 h 10 min
		CARE	80/10	50	2000	1024 x 1024 px²	64 x 64 px²	16	0.001	20	4x (flip/rot.)		1h 7 min
		StarDist	80/10	400	80	1024 x 1024 px²	512 x 512 px²	4	0.0003	10	4x (flip/rot.)	grid 2, 64 rays	4 h 20 min
		SplineDist	32/10	800	25	512 x 512 px²	256 x 256 px²	4	0.0003	20	4x (flip/rot.)	grid 2	5 h 11 min
		pix2pix	80/10	1000	n.a.	1024 x 1024 px²	256 x 256 px²	1	0.0002	n.a.	4x (flip/rot.)		5h 50 min
All above	mixed model	StarDist	155/35	200	120	all above	256 x 256 px²	4	0.0003	10	3x (flip/rot.)	grid 2, 64 rays	44 min 34 sec
E. coli	Brightfield (stat. Phase)	StarDist	33/12	200	60	512 x 512 px²	512 x 512 px²	2	0.0003	10	4x (flip/rot.)	grid 1, 80 rays	1 h 48 min

Supplementary Table 4: Segmentation performance of StarDist generalist model (G) and specialist models (S)

Dataset	IoU (G)	IoU (S)	Precision (G)	Precision (S)	Recall (G)	Recall (S)
E. coli bright field	0.74 ± 0.03	0.78 ± 0.03	0.88 ± 0.08	0.87 ± 0.07	0.82 ± 0.12	0.83 ± 0.12
S. aureus bright field	0.71 ± 0.02	0.64 ± 0.01	0.89 ± 0.04	0.90 ± 0.02	0.99 ± 0.02	0.87 ± 0.03
S. aureus fluorescence	0.86 ± 0.01	0.91 ± 0.03	0.95 ± 0.03	0.98 ± 0.02	0.99 ± 0.02	1.00 ± 0.01
B. subtilis fluorescence	0.69 ± 0.04	0.76 ± 0.03	0.82 ± 0.09	0.92 ± 0.04	0.73 ± 0.09	0.88 ± 0.55

Supplementary Table 5: YOLOv2 model training parameters. FNP = False negative penalty, FPP = False positive penalty, PSP = Position and size penalty, FCP = False class penalty

Dataset	Images (train/test)	Epochs	Image size	Train cycles	Batch size	LR	% Valid.	Augmentation	Penalties (FNP, FPP, PSP, FCP)	Training time
Growth stage (large FoV)	25/15	100	512 x 512 px²	4	4	0.0003	20	4x (flip/rot.)	5, 1, 3, 3	1 h 31 min
Growth stage (small FoV)	100/60	100	256 x 256 px²	4	8	0.0003	20	4x (flip/rot.)	5, 1, 3, 3	34 min
Antibiotic profiling	153/32 (50)	100	400 x 400 px²	4	16	0.001	20	8x (flip/rot.)	4, 2, 1, 2	2 h 33 min

Supplementary Table 6: Performance of YOLOv2 models for growth stage prediction, trained on differently sized field of views (FOV). mean average precision (mAP) values were calculated from the individual classes' average precision (AP) values.

FOV	Class	Recall	Precision	AP	mAP
Large (512 x 512 px²)	Rod	0.442	0.621	0.321	**0.386**
	Dividing	0.414	0.760	0.358	
	Microcolony	0.567	0.567	0.479	
Small (256 x 256 px²)	Rod	0.746	0.712	0.643	**0.667**
	Dividing	0.657	0.722	0.589	
	Microcolony	0.804	0.755	0.771	

Supplementary Table 7: Supplier information and experimental conditions for the antibiotics used in this study

Antibiotic	Supplier	Cat. #	Working concentration	Exposure time
MP265	Alfa Aesar	B25362	25 µM	60 min
Mecillinam	Sigma Aldrich	33447	2 µg/ml	60 min
Rifampicin	Sigma Aldrich	R3501	100 µg/ml	60 min
Chloramphenicol	Sigma Aldrich	C0378	50 µg/ml	60 min
Nalidixate	Alfa Aesar	J63550	50 µg/ml	30 min

Supplementary Table 8: Performance of the YOLOv2 antibiotic profiling model on different types of test datasets.

Test data	Class	# of objects in training dataset	# of objects in test dataset	Recall	Precision	AP score	mAP
stitched images	Rifampicin	430	102	0.920	0.928	0.879	**0.662**
	Oblique	535	93	0.871	0.750	0.769	
	Nalidixate	87	58	0.707	0.854	0.641	
	Mecillinam	327	91	0.703	0.744	0.605	
	CAM	264	45	0.978	0.620	0.754	
	Vesicle	207	67	0.284	0.594	0.207	
	Control	181	66	0.939	0.912	0.914	
	MP265	265	78	0.654	0.671	0.526	
individual treatment	Rifampicin	430	55	0.891	0.907	0.860	**0.690**
	Oblique	535	161	0.745	0.876	0.713	
	Nalidixate	87	64	0.672	0.796	0.602	
	Mecillinam	327	66	0.758	0.769	0.694	
	CAM	264	65	0.938	0.635	0.759	
	Vesicle	207	119	0.353	0.600	0.257	
	Control	181	61	0.984	0.822	0.944	
	MP265	265	76	0.789	0.800	0.692	

Supplementary Table 9: Model training parameters for image denoising

Organism	Network	Images (train/test)	Epochs	Steps	Image size	Patch size (#/img)	Batch size	LR	% Valid.	Augmentation	Train time
E. coli	CARE	28/2	100	600	512 x 512 px²	64 x 64 px² (50)	8	0.0004	10	4x (flip/rot.)	30 min 34 s
	N2V	28/2	200	101	512 x 512 px²	64 x 64 px² (50)	128	0.0004	10	4x (flip/rot.)	41 min
B. subtilis	N2V	3/n.a.	200	44	1024 x 1024 px²	64 x 64 px² (500)	128	0.0004	10	4x (flip/rot.)	18 min

Supplementary Table 10: Model training parameters for artificial labelling

Organism	Imaging Mode	Scaling	Network	Images (train/test)	Epochs	Steps	Image size	Patch size (#/img)	Batch size	LR	% Valid.	Augmentation	Train time
E. coli	Widefield (108 + 158 nm/px)	none	fnet	56/8	n.a.	200000	256 x 256 px²	128 x 128 px² (1)	2	0.001	10	4x (flip/rot.)	1 h 43 min
			CARE	56/8	300	200	256 x 256 px²	64 x 64 px² (4)	4	0.0004	10	4x (flip/rot.)	57 min
	PAINT (158 nm/px)	8x	fnet	33/20	n.a.	200000	512 x 512 px²	128 x 128 px² (1)	4	0.0004	10	4x (flip/rot.)	2 h 27 min
			CARE	33/20	300	100	512 x 512 px²	256 x 256 px² (1)	4	0.0004	10	4x (flip/rot.)	1 h 33 min

Supplementary Table 11: Model training parameters for super-resolution prediction

Organism	Scaling	Network	Images (train/test)	Epochs	Steps	Image size	Patch size (#/img)	Batch size	LR	% Valid.	Augmentation	Train time
E. coli	2x	CARE	55/5 (link)	300	2500	1024 x 1024 px²	80 x 80 px² (100)	8	0.0004	10	4x (flip/rot.)	6 h 6 min
S. aureus	2x	CARE	94/5 (link)	300	3000	1024 x 1024 px²	80 x 80 px² (100)	8	0.0004	10	4x (flip/rot.)	7 h 23 min

Supplementary Videos:

Supplementary Video 1: Application of a StarDist model trained using the ZerCostDL4Mic platform in the StarDist Fiji plugin.

Supplementary Video 2: StarDist segmentation of S. aureus time-lapse data at high cell density. Yellow lines outline segmented cells.

Supplementary Video 3: Segmentation and tracking of E. coli time-lapse data using a multilabel-U-Net and TrackMate. Boundaries are coloured according to the lineages.

Supplementary Video 4: Segmentation and tracking of E. coli time-lapse data using StarDist and TrackMate. Boundaries are coloured according to the lineages.

Supplementary Video 5: YOLOv2 object detection of different growth stages in E. coli time-lapse data. The model was trained to detect non-dividing cells (blue bounding boxes), dividing cells (green bounding boxes) or microcolonies (red bounding boxes) using the entire field of view (80x80 µm²).

Supplementary Video 6: YOLOv2 object detection of different growth stages in E. coli time-lapse data. The model was trained to detect non-dividing cells (blue bounding boxes), dividing cells (green bounding boxes) or microcolonies (4+ cells in close contact, red bounding boxes) using a ROI of 40x40 µm².

Supplementary Video 7: Denoising of E. coli nucleoid dynamics. Confocal time-lapse videos of E. coli cells expressing H-NS-mScarlet protein fusion from the native locus were denoised using PureDenoise, Noise2Void or CARE.

Supplementary Video 8: Denoising of E. coli MreB dynamics. Confocal time-lapse videos of E. coli cells expressing a MreB-sfGFP^{sw} protein fusion from the native locus were denoised using PureDenoise, Noise2Void or CARE. Tracking was performed with TrackMate. 20 frame track segments are shown.

Supplementary Video 9: Self-supervised denoising of FtsZ-GFP dynamics in vertically aligned B. subtilis cells using Noise2Void. The false-coloured panel is shown for visualisation. Scale bar is 1 µm.

Supplementary Video 10: Artificial labelling of E. coli bright field time series using fnet. The fnet 2D model was trained using paired bright field and super-resolution PAINT images.

Supplementary Video 11: Prediction of super-resolution SIM images from widefield fluorescence images. Live S. aureus cells were stained with Nile Red. The right panel shows the overlay between SIM reconstructions (green) and the CARE 2D prediction (magenta).

Supplementary References:

1. von Chamier, L. *et al.* Democratising deep learning for microscopy with ZeroCostDL4Mic. *Nat. Commun.* **12**, 1–18 (2021).

2. Wang, Z., Bovik, A. C., Sheikh, H. R. & Simoncelli, E. P. Image quality assessment: From error visibility to structural similarity. *IEEE Trans. Image Process.* **13**, 600–612 (2004).

3. Kass, M., Witkin, A. & Terzopoulos, D. Snakes: Active contour models. *Int. J. Comput. Vis.* **1**, 321–331 (1988).

4. Stylianidou, S., Brennan, C., Nissen, S. B., Kuwada, N. J. & Wiggins, P. A. SuperSegger: robust image segmentation, analysis and lineage tracking of bacterial cells. *Mol. Microbiol.* **102**, 690–700 (2016).

5. Paintdakhi, A. *et al.* Oufti: An integrated software package for high-accuracy, high-throughput quantitative microscopy analysis. *Mol. Microbiol.* **99**, 767–777 (2016).

6. Van Valen, D. A. *et al.* Deep Learning Automates the Quantitative Analysis of Individual Cells in Live-Cell Imaging Experiments. *PLoS Comput. Biol.* **12**, 1–24 (2016).

7. Panigrahi, S. *et al.* Misic, a general deep learning-based method for the high-throughput cell segmentation of complex bacterial communities. *Elife* **10**, 1–18 (2021).

8. Culley, S. *et al.* Quantitative mapping and minimization of super-resolution optical imaging artifacts. *Nat. Methods* **15**, 263–266 (2018).
