# Supplementary material for: DeepBacs for multi-task bacterial image analysis using open-source deep learning approaches
Source: Commun Biol. 2022 Jul 9;5:688. doi: 10.1038/s42003-022-03634-z (PMC9271087; doi:10.1038/s42003-022-03634-z)
Supplement: Supplementary file 3 — Description of Additional Supplementary Files [file 42003_2022_3634_MOESM3_ESM.pdf]

Description of Additional Supplementary Files

File name: Supplementary Data 1
Description: Source data behind the graphs and statistics in the paper.

File name: Supplementary Video 1
Description: Application of a StarDist model trained using the ZerCostDL4Mic platform in the StarDist Fiji plugin.

File name: Supplementary Video 2
Description: StarDist segmentation of S. aureus time-lapse data at high cell density. Yellow lines outline segmented cells.

File name: Supplementary Video 3
Description: Segmentation and tracking of E. coli time-lapse data using a multilabel-U-Net and TrackMate. Boundaries are coloured according to the lineages.

File name: Supplementary Video 4
Description: Segmentation and tracking of E. coli time-lapse data using StarDist and TrackMate. Boundaries are coloured according to the lineages.

File name: Supplementary Video 5
Description: YOLOv2 object detection of different growth stages in E. coli time-lapse data. The model was trained to detect non-dividing cells (blue bounding boxes), dividing cells (green bounding boxes) or microcolonies (red bounding boxes) using the entire field of view (80x80 μm^2).

File name: Supplementary Video 6
Description: YOLOv2 object detection of different growth stages in E. coli time-lapse data. The model was trained to detect non-dividing cells (blue bounding boxes), dividing cells (green bounding boxes) or microcolonies (4+ cells in close contact, red bounding boxes) using a ROI of 40x40 μm^2.

File name: Supplementary Video 7
Description: Denoising of E. coli nucleoid dynamics. Confocal time-lapse videos of E. coli cells expressing H-NS-mScarlet protein fusion from the native locus were denoised using PureDenoise, Noise2Void or CARE.

File name: Supplementary Video 8
Description: Denoising of E. coli MreB dynamics. Confocal time-lapse videos of E. coli cells expressing a MreB-sfGFPsw protein fusion from the native locus were denoised using PureDenoise, Noise2Void or CARE. Tracking was performed with TrackMate. 20 frame track segments are shown.

File name: Supplementary Video 9
Description: Self-supervised denoising of FtsZ-GFP dynamics in vertically aligned B. subtilis cells using Noise2Void. The false-coloured panel is shown for visualisation. Scale bar is 1 µm.

File name: Supplementary Video 10
Description: Artificial labelling of E. coli bright field time series using fnet. The fnet 2D model was trained using paired bright field and super-resolution PAINT images.

File name: Supplementary Video 11
Description: Prediction of super-resolution SIM images from widefield fluorescence images. Live S.

aureus cells were stained with Nile Red. The right panel shows the overlay between SIM reconstructions (green) and the CARE 2D prediction (magenta).
