# Supplementary material for: DeepBacs for multi-task bacterial image analysis using open-source deep learning approaches
Source: Commun Biol. 2022 Jul 9;5:688. doi: 10.1038/s42003-022-03634-z (PMC9271087; doi:10.1038/s42003-022-03634-z)
Supplement: Supplementary file 15 — Supplementary Data 1 [file 42003_2022_3634_MOESM15_ESM.zip › Figure_2/Figure_2.pdf]

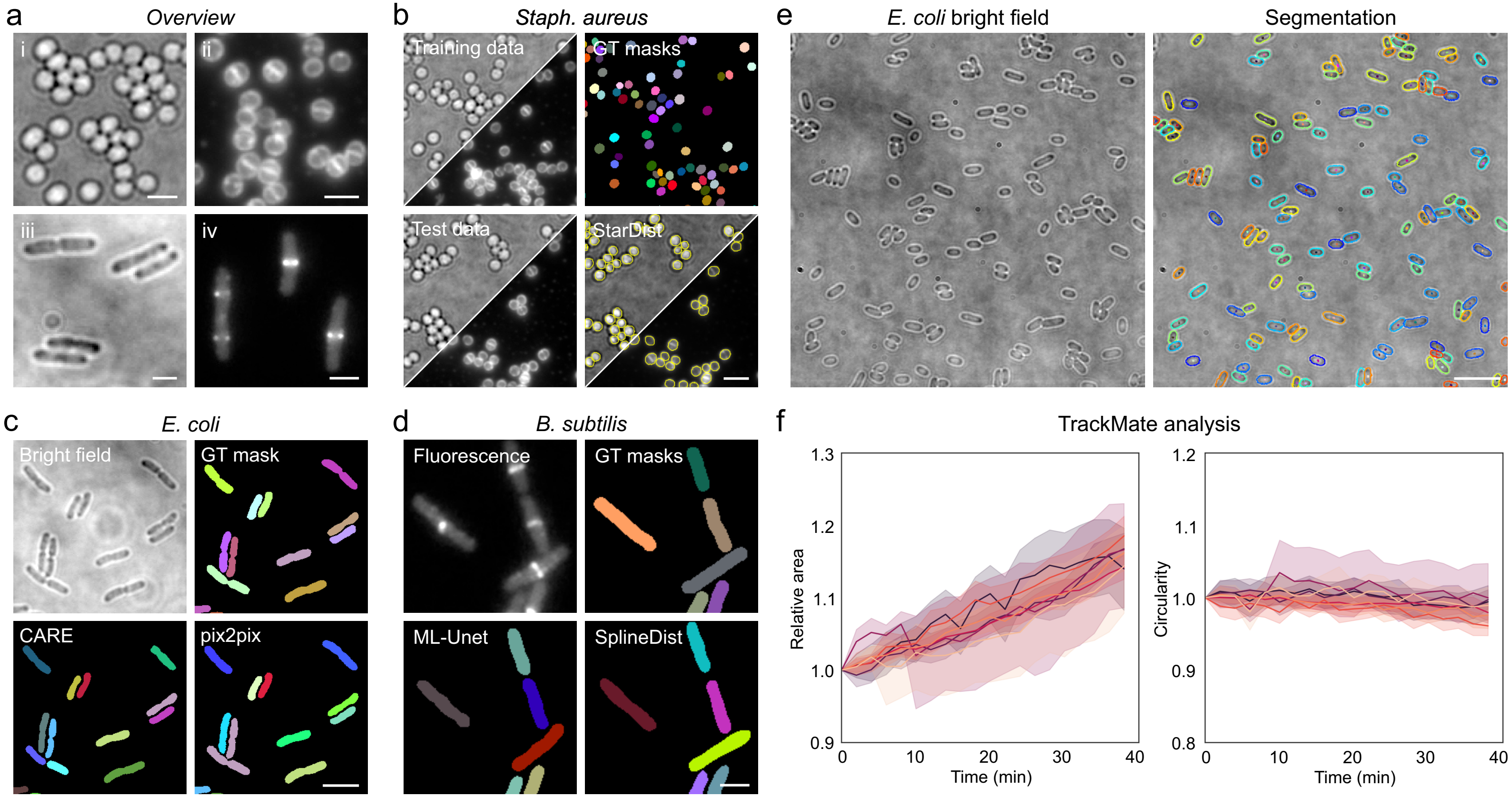

a
Overview
i
ii
iii
iv
b
Staph. aureus
Training data
GT masks
Test data
StarDist
c
E. coli
Bright field
GT mask
CARE
pix2pix
d
B. subtilis
Fluorescence
GT masks
ML-Unet
SplineDist
e
E. coli bright field
Segmentation
f
TrackMate analysis
Relative area
1.3
1.2
1.1
1.0
0.9
Time (min)
0
10
20
30
40
Circularity
1.2
1.1
1.0
0.9
0.8
