# Supplementary material for: DeepBacs for multi-task bacterial image analysis using open-source deep learning approaches
Source: Commun Biol. 2022 Jul 9;5:688. doi: 10.1038/s42003-022-03634-z (PMC9271087; doi:10.1038/s42003-022-03634-z)
Supplement: Supplementary file 15 — Supplementary Data 1 [file 42003_2022_3634_MOESM15_ESM.zip › Figure_3/Antibiotic_phenotyping/YOLOv2_Model_reports/All_antibiotic/M1_QC_report.pdf]

Quality Control report for YOLOv2 model (M2_new_DNA_membr_100ep_batch_16_train_4_aug_8_LR_0.001_val_20_FPP2_FNP4_FCP_2_full_YOLO)

Date and Time: 2021-02-19 07:03

Development of Training Losses

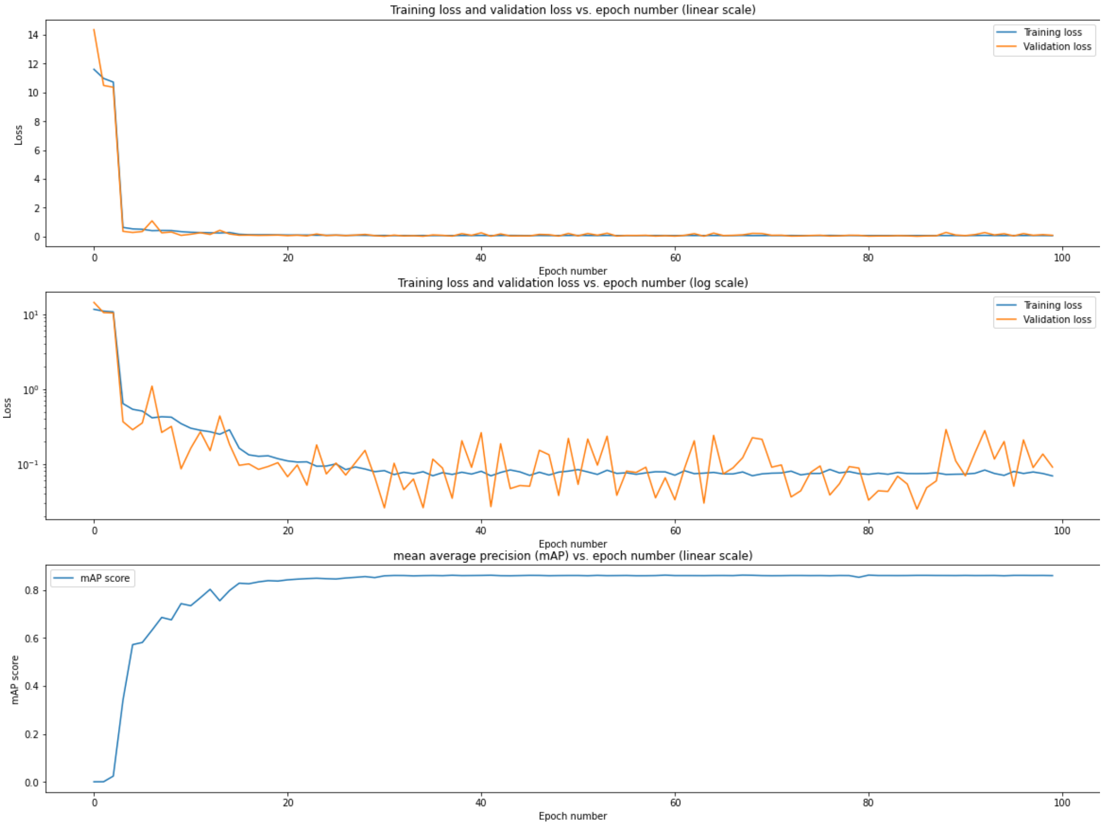

P-R curves for test dataset

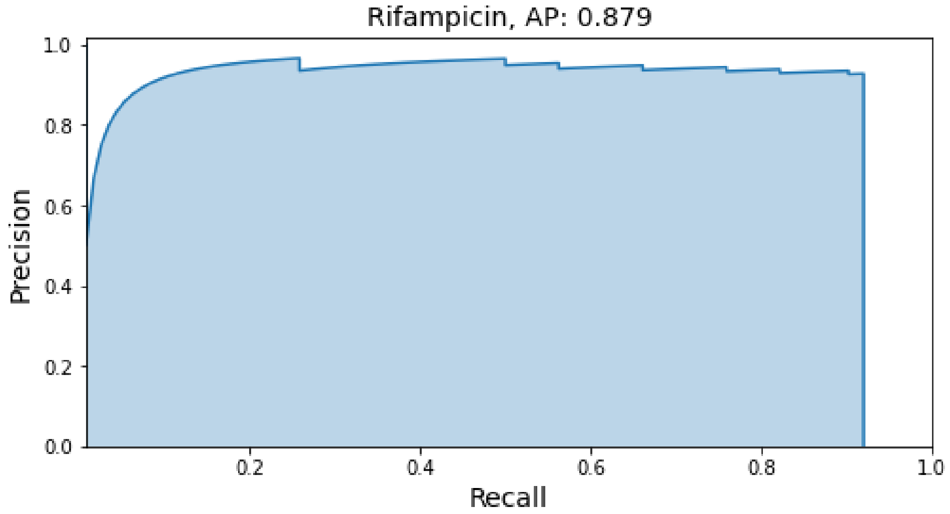

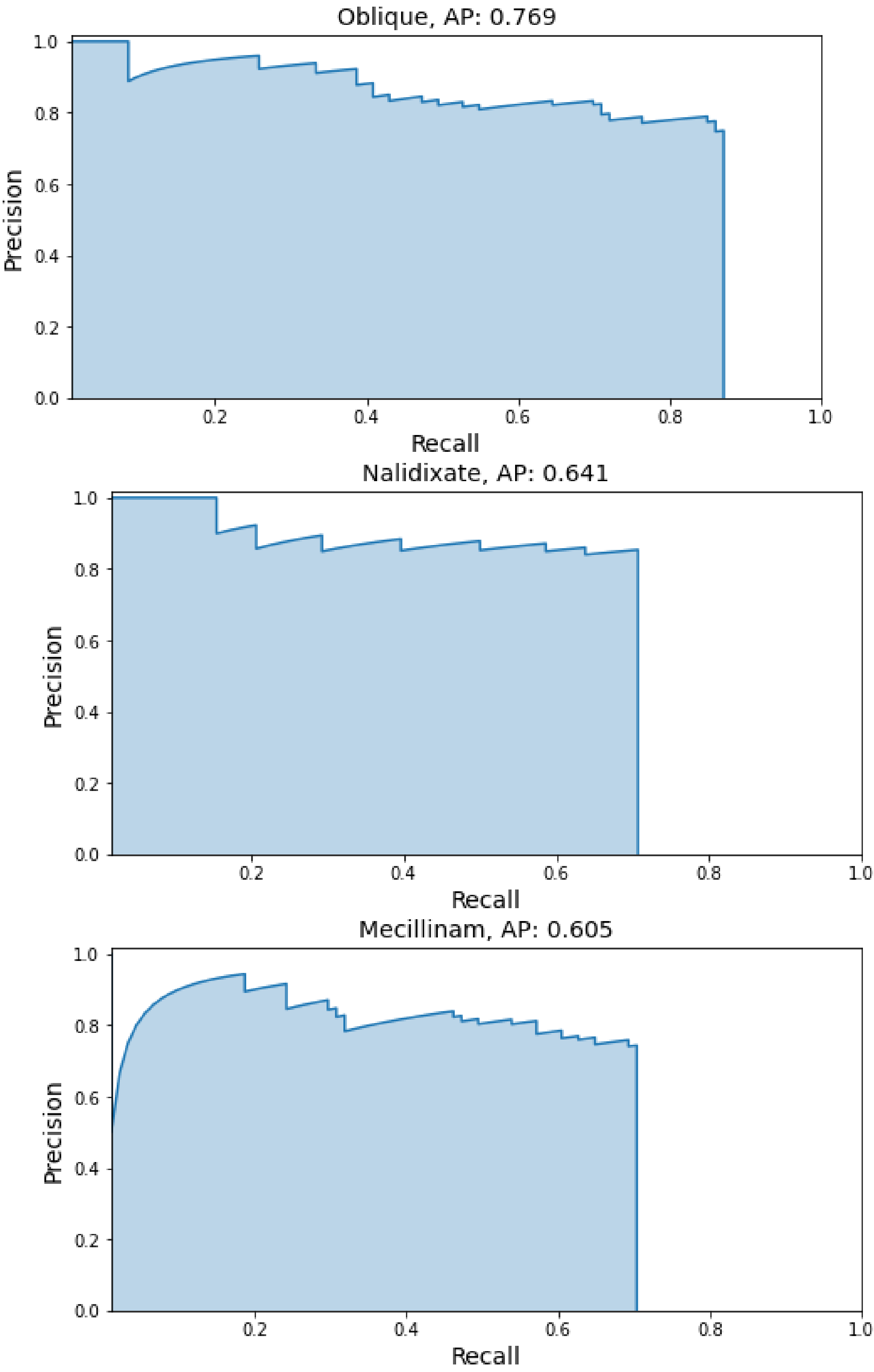
Oblique, AP: 0.769
Precision
Recall
Nalidixate, AP: 0.641
Precision
Recall
Mecillinam, AP: 0.605
Precision
Recall

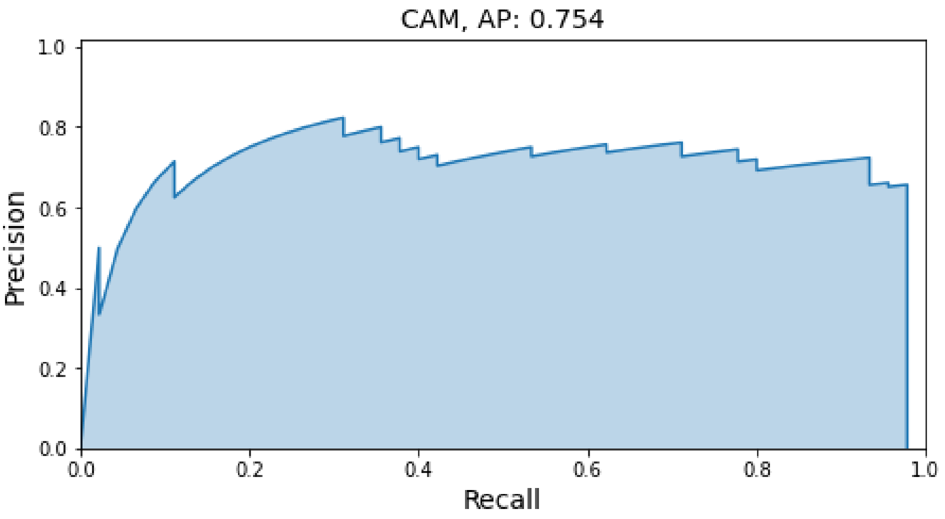
CAM, AP: 0.754
Precision
Recall
0.0
0.2
0.4
0.6
0.8
1.0

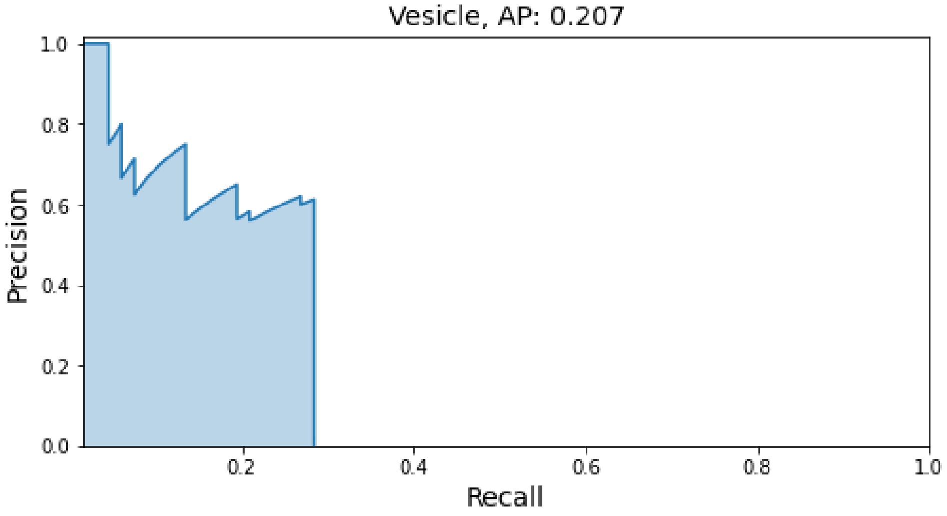
Vesicle, AP: 0.207
Precision
Recall
0.0
0.2
0.4
0.6
0.8
1.0

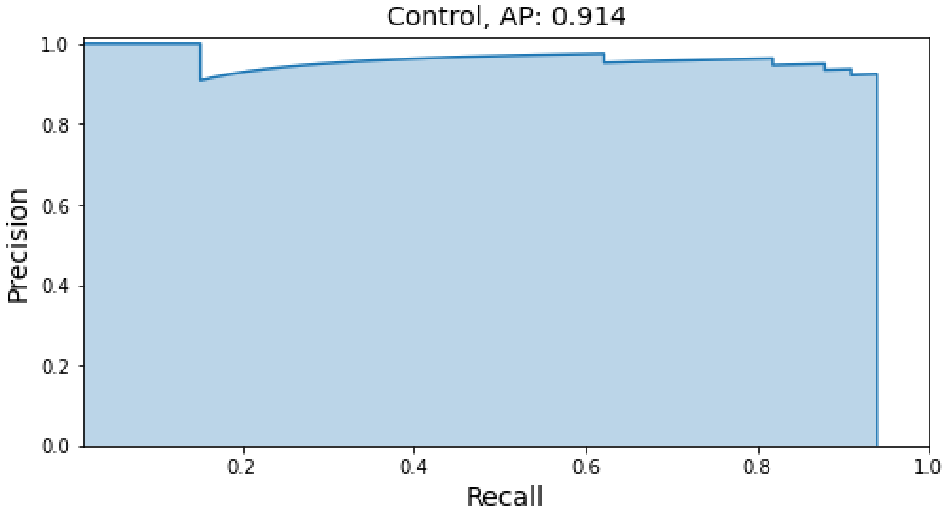
Control, AP: 0.914
Precision
Recall
0.0
0.2
0.4
0.6
0.8
1.0

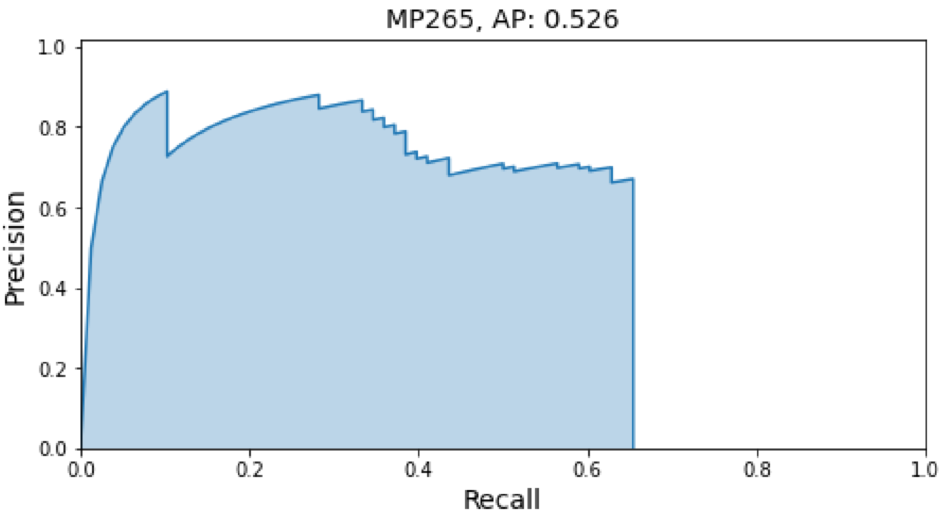

Quality Control Metrics

class	false positive	true positive	false negative	recall	precision	accuracy	f1 score	average_precision
Rifampicin	8	103	9	0.92	0.928	0.92	0.924	0.879
Oblique	27	81	12	0.871	0.75	0.871	0.806	0.769
Nalidixate	7	41	17	0.707	0.854	0.707	0.774	0.641
Mecillinam	22	64	27	0.703	0.744	0.703	0.723	0.605
CAM	27	44	1	0.978	0.62	0.978	0.759	0.754
Vesicle	13	19	48	0.284	0.594	0.284	0.384	0.207
Control	6	62	4	0.939	0.912	0.939	0.925	0.914
MP265	25	51	27	0.654	0.671	0.654	0.662	0.526

Mean average precision (mAP) over the all classes is: 0.662

Example Quality Control Visualisation

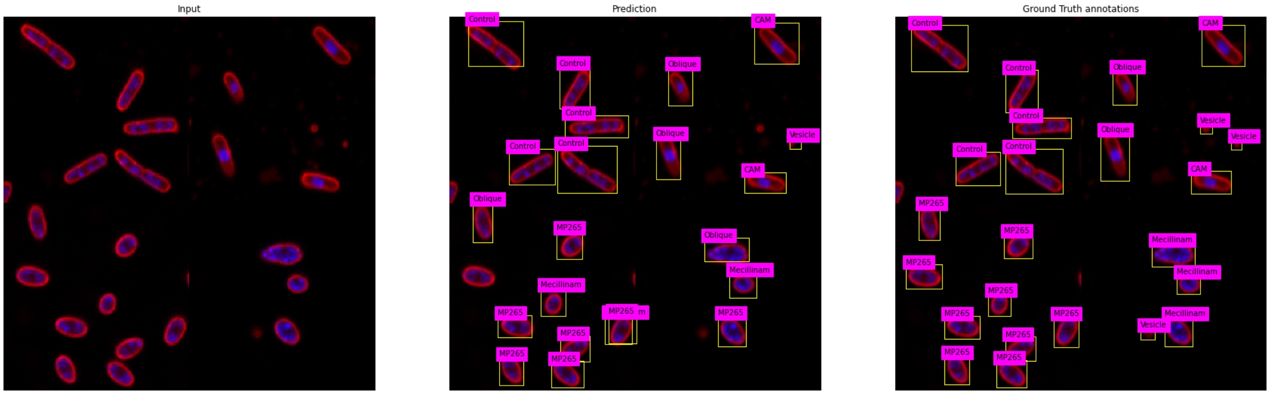

References:

- ZeroCostDL4Mic: von Chamier, Lucas & Laine, Romain, et al. "ZeroCostDL4Mic: an open platform to simplify access and use of Deep-Learning in Microscopy." bioRxiv (2020).
- YOLOv2: Redmon, Joseph, and Ali Farhadi. "YOLO9000: better, faster, stronger." Proceedings of the IEEE conference on computer vision and pattern recognition. 2017.
- YOLOv2 keras: https://github.com/experiencor/keras-yolo2, (2018)

To find the parameters and other information about how this model was trained, go to the training_report.pdf of this model which should be in the folder of the same name.
