# Supplementary material for: DeepBacs for multi-task bacterial image analysis using open-source deep learning approaches
Source: Commun Biol. 2022 Jul 9;5:688. doi: 10.1038/s42003-022-03634-z (PMC9271087; doi:10.1038/s42003-022-03634-z)
Supplement: Supplementary file 15 — Supplementary Data 1 [file 42003_2022_3634_MOESM15_ESM.zip › Figure_3/Antibiotic_phenotyping/YOLOv2_Model_reports/All_antibiotic/M1_training_report.pdf]

Training report for YOLOv2 model (M2_new_DNA_membr_100ep_batch_16_train_4_aug_8_LR_0.001_val_20_FPP2_FNP4_FCP_2_full_YOLO):

Date: 2021-02-17

Training time: 2.0hour(s) 32.0min(s) 42sec(s)

Information for your materials and methods:

The YOLOv2 model was trained from scratch for 97 epochs on 153 labelled images (image dimensions: (400, 400, 3)) with a batch size of 16 and a custom loss function combining MSE and crossentropy losses, using the YOLOv2 ZeroCostDL4Mic notebook (v 1.12) (von Chamier & Laine et al., 2020). Key python packages used include tensorflow (v 0.1.12), Keras (v 2.3.1), numpy (v 1.19.5), cuda (v 10.1.243). The training was accelerated using a Tesla P100GPU.

Augmentation: The dataset was augmented by a factor of 8 by
- flipping
- rotation

Parameters

The following parameters were used for training:

Parameter	Value
number_of_epochs	97
train_times	4
batch_size	16
learning_rate	0.001
false_negative_penalty	4.0
false_positive_penalty	2.0
position_size_penalty	1.0
false_class_penalty	2.0
percentage_validation	20

Training Dataset

Training_source: /content/gdrive/MyDrive/Deep_Learning_Bacteria_EMBO/Datasets/YOLOv2/antibiotic_MoA/split_FOV/TRAINING_DATA

Training_target: /content/gdrive/MyDrive/Deep_Learning_Bacteria_EMBO/Datasets/YOLOv2/antibiotic_MoA/split_FOV/TRAINING_ANNOTATION_NEW

Model Path: /content/gdrive/MyDrive/Deep_Learning_Bacteria_EMBO/Datasets/YOLOv2/antibiotic_MoA/split_FOV/new_models_20210217/M2_new_DNA_membr_100ep_batch_16_train_4_aug_8_LR_0.001_val_20_FPP2_FNP4_FCP_2_full_YOLO

Example ground-truth annotation

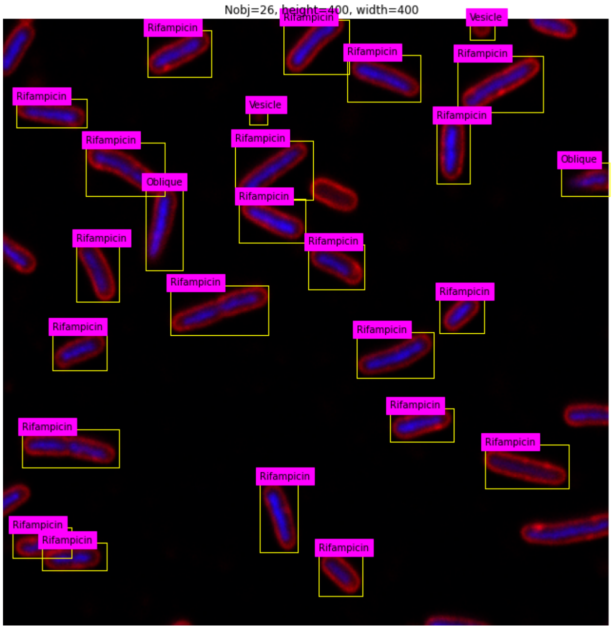

References:
- ZeroCostDL4Mic: von Chamier, Lucas & Laine, Romain, et al. "ZeroCostDL4Mic: an open platform to simplify access and use of Deep-Learning in Microscopy." bioRxiv (2020).
- YOLOv2: Redmon, Joseph, and Ali Farhadi. "YOLO9000: better, faster, stronger." Proceedings of the IEEE conference on computer vision and pattern recognition. 2017.
- YOLOv2 keras: https://github.com/experiencor/keras-yolo2, (2018)
- imgaug: Jung, Alexander et al., https://github.com/aleju/imgaug, (2020)

Important:
Remember to perform the quality control step on all newly trained models
Please consider depositing your training dataset on Zenodo
