# Supplementary material for: DeepBacs for multi-task bacterial image analysis using open-source deep learning approaches
Source: Commun Biol. 2022 Jul 9;5:688. doi: 10.1038/s42003-022-03634-z (PMC9271087; doi:10.1038/s42003-022-03634-z)
Supplement: Supplementary file 15 — Supplementary Data 1 [file 42003_2022_3634_MOESM15_ESM.zip › Figure_3/Antibiotic_phenotyping/YOLOv2_Model_reports/Without_MP265/QC_report.pdf]

Quality Control report for YOLOv2 model (without_MP265_M1_100ep_train4_batch16_val20_4_2_1_2_aug_8)
Date and Time: 2021-08-11 19:36

Development of Training Losses

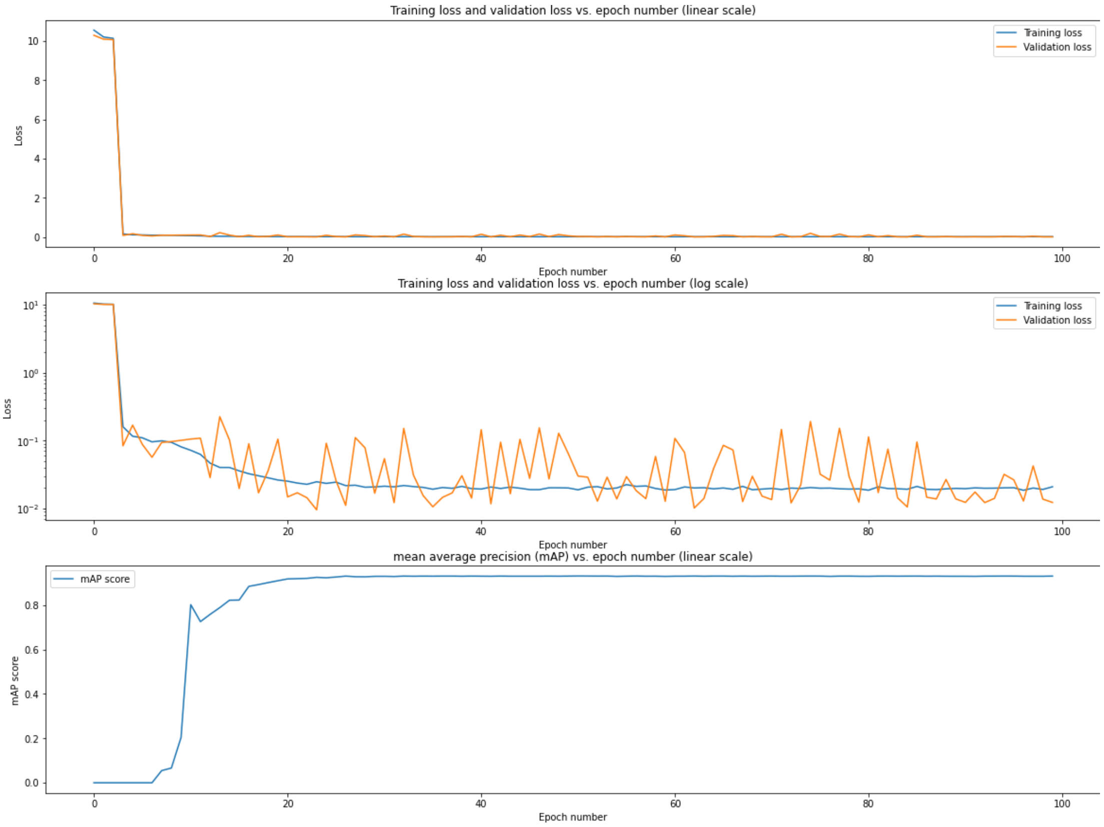

P-R curves for test dataset

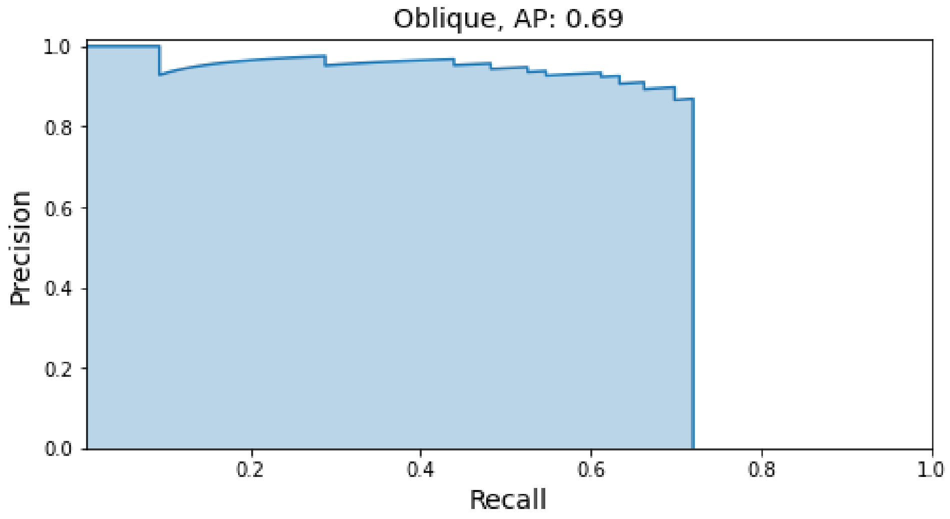

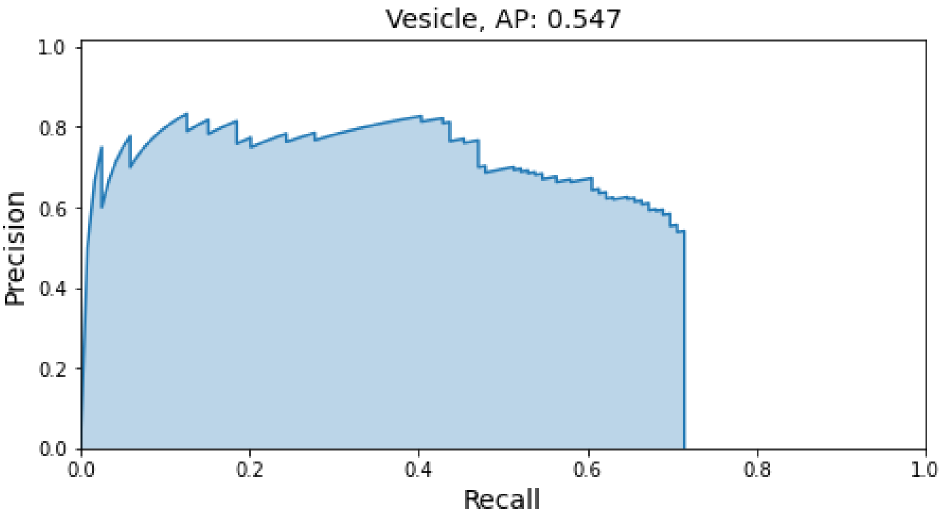
Vesicle, AP: 0.547
Precision
Recall
0.0
0.2
0.4
0.6
0.8
1.0

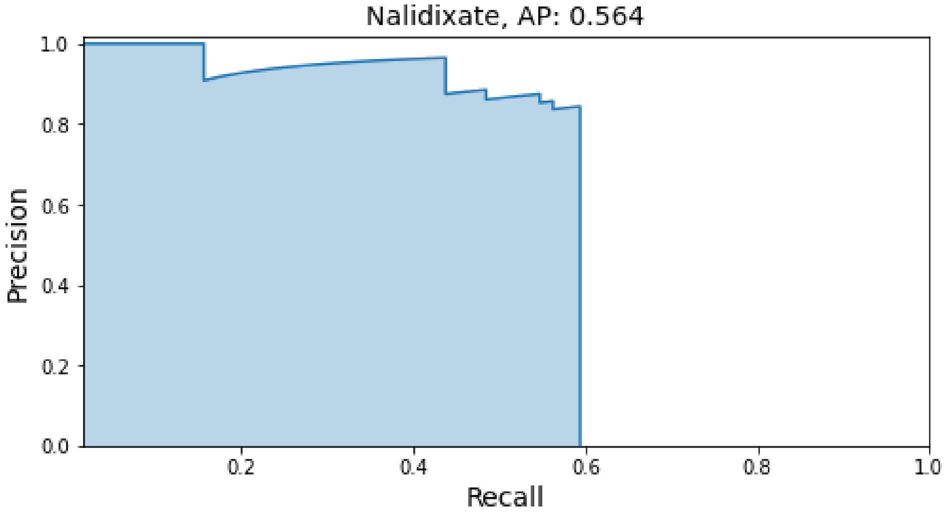
Nalidixate, AP: 0.564
Precision
Recall
0.0
0.2
0.4
0.6
0.8
1.0

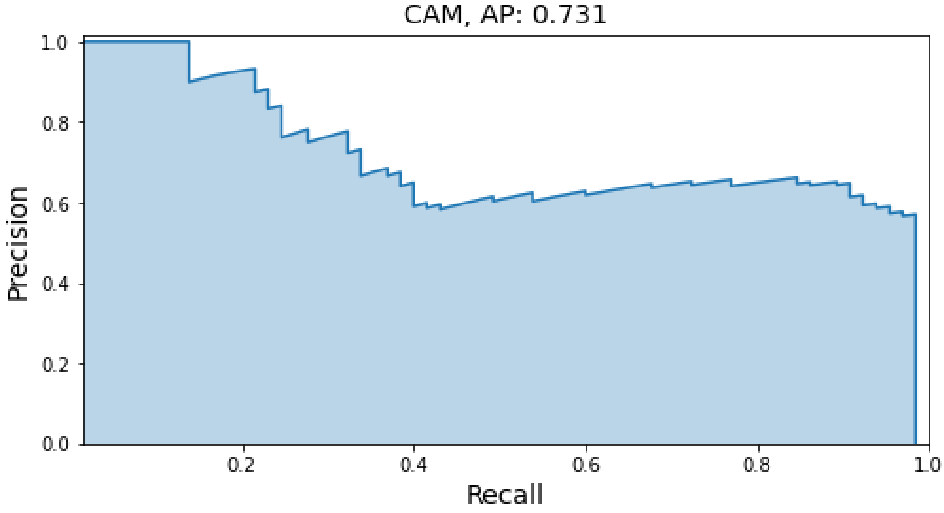
CAM, AP: 0.731
Precision
Recall
0.0
0.2
0.4
0.6
0.8
1.0

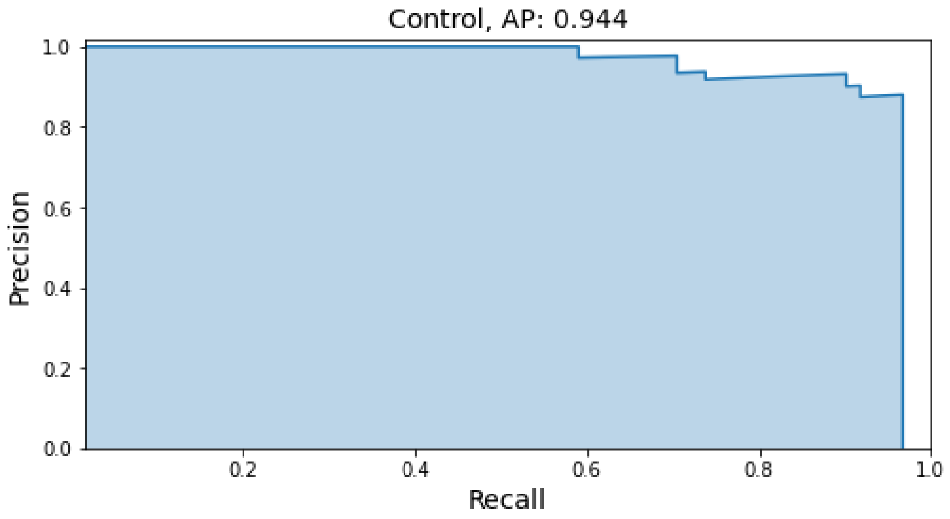

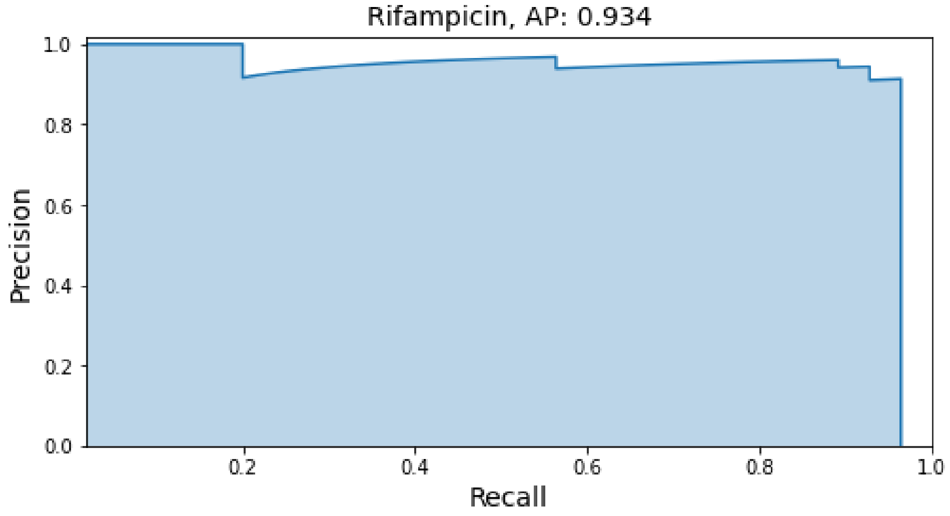

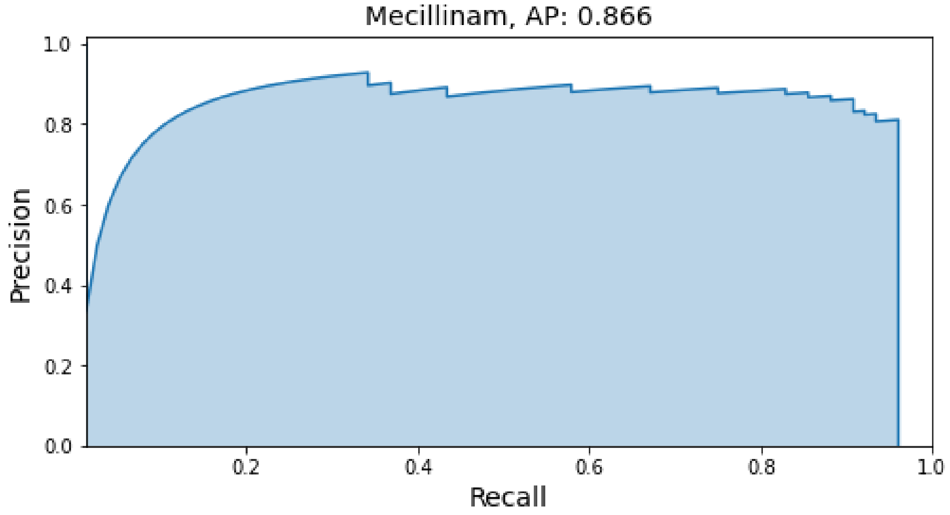

Quality Control Metrics

class	false positive	true positive	false negative	recall	precision	accuracy	f1 score	average_precision
Oblique	15	100	39	0.719	0.87	0.719	0.787	0.69
Vesicle	78	85	34	0.714	0.521	0.714	0.603	0.547
Nalidixate	7	38	26	0.594	0.844	0.594	0.697	0.564
CAM	51	64	1	0.985	0.557	0.985	0.711	0.731
Control	12	59	2	0.967	0.831	0.967	0.894	0.944
Rifampicin	9	53	2	0.964	0.855	0.964	0.906	0.934
Mecillinam	17	73	3	0.961	0.811	0.961	0.88	0.866

Mean average precision (mAP) over the all classes is: 0.754

Example Quality Control Visualisation

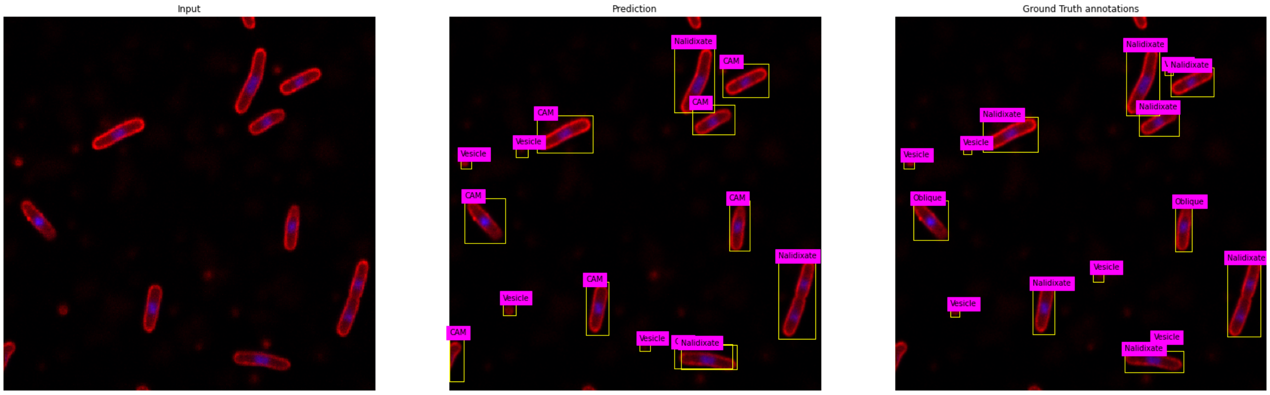

References:
- ZeroCostDL4Mic: von Chamier, Lucas & Laine, Romain, et al. "Democratising deep learning for microscopy with ZeroCostDL4Mic." Nature Communications (2021).
- YOLOv2: Redmon, Joseph, and Ali Farhadi. "YOLO9000: better, faster, stronger." Proceedings of the IEEE conference on computer vision and pattern recognition. 2017.
- YOLOv2 keras: https://github.com/experiencor/keras-yolo2, (2018)

To find the parameters and other information about how this model was trained, go to the training_report.pdf of this model which should be in the folder of the same name.
