# Supplementary material for: DeepBacs for multi-task bacterial image analysis using open-source deep learning approaches
Source: Commun Biol. 2022 Jul 9;5:688. doi: 10.1038/s42003-022-03634-z (PMC9271087; doi:10.1038/s42003-022-03634-z)
Supplement: Supplementary file 15 — Supplementary Data 1 [file 42003_2022_3634_MOESM15_ESM.zip › Figure_3/Antibiotic_phenotyping/YOLOv2_Model_reports/Without_MP265/Training_Report.pdf]

Training report for YOLOv2 model (without_MP265_M1_100ep_train4_batch16_val20_4_2_1_2_aug_8):
Date: 2021-08-10
Training time: 4.0hour(s) 31.0min(s) 35sec(s)

Information for your materials and methods:

The YOLOv2 model was trained from scratch for 97 epochs on 137 labelled images (image dimensions: (400, 400, 3)) with a batch size of 8 and a custom loss function combining MSE and crossentropy losses, using the YOLOv2 ZeroCostDL4Mic notebook (v 1) (von Chamier & Laine et al., 2020). Key python packages used include tensorflow (v 0.1.12), Keras (v 2.3.1), numpy (v 1.19.5), cuda (v 11.0.221
Build cuda_11.0_bu.TC445_37.28845127_0). The training was accelerated using a Tesla T4 GPU.

Augmentation: The dataset was augmented by a factor of 8 by
- flipping
- rotation

Parameters

Default Advanced Parameters were enabled

Parameter	Value
number_of_epochs	97
train_times	4
batch_size	8
learning_rate	0.0001
false_negative_penalty	5.0
false_positive_penalty	1.0
position_size_penalty	1.0
false_class_penalty	1.0
percentage_validation	10

Training Dataset

Training_source: /content/gdrive/MyDrive/Deep_Learning_Bacteria_EMBO/20210507_NEW_YOLO_ANNOTATIONS_prelim_folder/Antibiotic_profiling_leave_one_out/without_MP265/train_images

Training_target: /content/gdrive/MyDrive/Deep_Learning_Bacteria_EMBO/20210507_NEW_YOLO_ANNOTATIONS_prelim_folder/Antibiotic_profiling_leave_one_out/without_MP265/train_annotations

Model Path: /content/gdrive/MyDrive/Deep_Learning_Bacteria_EMBO/20210507_NEW_YOLO_ANNOTATIONS_prelim_folder/Antibiotic_profiling_leave_one_out/without_MP265/models/without_MP265_M1_100ep_train4_batch16_val20_4_2_1_2_aug_8

Example ground-truth annotation

Nobj=22, height=400, width=400

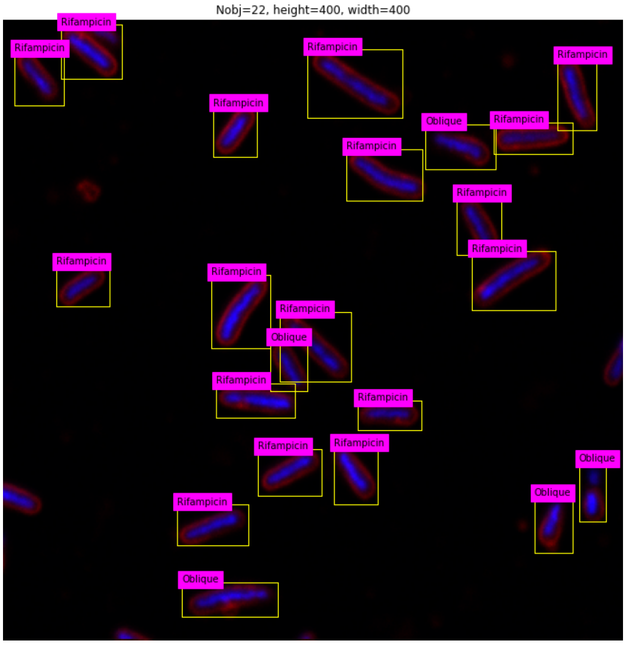

References:
- ZeroCostDL4Mic: von Chamier, Lucas & Laine, Romain, et al. "Democratising deep learning for microscopy with ZeroCostDL4Mic." Nature Communications (2021).
- YOLOv2: Redmon, Joseph, and Ali Farhadi. "YOLO9000: better, faster, stronger." Proceedings of the IEEE conference on computer vision and pattern recognition. 2017.
- YOLOv2 keras: https://github.com/experiencor/keras-yolo2, (2018)
- imgaug: Jung, Alexander et al., https://github.com/aleju/imgaug, (2020)

Important:
Remember to perform the quality control step on all newly trained models
Please consider depositing your training dataset on Zenodo
