# Supplementary material for: DeepBacs for multi-task bacterial image analysis using open-source deep learning approaches
Source: Commun Biol. 2022 Jul 9;5:688. doi: 10.1038/s42003-022-03634-z (PMC9271087; doi:10.1038/s42003-022-03634-z)
Supplement: Supplementary file 15 — Supplementary Data 1 [file 42003_2022_3634_MOESM15_ESM.zip › Figure_3/Growth_stage_analysis/YOLOv2_Model_reports/Large_FoV/QC_report.pdf]

Quality Control report for YOLOv2 model (Full_FOV_M2_100ep_100box_4xaug_FNP5_FPP_1_PSP_3_FCP3_train4_batch_4_LR_1E-4_val20)

Date and Time: 2021-05-14 06:47

Development of Training Losses

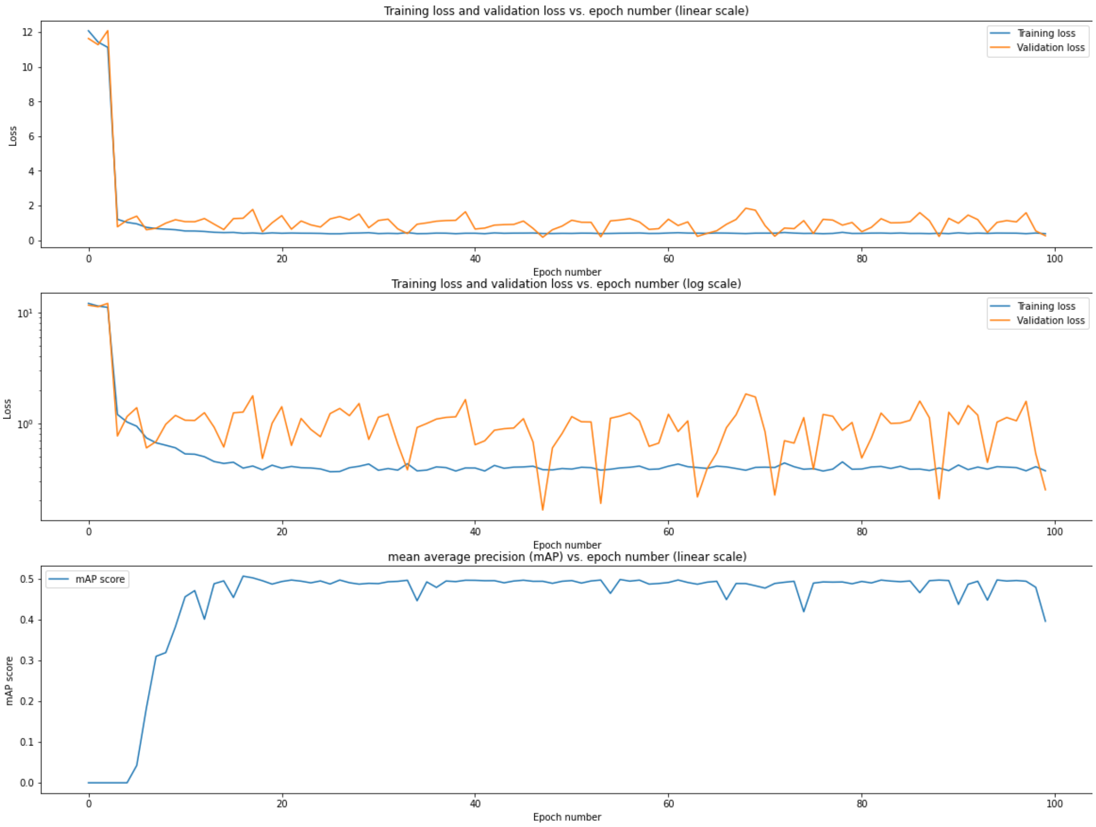

P-R curves for test dataset

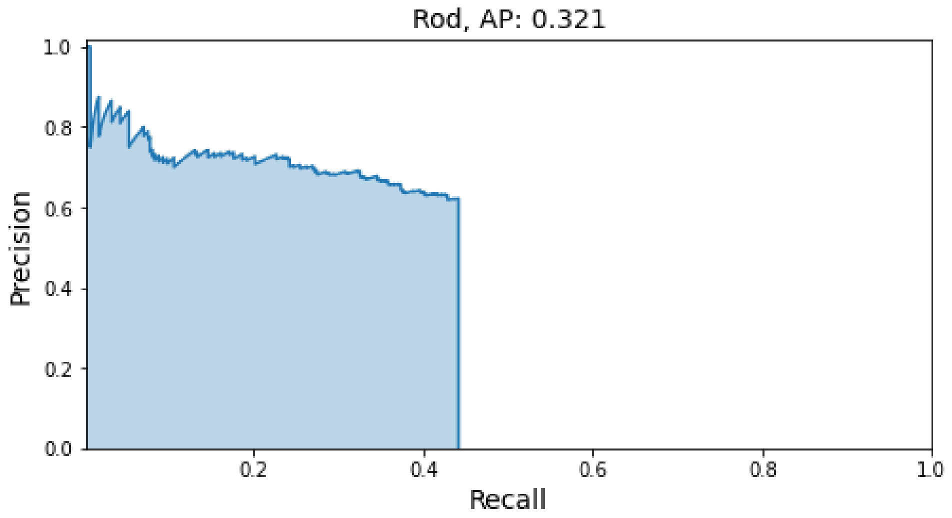

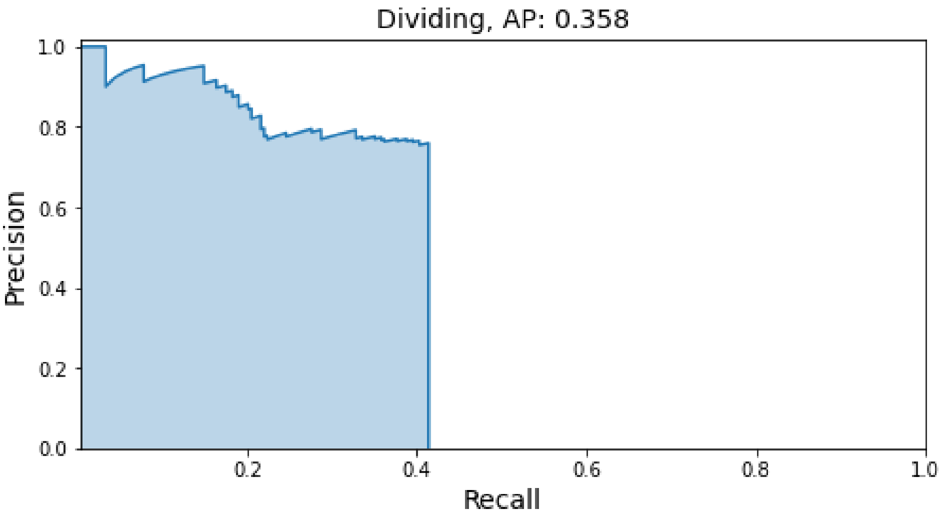

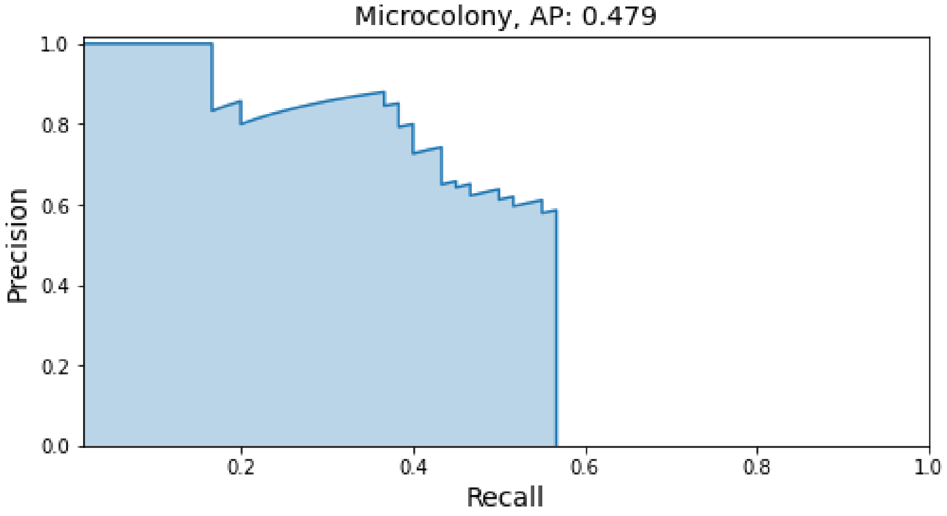

Quality Control Metrics

class	false positive	true positive	false negative	recall	precision	accuracy	f1 score	average_precision
Rod	107	175	221	0.442	0.621	0.442	0.516	0.321
Dividing	35	111	157	0.414	0.76	0.414	0.536	0.358
Microcolony	26	34	26	0.567	0.567	0.567	0.567	0.479

Mean average precision (mAP) over the all classes is: 0.386

Example Quality Control Visualisation

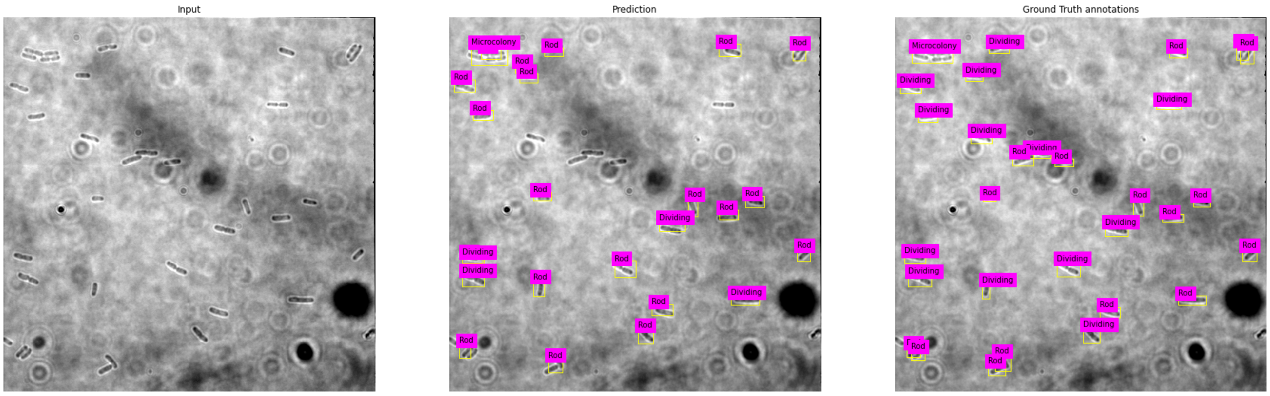

References:

- ZeroCostDL4Mic: von Chamier, Lucas & Laine, Romain, et al. "Democratising deep learning for microscopy with ZeroCostDL4Mic." Nature Communications (2021).
- YOLOv2: Redmon, Joseph, and Ali Farhadi. "YOLO9000: better, faster, stronger." Proceedings of the IEEE conference

on computer vision and pattern recognition. 2017.
- YOLOv2 keras: https://github.com/experiencor/keras-yolo2, (2018)

To find the parameters and other information about how this model was trained, go to the training_report.pdf of this model which should be in the folder of the same name.
