# Supplementary material for: DeepBacs for multi-task bacterial image analysis using open-source deep learning approaches
Source: Commun Biol. 2022 Jul 9;5:688. doi: 10.1038/s42003-022-03634-z (PMC9271087; doi:10.1038/s42003-022-03634-z)
Supplement: Supplementary file 15 — Supplementary Data 1 [file 42003_2022_3634_MOESM15_ESM.zip › Figure_3/Growth_stage_analysis/YOLOv2_Model_reports/Small_FoV/QC_report.pdf]

Quality Control report for YOLOv2 model (Quartered_M3_100ep_30box_4xaug_FNP5_FPP_1_PSP_3_FCP3_train4_batch_8_LR_1E-4_val20)

Date and Time: 2021-05-13 22:51

Development of Training Losses

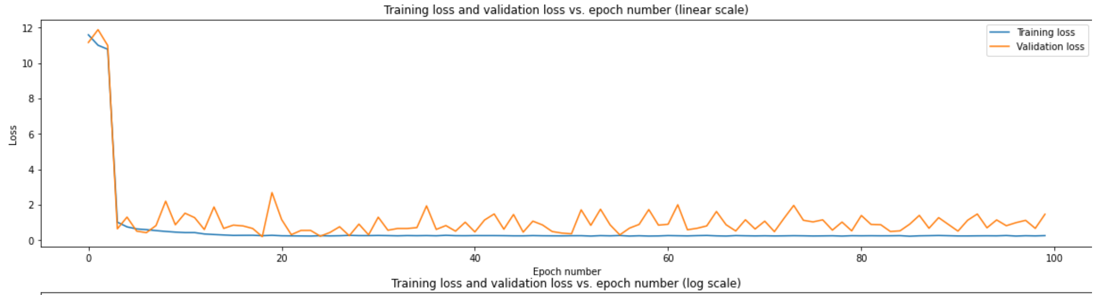

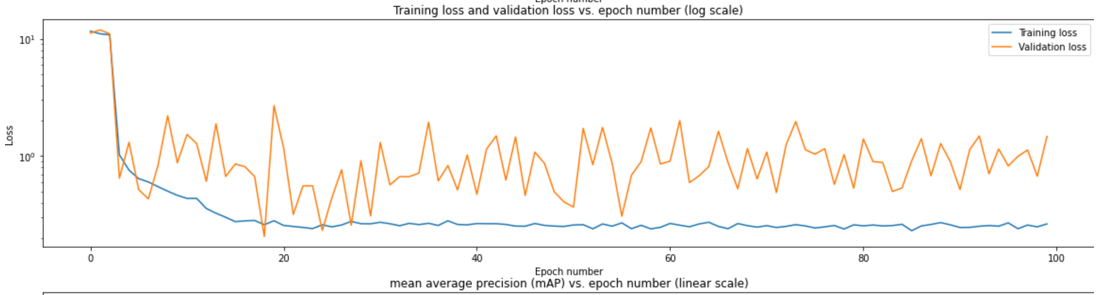

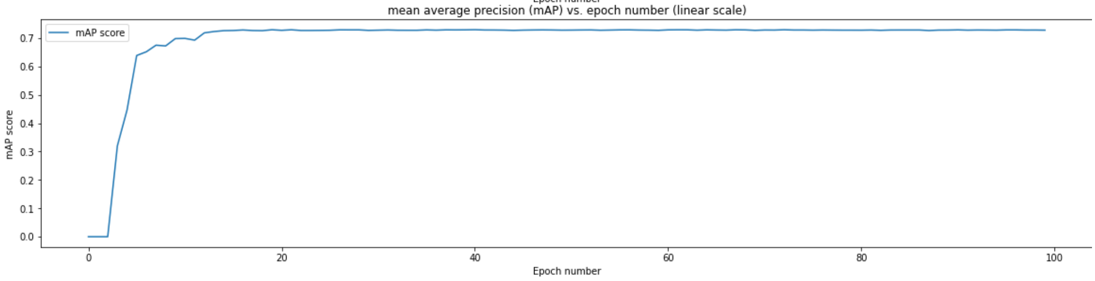

P-R curves for test dataset

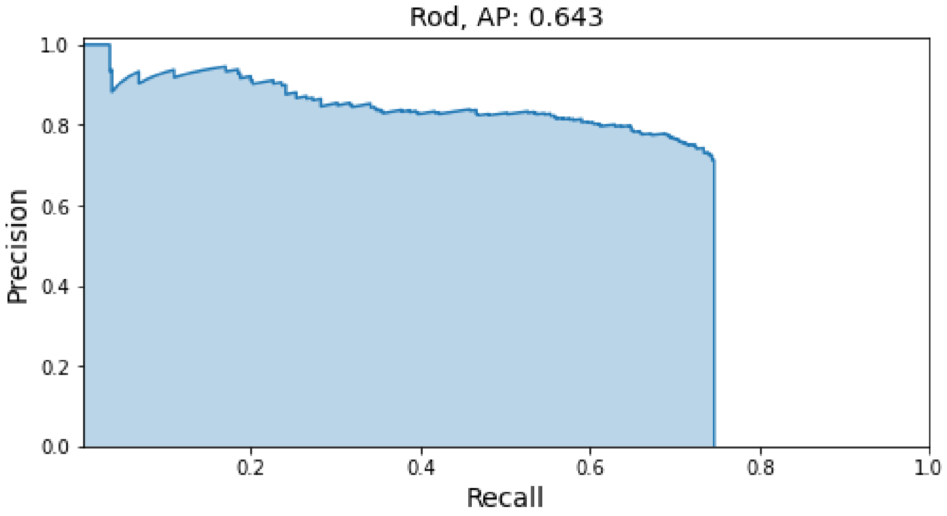

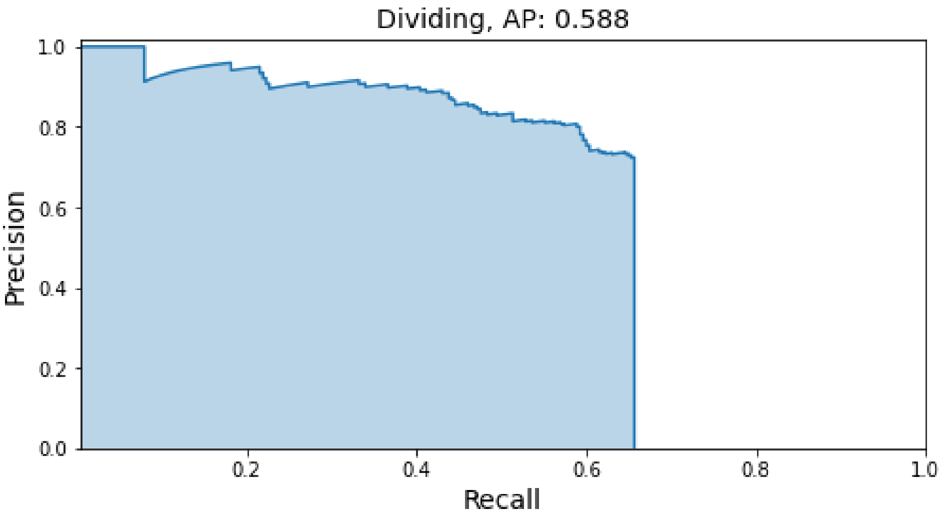

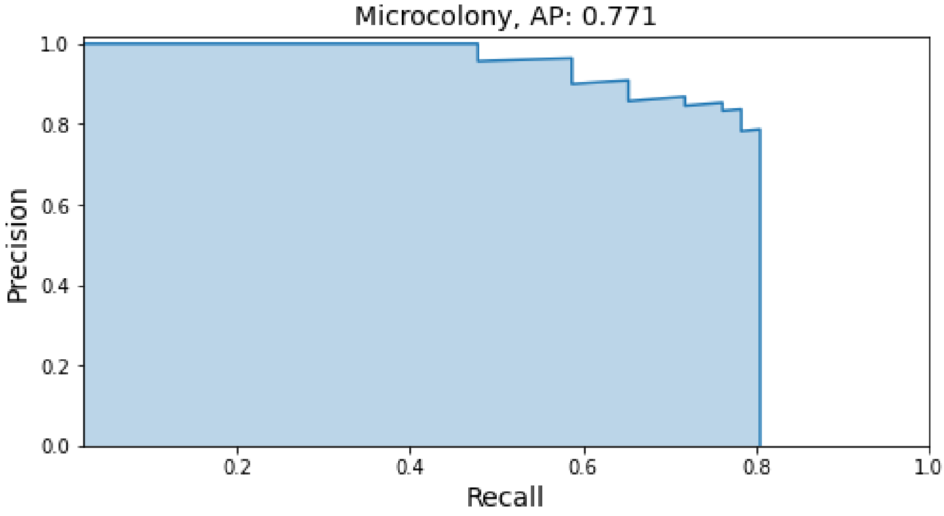

Quality Control Metrics

class	false positive	true positive	false negative	recall	precision	accuracy	f1 score	average_precision
Rod	124	306	104	0.746	0.712	0.746	0.729	0.643
Dividing	67	174	91	0.657	0.722	0.657	0.688	0.588
Microcolony	12	37	9	0.804	0.755	0.804	0.779	0.771

Mean average precision (mAP) over the all classes is: 0.667

Example Quality Control Visualisation

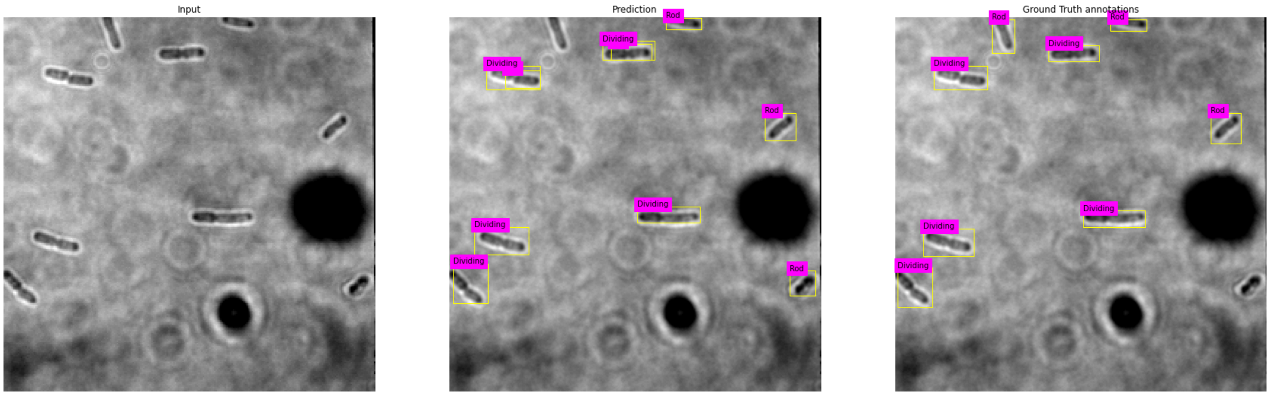

References:
- ZeroCostDL4Mic: von Chamier, Lucas & Laine, Romain, et al. "Democratising deep learning for microscopy with ZeroCostDL4Mic." Nature Communications (2021).
- YOLOv2: Redmon, Joseph, and Ali Farhadi. "YOLO9000: better, faster, stronger." Proceedings of the IEEE conference

on computer vision and pattern recognition. 2017.
- YOLOv2 keras: https://github.com/experiencor/keras-yolo2, (2018)

To find the parameters and other information about how this model was trained, go to the training_report.pdf of this model which should be in the folder of the same name.
