# Supplementary material for: DeepBacs for multi-task bacterial image analysis using open-source deep learning approaches
Source: Commun Biol. 2022 Jul 9;5:688. doi: 10.1038/s42003-022-03634-z (PMC9271087; doi:10.1038/s42003-022-03634-z)
Supplement: Supplementary file 15 — Supplementary Data 1 [file 42003_2022_3634_MOESM15_ESM.zip › Figure_4/Figure_4fg_DeepBugs_MreB_tracking_analysis/mreb_denoise_final_figures.html]

Untitled 

%REQUIRES https://github.com/bastibe/Violinplot-Matlab

load('MreB\_denoise\_trackmate\_results\_all\_conditions.mat');

violin plot of the track length

cats = [repmat("Training",size(MreBhighSNR,1),1);...

repmat("Raw",size(fast1raw,1),1);...

repmat("PureDenoise",size(fast1PureDenoise,1),1);...

repmat("CARE",size(fast1CARE,1),1)];

trackDuration = [MreBhighSNR.TRACK\_DURATION;fast1raw.TRACK\_DURATION;...

fast1PureDenoise.TRACK\_DURATION;fast1CARE.TRACK\_DURATION] ;

h1 = figure

h1 =

Figure (1) with properties:
Number: 1
Name: ''
Color: [0.9400 0.9400 0.9400]
Position: [1000 918 560 420]
Units: 'pixels'
Show all properties

violinplot(trackDuration,cats,'GroupOrder',{'Training','Raw','PureDenoise','CARE'},'ShowData',false);

ylabel('Track duration (s)');

hA=gca;

hA.TickDir='out';

hA.LineWidth=1;

hA.FontName='Arial';

h1.Color='w'

h1 =

Figure (1) with properties:
Number: 1
Name: ''
Color: [1 1 1]
Position: [1000 918 560 420]
Units: 'pixels'
Show all properties

h1.Visible='on';

saveas(h1,'MreB\_track\_length.pdf')

saveas(h1,'MreB\_track\_length.fig')

trackSpeedNm = 1000\*[MreBhighSNR.TRACK\_MEAN\_SPEED;fast1raw.TRACK\_MEAN\_SPEED;...

fast1PureDenoise.TRACK\_MEAN\_SPEED;fast1CARE.TRACK\_MEAN\_SPEED] ;

h1=figure;

violinplot(trackSpeedNm,cats,'GroupOrder',{'Training','Raw','PureDenoise','CARE'},'ShowData',false);

ylabel('Speed (nm/ s)');

ylim([0 150]);

hA=gca;

hA.TickDir='out';

hA.LineWidth=1;

hA.FontName='Arial';

h1.Color='w'

h1 =

Figure (2) with properties:
Number: 2
Name: ''
Color: [1 1 1]
Position: [1000 918 560 420]
Units: 'pixels'
Show all properties

h1.Visible='on';

saveas(h1,'MreB\_speedMax150.pdf')

saveas(h1,'MreB\_speedMax150.fig')

  
