# Supplementary material for: DeepBacs for multi-task bacterial image analysis using open-source deep learning approaches
Source: Commun Biol. 2022 Jul 9;5:688. doi: 10.1038/s42003-022-03634-z (PMC9271087; doi:10.1038/s42003-022-03634-z)
Supplement: Supplementary file 15 — Supplementary Data 1 [file 42003_2022_3634_MOESM15_ESM.zip › Figure_S3/StarDist_model_Quality_control/QC_report.pdf]

Quality Control report for Stardist 2D model (Mixed_M1_StarDist_256px_200ep_batch_4_120_steps_64_rays_grid_2_10val_aug3)

Date: 2021-04-06

Development of Training Losses

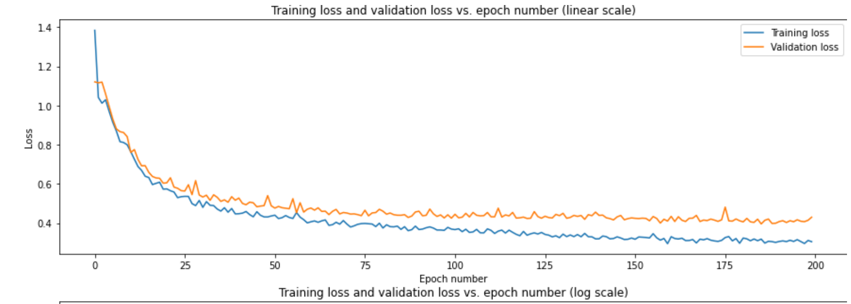

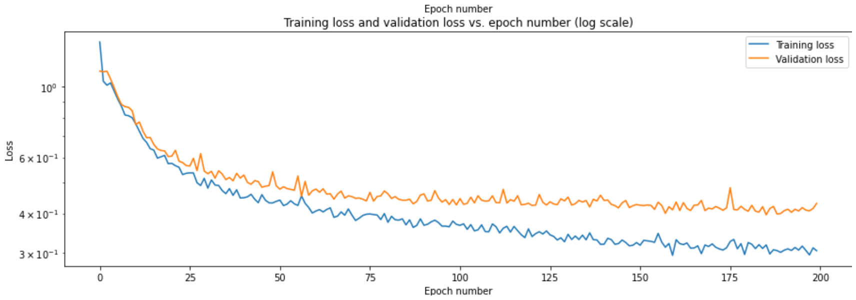

Example Quality Control Visualisation

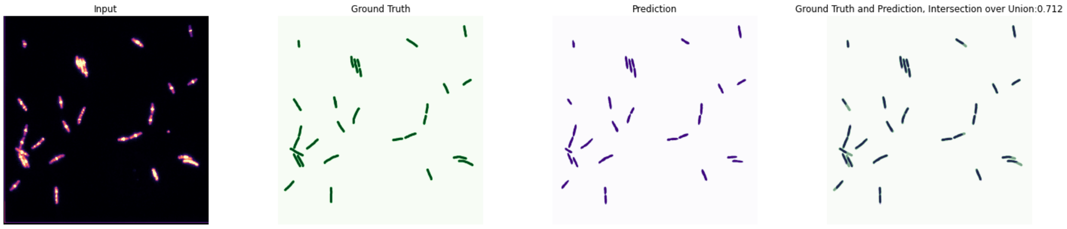

Quality Control Metrics

image #	Prediction v. GT IoU	false pos.	true pos.	false neg.	precision	recall	accuracy	f1 score	n_true	n_pred	mean_true_score	mean_matched_score	panoptic_quality
1	0.712	5	24	9	0.828	0.727	0.632	0.774	33	29	0.532	0.732	0.566
2	0.678	12	44	24	0.786	0.647	0.55	0.71	68	56	0.461	0.713	0.506
3	0.665	25	70	39	0.737	0.642	0.522	0.686	109	95	0.453	0.706	0.484
4	0.757	3	24	7	0.889	0.774	0.706	0.828	31	27	0.587	0.758	0.627
5	0.609	7	33	10	0.825	0.767	0.66	0.795	43	40	0.575	0.749	0.596
6	0.725	4	26	6	0.867	0.812	0.722	0.839	32	30	0.611	0.752	0.631
7	0.692	16	66	29	0.805	0.695	0.595	0.746	95	82	0.496	0.714	0.533
8	0.685	4	38	10	0.905	0.792	0.731	0.844	48	42	0.593	0.75	0.633
9	0.707	3	48	8	0.941	0.857	0.814	0.897	56	51	0.65	0.758	0.68
10	0.778	4	52	8	0.929	0.867	0.812	0.897	60	56	0.675	0.779	0.698
11	0.759	3	69	3	0.958	0.958	0.92	0.958	72	72	0.739	0.771	0.739
12	0.744	9	73	15	0.89	0.83	0.753	0.859	88	82	0.618	0.745	0.64
13	0.753	9	96	14	0.914	0.873	0.807	0.893	110	105	0.659	0.756	0.675
14	0.722	26	92	42	0.78	0.687	0.575	0.73	134	118	0.485	0.707	0.516
15	0.735	2	34	5	0.944	0.872	0.829	0.907	39	36	0.664	0.761	0.69
16	0.769	1	47	1	0.979	0.979	0.959	0.979	48	48	0.774	0.79	0.774
17	0.758	6	45	12	0.882	0.789	0.714	0.833	57	51	0.61	0.773	0.644
18	0.678	8	55	10	0.873	0.846	0.753	0.859	65	63	0.618	0.731	0.628
19	0.724	18	55	37	0.753	0.598	0.5	0.667	92	73	0.422	0.705	0.47
20	0.77	2	57	0	0.966	1.0	0.966	0.983	57	59	0.782	0.782	0.769

21	0.72	9	59	13	0.868	0.819	0.728	0.843	72	68	0.617	0.753	0.635
22	0.734	13	71	17	0.845	0.807	0.703	0.826	88	84	0.621	0.77	0.636
23	0.738	17	86	30	0.835	0.741	0.647	0.785	116	103	0.544	0.734	0.577
24	0.729	30	94	58	0.758	0.618	0.516	0.681	152	124	0.446	0.721	0.491
25	0.672	65	132	95	0.67	0.581	0.452	0.623	227	197	0.387	0.666	0.414
26	0.685	15	74	2	0.831	0.974	0.813	0.897	76	89	0.751	0.771	0.692
27	0.705	15	128	0	0.895	1.0	0.895	0.945	128	143	0.755	0.755	0.713
28	0.737	4	71	0	0.947	1.0	0.947	0.973	71	75	0.76	0.76	0.739
29	0.715	10	72	0	0.878	1.0	0.878	0.935	72	82	0.771	0.771	0.721
30	0.725	8	73	3	0.901	0.961	0.869	0.93	76	81	0.738	0.769	0.715
31	0.841	5	84	3	0.944	0.966	0.913	0.955	87	89	0.843	0.873	0.833
32	0.86	6	137	0	0.958	1.0	0.958	0.979	137	143	0.882	0.882	0.863
33	0.864	1	74	0	0.987	1.0	0.987	0.993	74	75	0.87	0.87	0.864
34	0.85	5	77	0	0.939	1.0	0.939	0.969	77	82	0.875	0.875	0.847
35	0.863	6	75	2	0.926	0.974	0.904	0.949	77	81	0.875	0.898	0.853

References:
- ZeroCostDL4Mic: von Chamier, Lucas & Laine, Romain, et al. "ZeroCostDL4Mic: an open platform to simplify access and use of Deep-Learning in Microscopy." BioRxiv (2020).
- StarDist 2D: Schmidt, Uwe, et al. "Cell detection with star-convex polygons." International Conference on Medical Image Computing and Computer-Assisted Intervention. Springer, Cham, 2018.

To find the parameters and other information about how this model was trained, go to the training_report.pdf of this model which should be in the folder of the same name.
