# Supplementary material for: DeepBacs for multi-task bacterial image analysis using open-source deep learning approaches
Source: Commun Biol. 2022 Jul 9;5:688. doi: 10.1038/s42003-022-03634-z (PMC9271087; doi:10.1038/s42003-022-03634-z)
Supplement: Supplementary file 15 — Supplementary Data 1 [file 42003_2022_3634_MOESM15_ESM.zip › Figure_S4/Model_validation/Training_report.pdf]

Training report for StarDist 2D model (StarDist_200_ep_30_steps_512px_batch_4_60_rays_grid2_20_val_aug4)
Date: 2021-08-01
Training time: 0.0hour(s) 23.0min(s) 58sec(s)

Information for your materials and method:

The StarDist 2D model was trained from scratch for 200 epochs on 36 paired image patches (image dimensions: (512, 512), patch size: (512,512)) with a batch size of 4 and a mae loss function, using the StarDist 2D ZeroCostDL4Mic notebook (v 1) (von Chamier & Laine et al., 2020). Key python packages used include tensorflow (v 0.1.12), Keras (v 2.3.1), csbdeep (v 0.6.2), numpy (v 1.19.5), cuda (v 11.0.221
Build cuda_11.0_bu.TC445_37.28845127_0). The training was accelerated using a Tesla T4 GPU.

Augmentation: The dataset was augmented by a factor of 4

Parameters

The following parameters were used for training:

Parameter	Value
number_of_epochs	200
patch_size	512x512
batch_size	4
number_of_steps	30
percentage_validation	20
n_rays	60
grid_parameter	2
initial_learning_rate	0.0003

Training Dataset

Training_source: /content/gdrive/MyDrive/Deep_Learning_Bacteria_EMBO/Datasets/Segmentation_Alex_Bisson/Data/Brightfield

Training_target: /content/gdrive/MyDrive/Deep_Learning_Bacteria_EMBO/Datasets/Segmentation_Alex_Bisson/Data/Masks

Model Path: /content/gdrive/MyDrive/Deep_Learning_Bacteria_EMBO/Datasets/Segmentation_Alex_Bisson/Models/StarDist_200_ep_30_steps_512px_batch_4_60_rays_grid2_20_val_aug4

Example Training pair

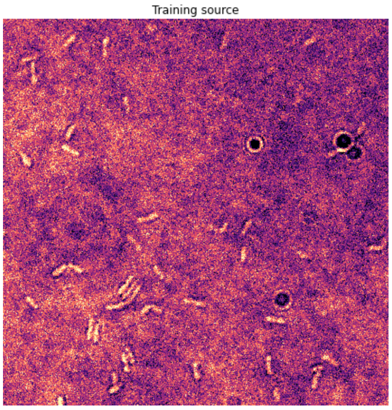

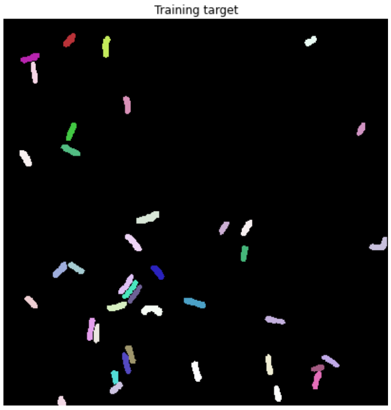

References:

- ZeroCostDL4Mic: von Chamier, Lucas & Laine, Romain, et al. "Democratising deep learning for microscopy with ZeroCostDL4Mic." Nature Communications (2021).
- StarDist 2D: Schmidt, Uwe, et al. "Cell detection with star-convex polygons." International Conference on Medical Image Computing and Computer-Assisted Intervention. Springer, Cham, 2018.
- Augmentor: Bloice, Marcus D., Christof Stocker, and Andreas Holzinger. "Augmentor: an image augmentation library for machine learning." arXiv preprint arXiv:1708.04680 (2017).

Important:

Remember to perform the quality control step on all newly trained models
Please consider depositing your training dataset on Zenodo
