# Supplementary figures and images for: DeepBacs for multi-task bacterial image analysis using open-source deep learning approaches
Source: Commun Biol. 2022 Jul 9;5:688. doi: 10.1038/s42003-022-03634-z (PMC9271087; doi:10.1038/s42003-022-03634-z)

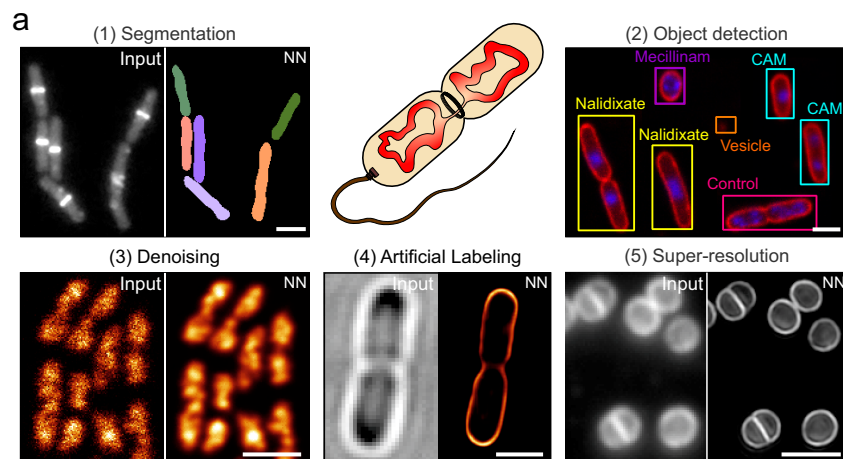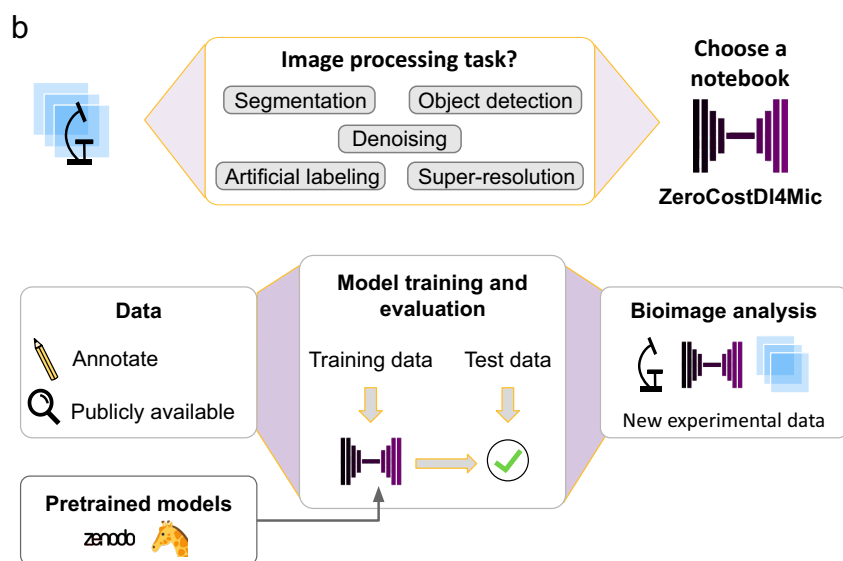

Supplement: Supplementary file 15 — Supplementary Data 1 [file 42003_2022_3634_MOESM15_ESM.zip › Figure_1/Figure_1.pdf]

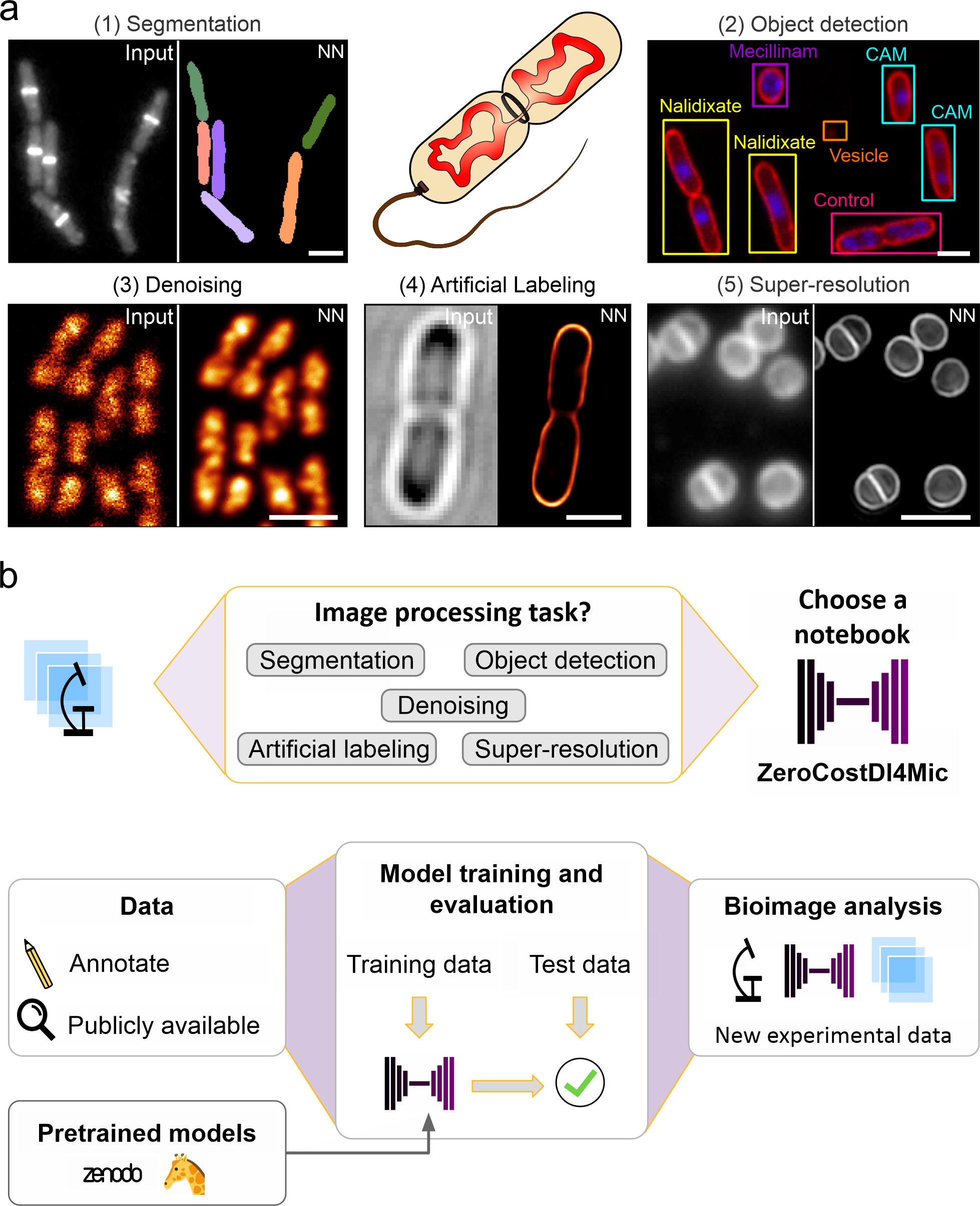

Supplement: Supplementary file 15 — Supplementary Data 1 [file 42003_2022_3634_MOESM15_ESM.zip › Figure_1/Figure_1.tif]

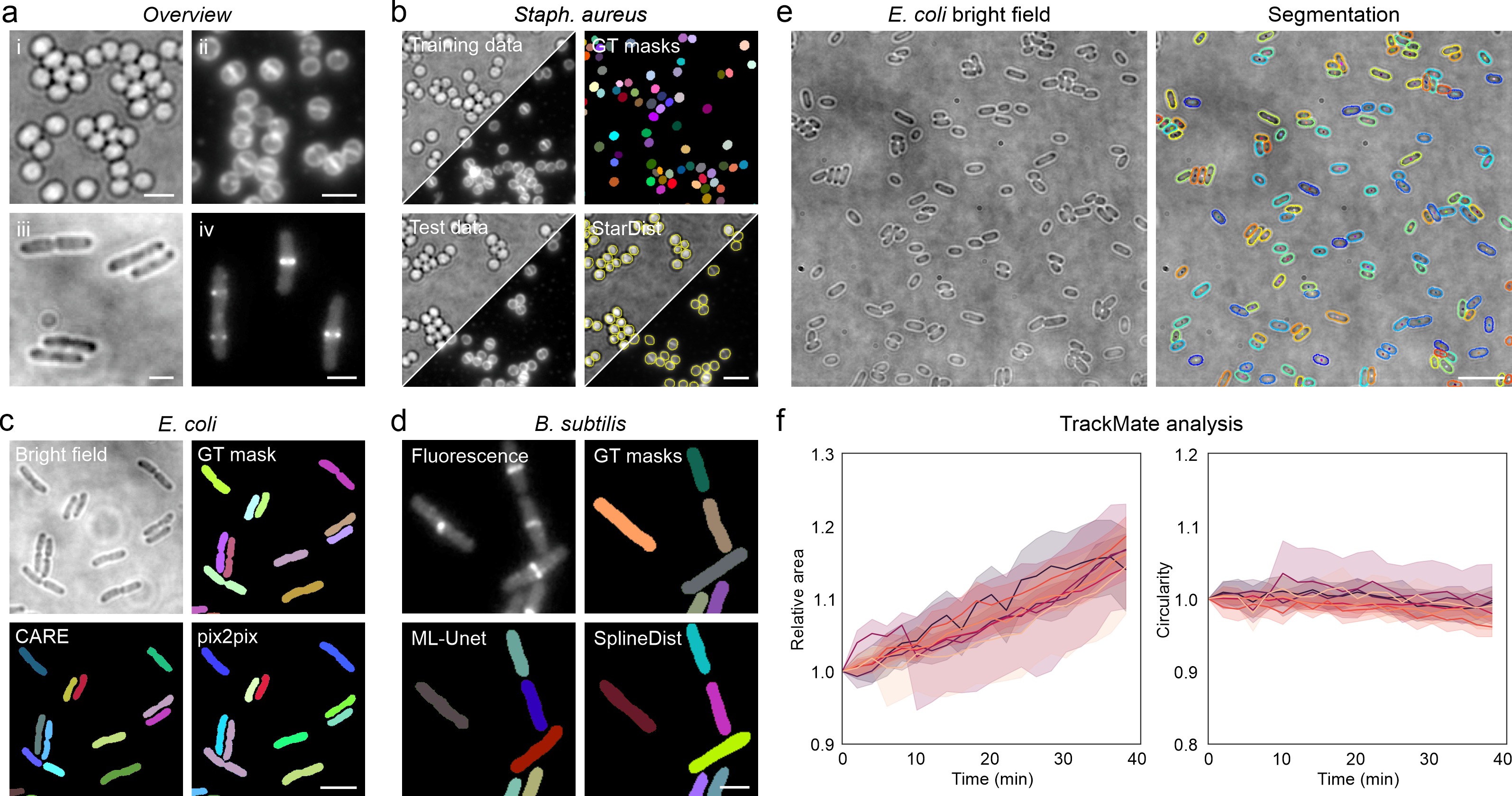

Supplement: Supplementary file 15 — Supplementary Data 1 [file 42003_2022_3634_MOESM15_ESM.zip › Figure_2/Figure_2.tif]

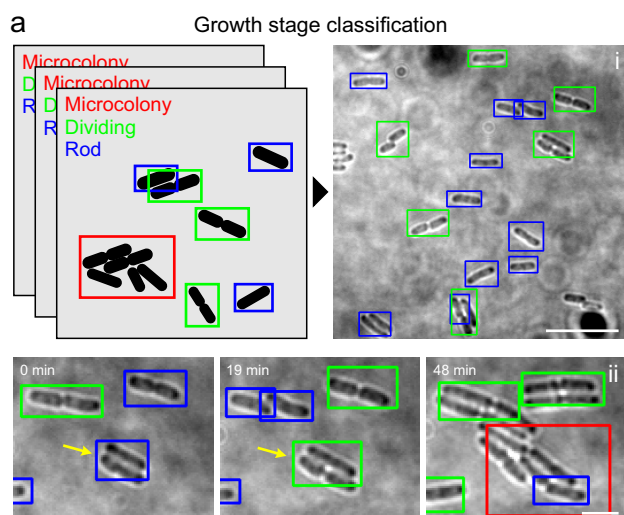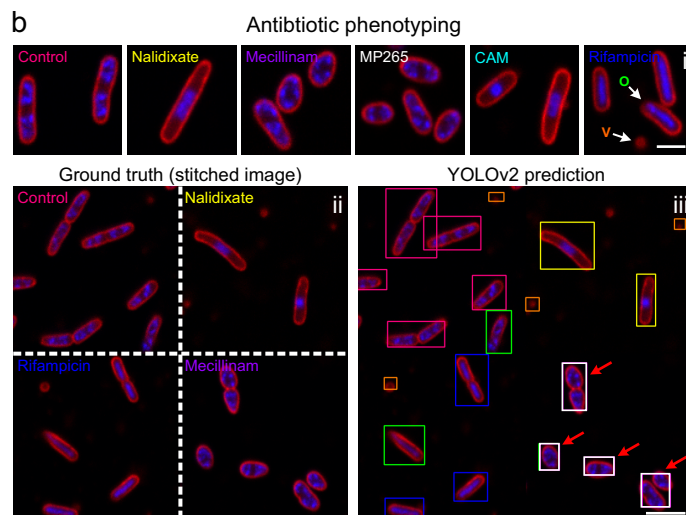

Supplement: Supplementary file 15 — Supplementary Data 1 [file 42003_2022_3634_MOESM15_ESM.zip › Figure_3/Figure_3.pdf]

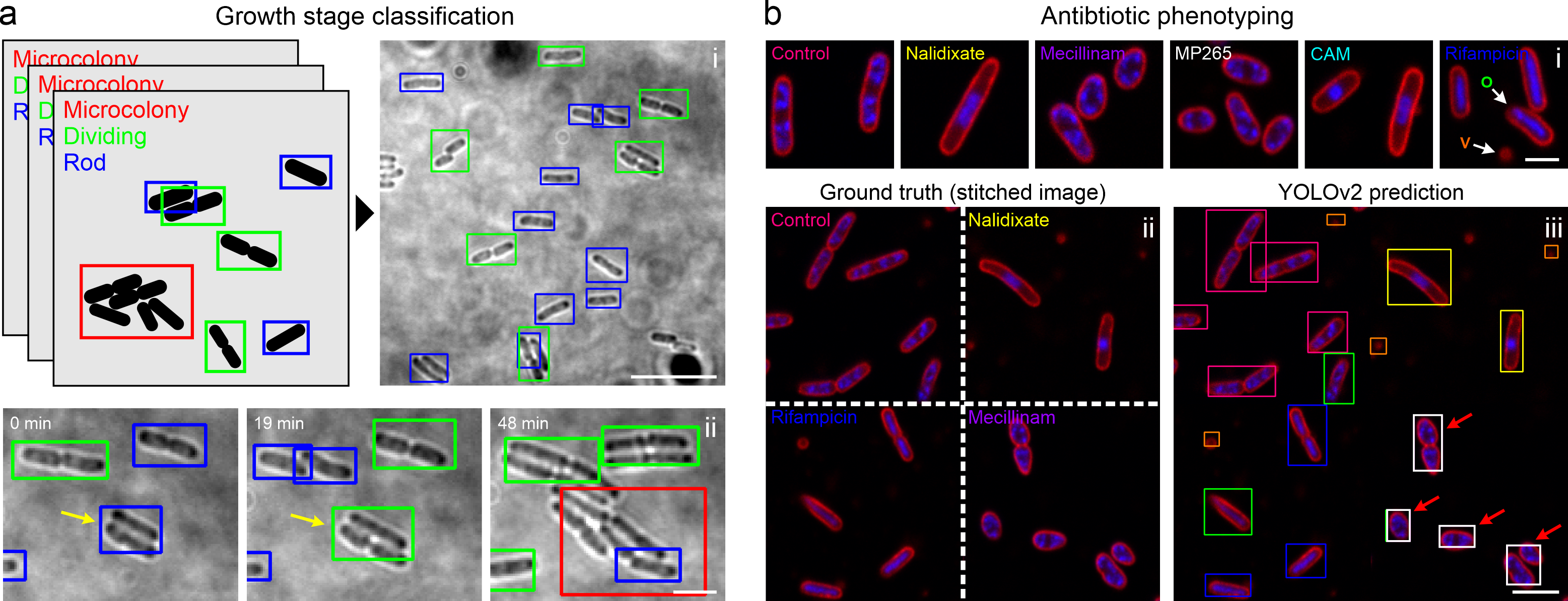

Supplement: Supplementary file 15 — Supplementary Data 1 [file 42003_2022_3634_MOESM15_ESM.zip › Figure_3/Figure_3.tif]

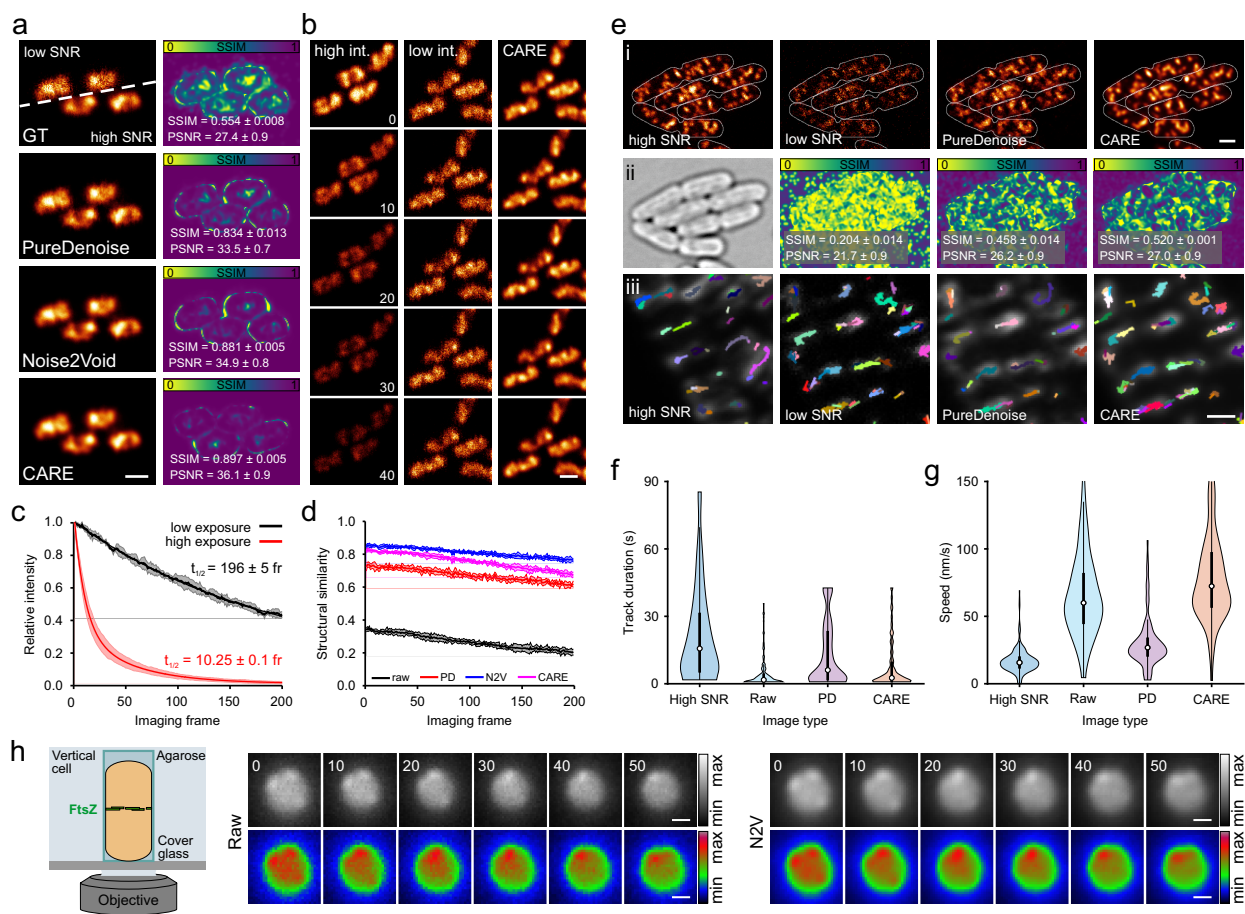

Supplement: Supplementary file 15 — Supplementary Data 1 [file 42003_2022_3634_MOESM15_ESM.zip › Figure_4/Figure_4.pdf]

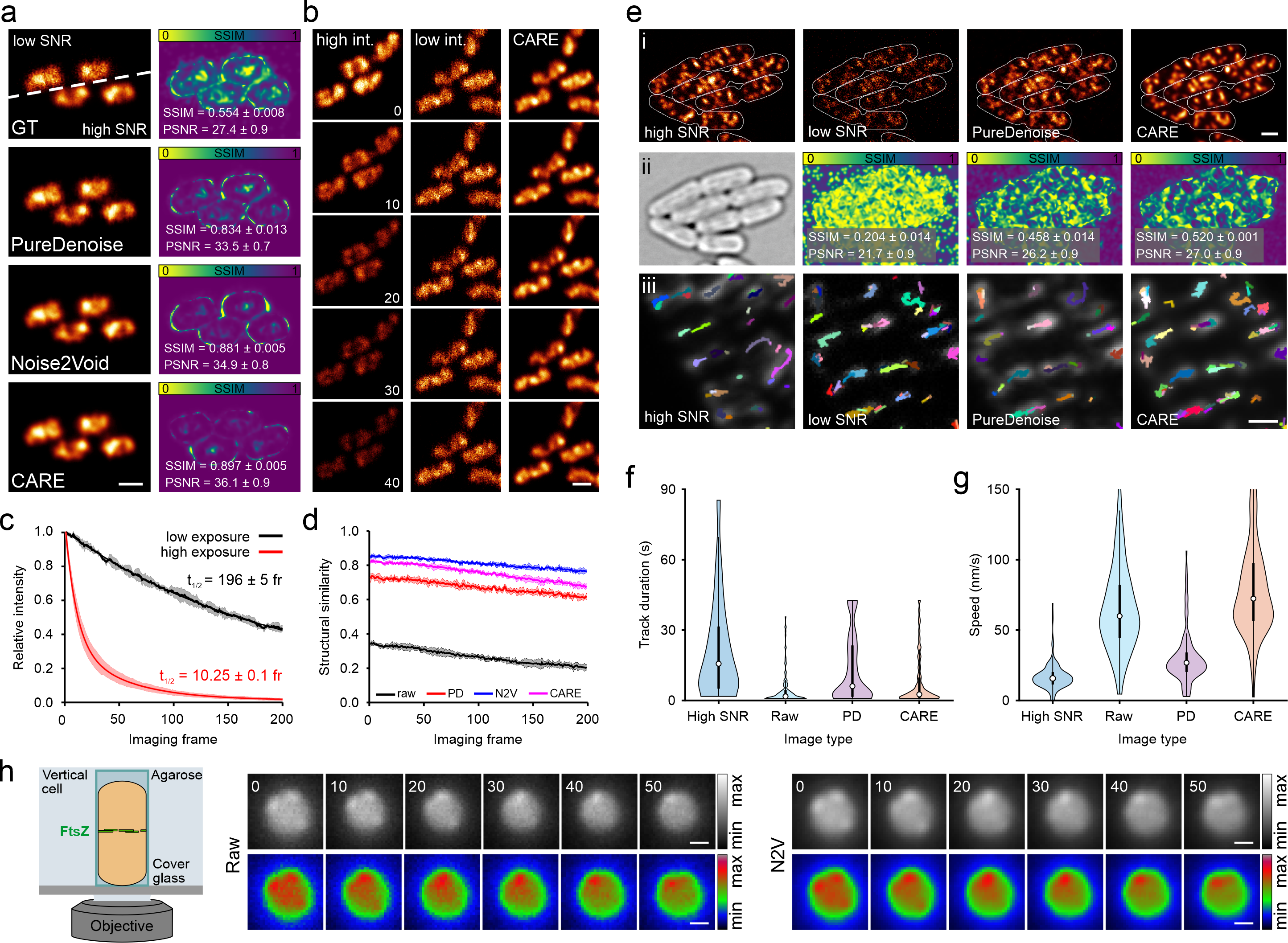

Supplement: Supplementary file 15 — Supplementary Data 1 [file 42003_2022_3634_MOESM15_ESM.zip › Figure_4/Figure_4.tif]

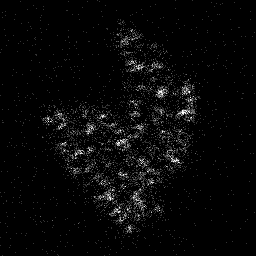

Supplement: Supplementary file 15 — Supplementary Data 1 [file 42003_2022_3634_MOESM15_ESM.zip › Figure_4/Figure_4e/Tracking/Time_series/fast_1_raw.tif]

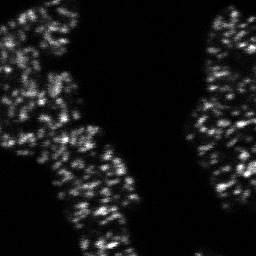

Supplement: Supplementary file 15 — Supplementary Data 1 [file 42003_2022_3634_MOESM15_ESM.zip › Figure_4/Figure_4e/Tracking/Time_series/MreB_high_SNR_fluorescence.tif]

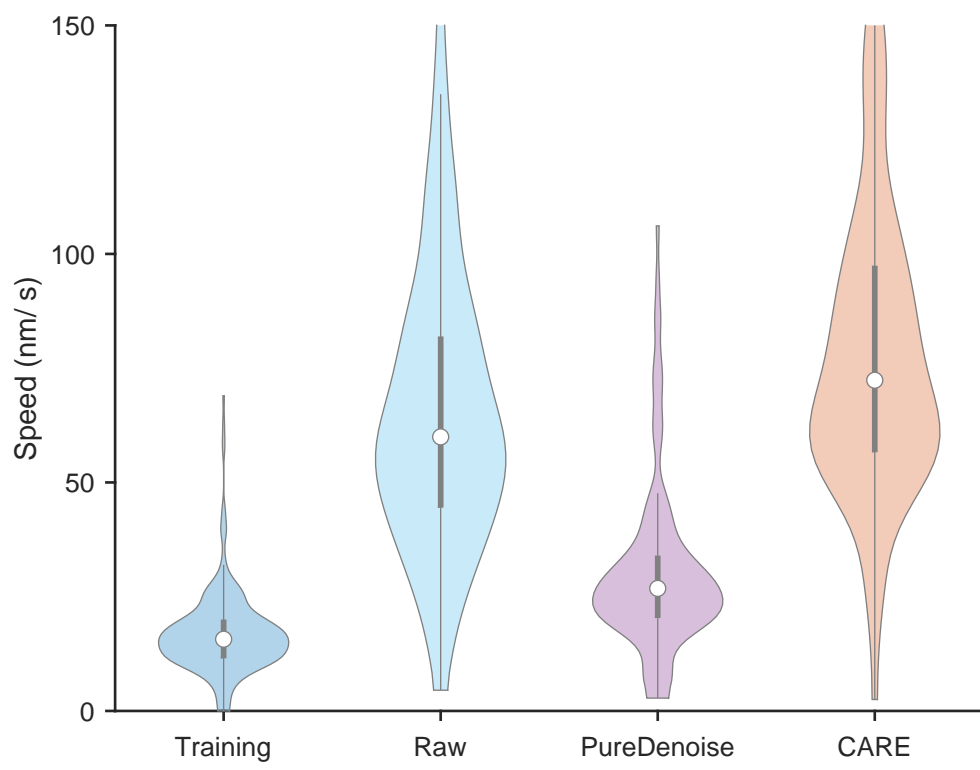

Supplement: Supplementary file 15 — Supplementary Data 1 [file 42003_2022_3634_MOESM15_ESM.zip › Figure_4/Figure_4fg_DeepBugs_MreB_tracking_analysis/MreB_speedMax150.pdf]

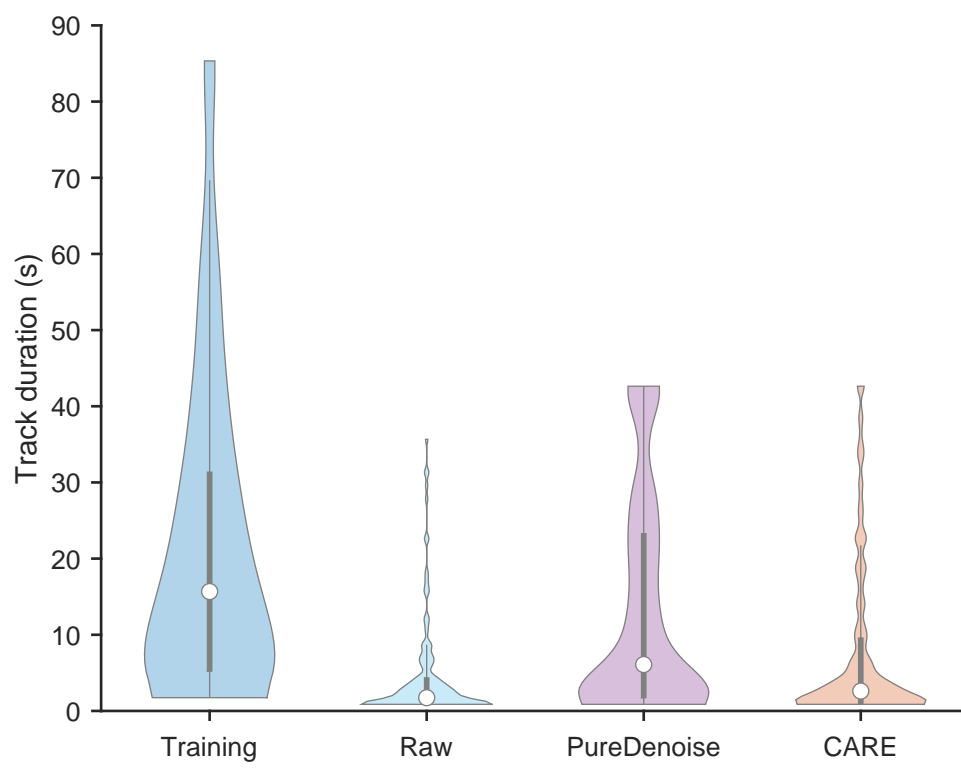

Supplement: Supplementary file 15 — Supplementary Data 1 [file 42003_2022_3634_MOESM15_ESM.zip › Figure_4/Figure_4fg_DeepBugs_MreB_tracking_analysis/MreB_track_length.pdf]

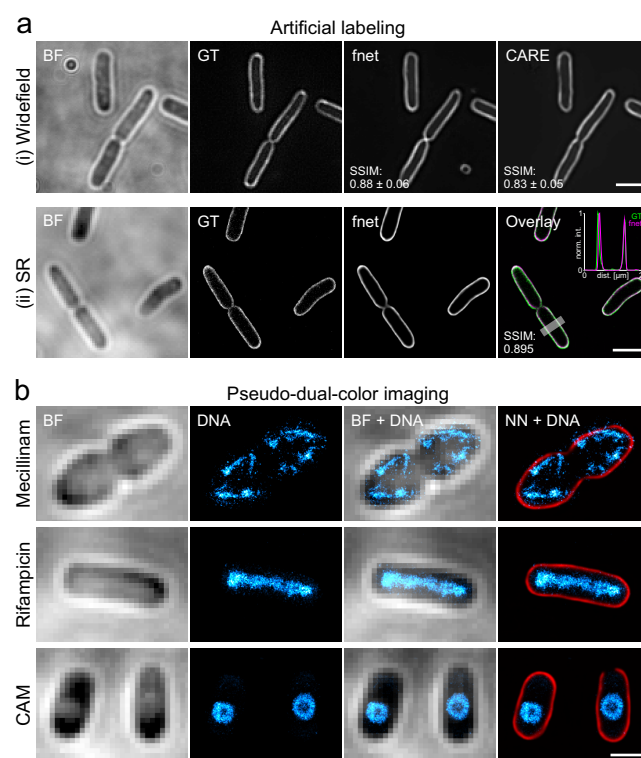

Supplement: Supplementary file 15 — Supplementary Data 1 [file 42003_2022_3634_MOESM15_ESM.zip › Figure_5/Figure_5.pdf]

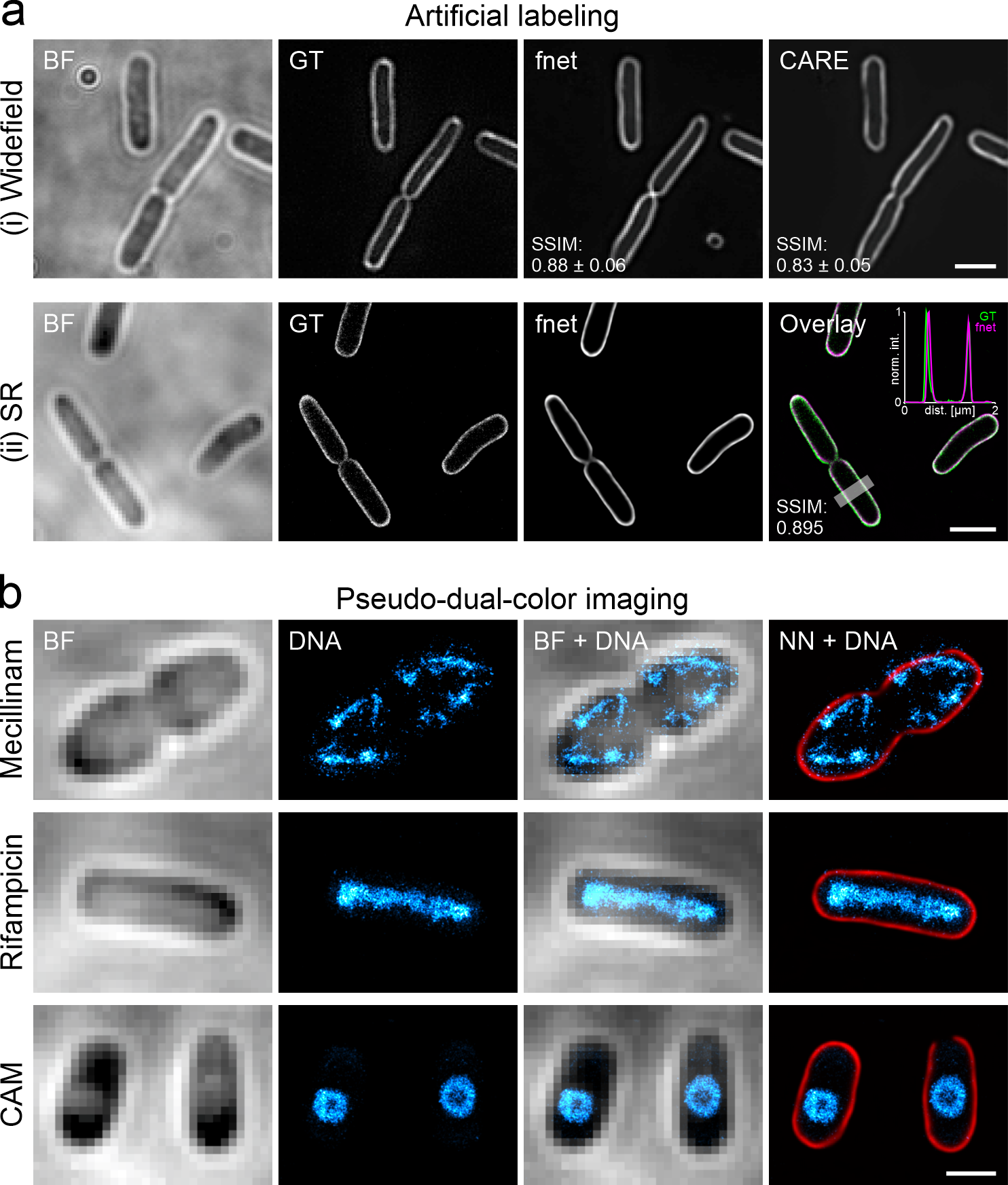

Supplement: Supplementary file 15 — Supplementary Data 1 [file 42003_2022_3634_MOESM15_ESM.zip › Figure_5/Figure_5.tif]

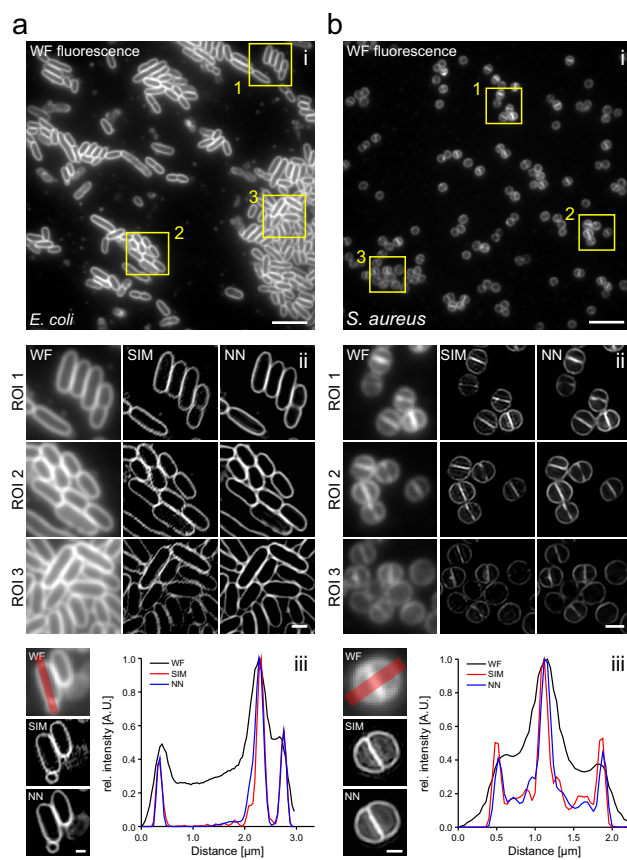

Supplement: Supplementary file 15 — Supplementary Data 1 [file 42003_2022_3634_MOESM15_ESM.zip › Figure_6/Figure_6.pdf]

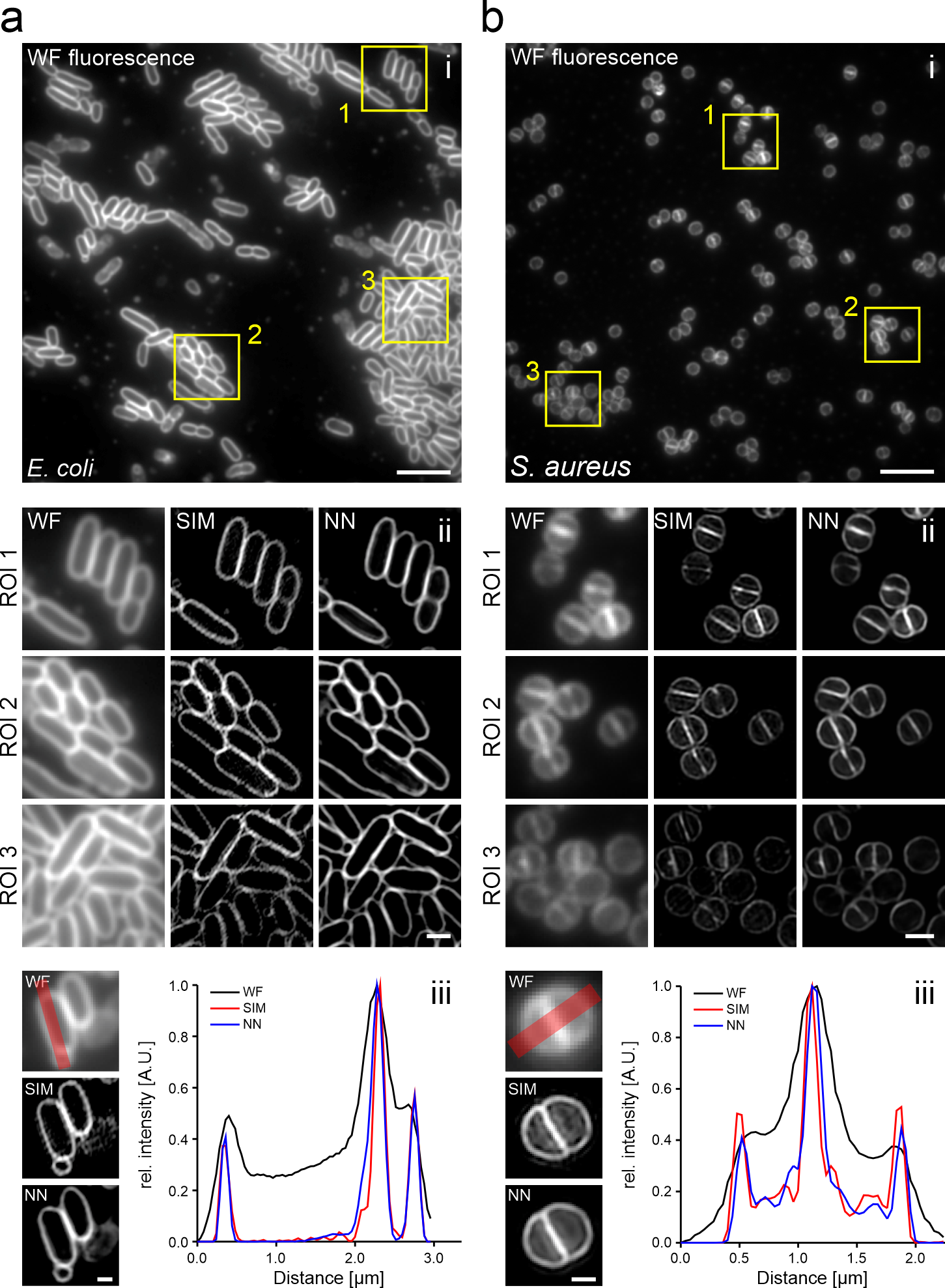

Supplement: Supplementary file 15 — Supplementary Data 1 [file 42003_2022_3634_MOESM15_ESM.zip › Figure_6/Figure_6.tif]

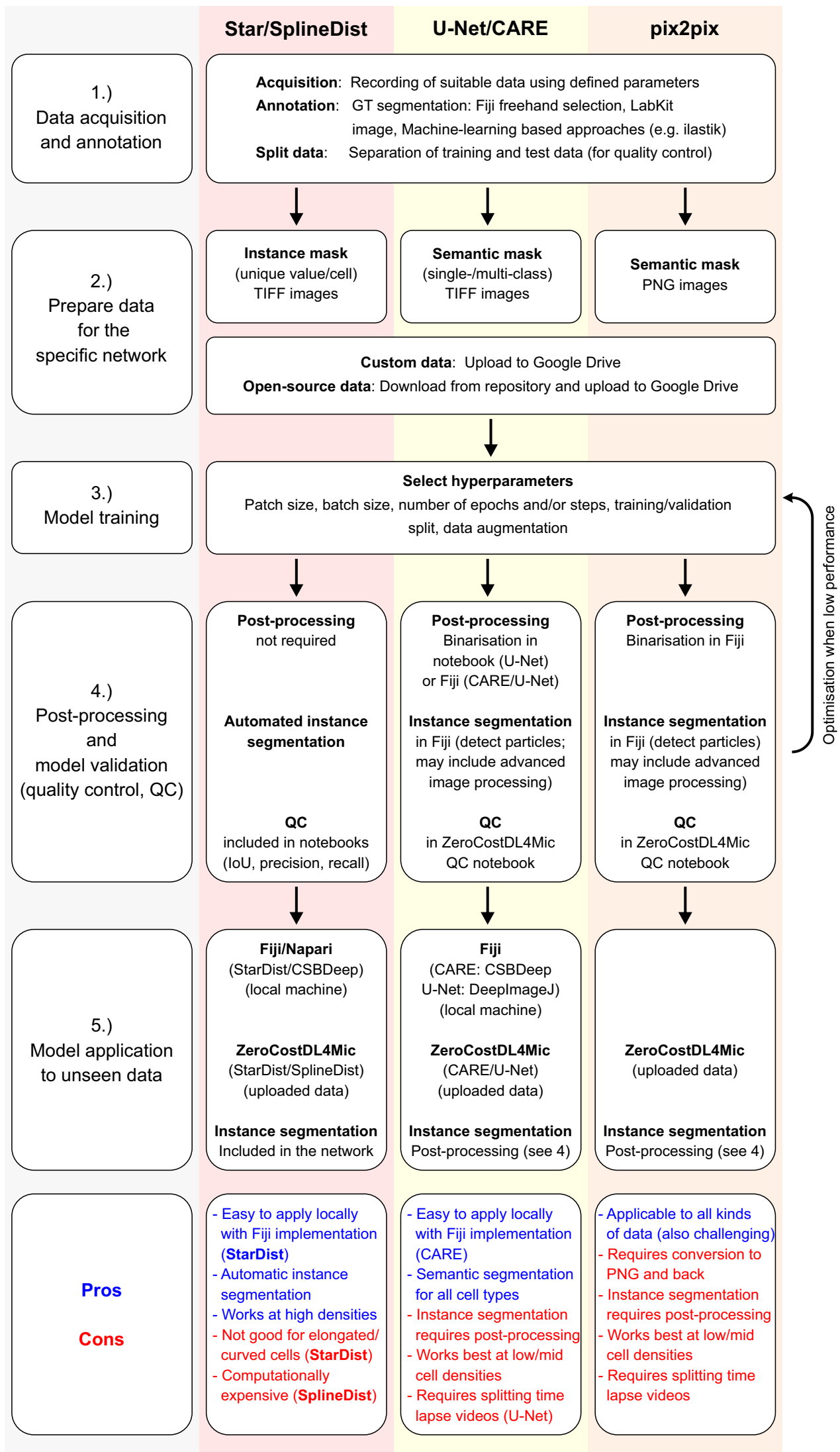

Supplement: Supplementary file 15 — Supplementary Data 1 [file 42003_2022_3634_MOESM15_ESM.zip › Figure_S1/Figure_S1.pdf]

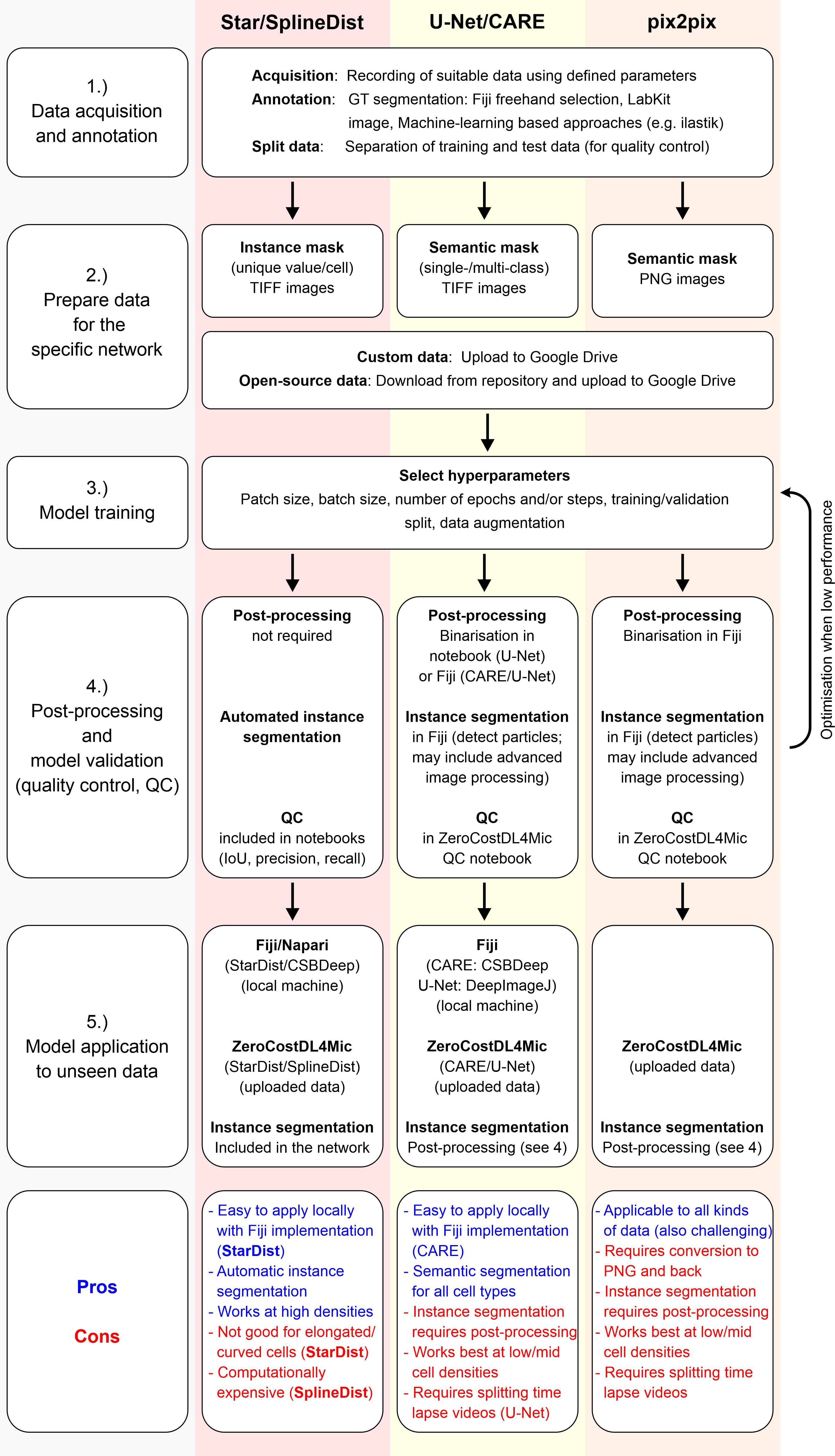

Supplement: Supplementary file 15 — Supplementary Data 1 [file 42003_2022_3634_MOESM15_ESM.zip › Figure_S1/Figure_S1.tif]

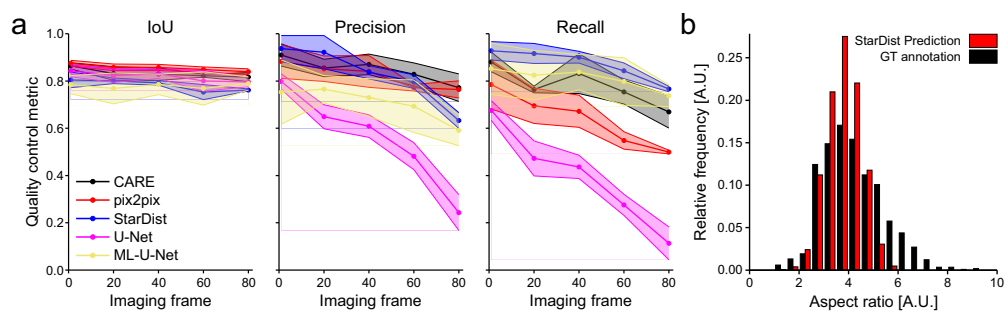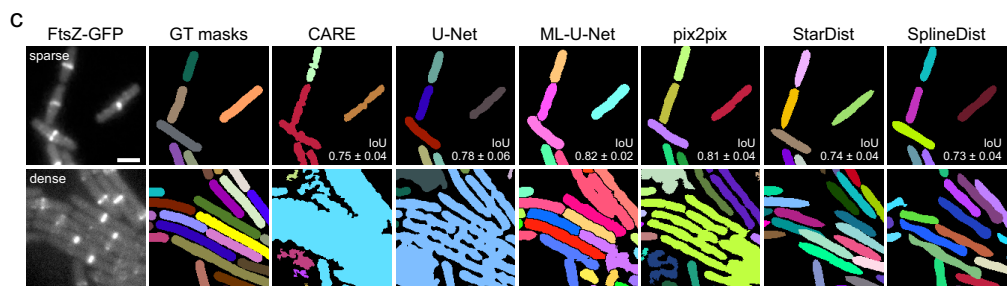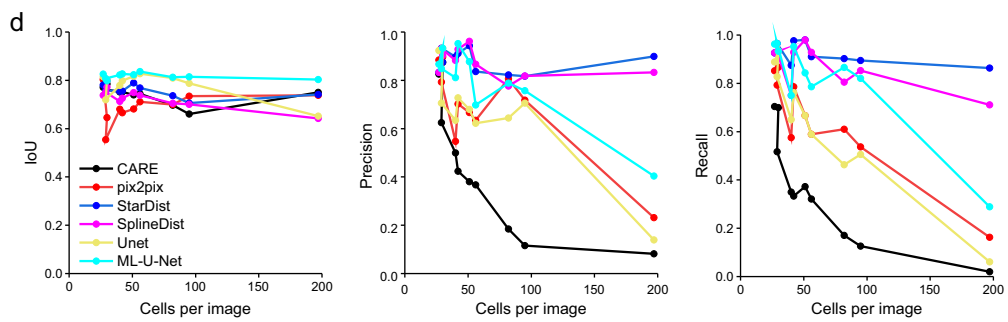

Supplement: Supplementary file 15 — Supplementary Data 1 [file 42003_2022_3634_MOESM15_ESM.zip › Figure_S2/Figure_S2.pdf]

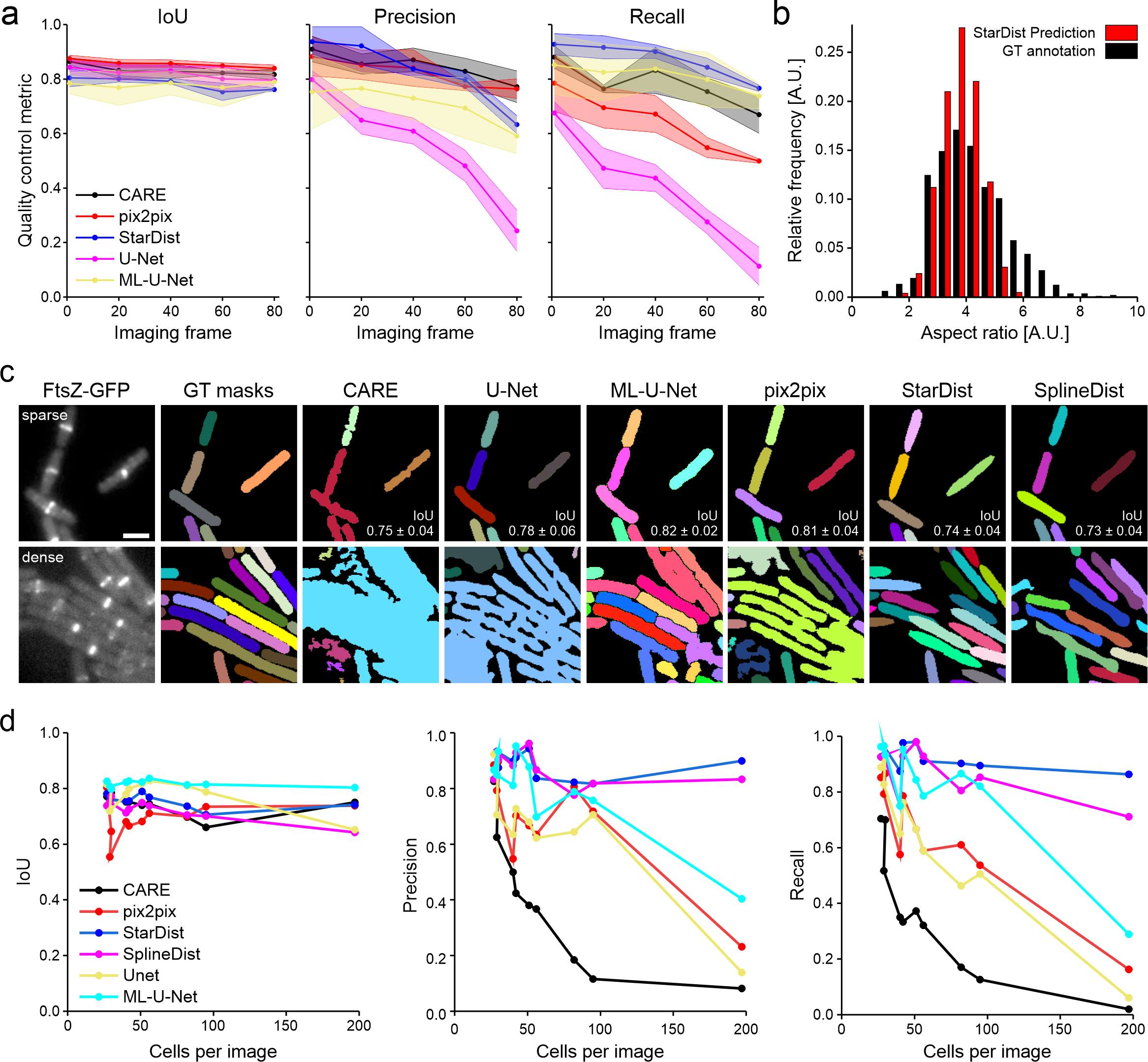

Supplement: Supplementary file 15 — Supplementary Data 1 [file 42003_2022_3634_MOESM15_ESM.zip › Figure_S2/Figure_S2.tif]

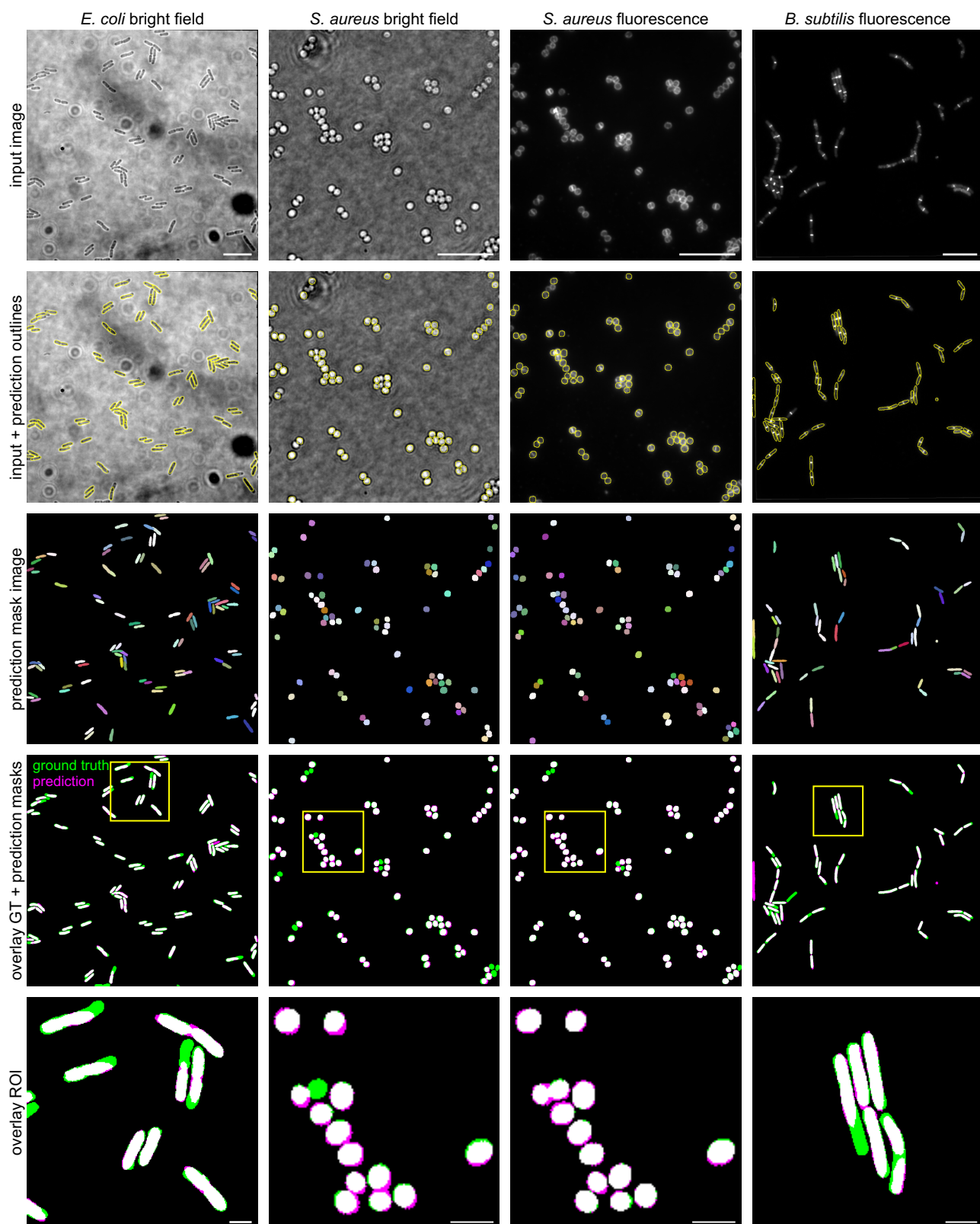

Supplement: Supplementary file 15 — Supplementary Data 1 [file 42003_2022_3634_MOESM15_ESM.zip › Figure_S3/Figure_S3.pdf]

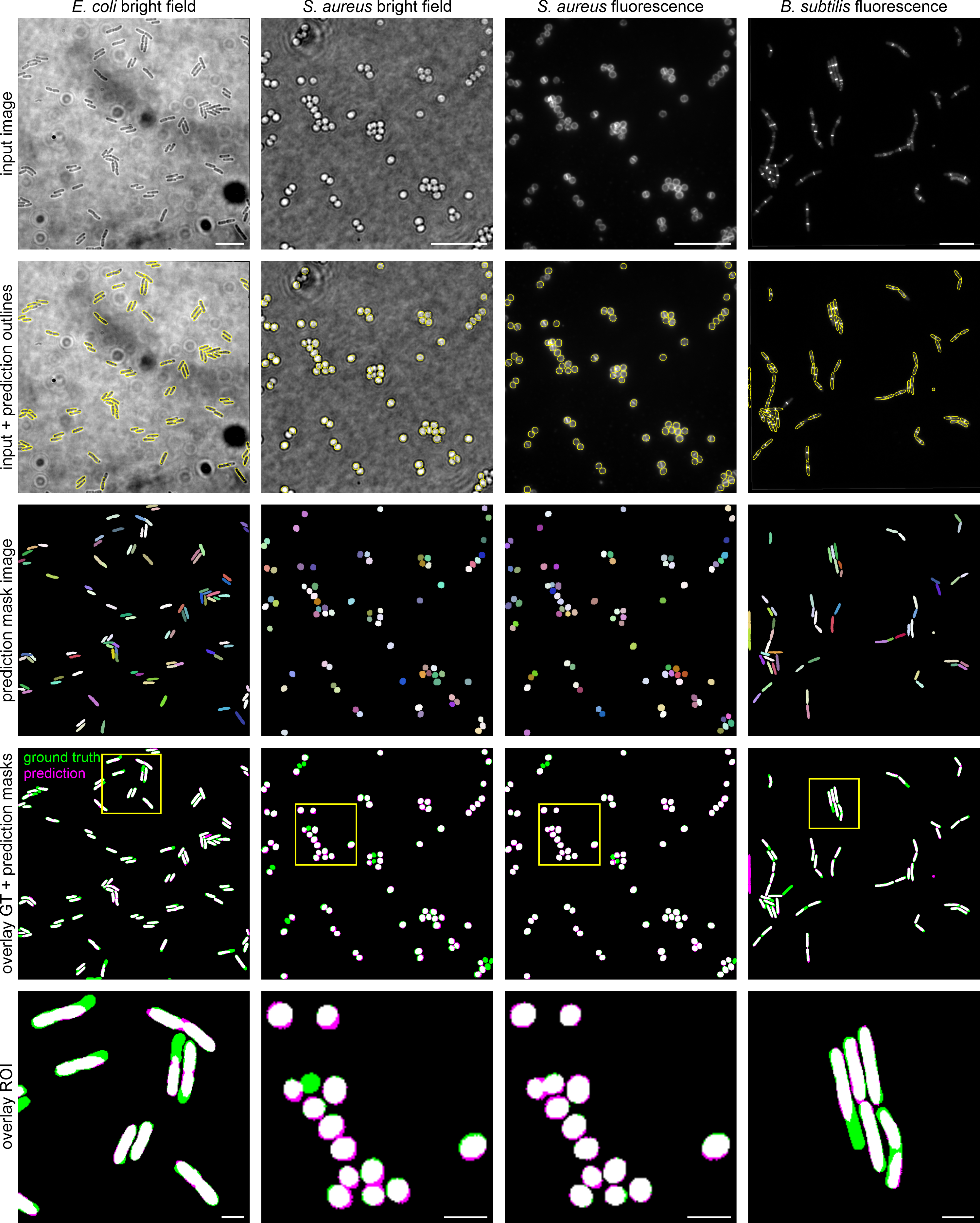

Supplement: Supplementary file 15 — Supplementary Data 1 [file 42003_2022_3634_MOESM15_ESM.zip › Figure_S3/Figure_S3.tif]

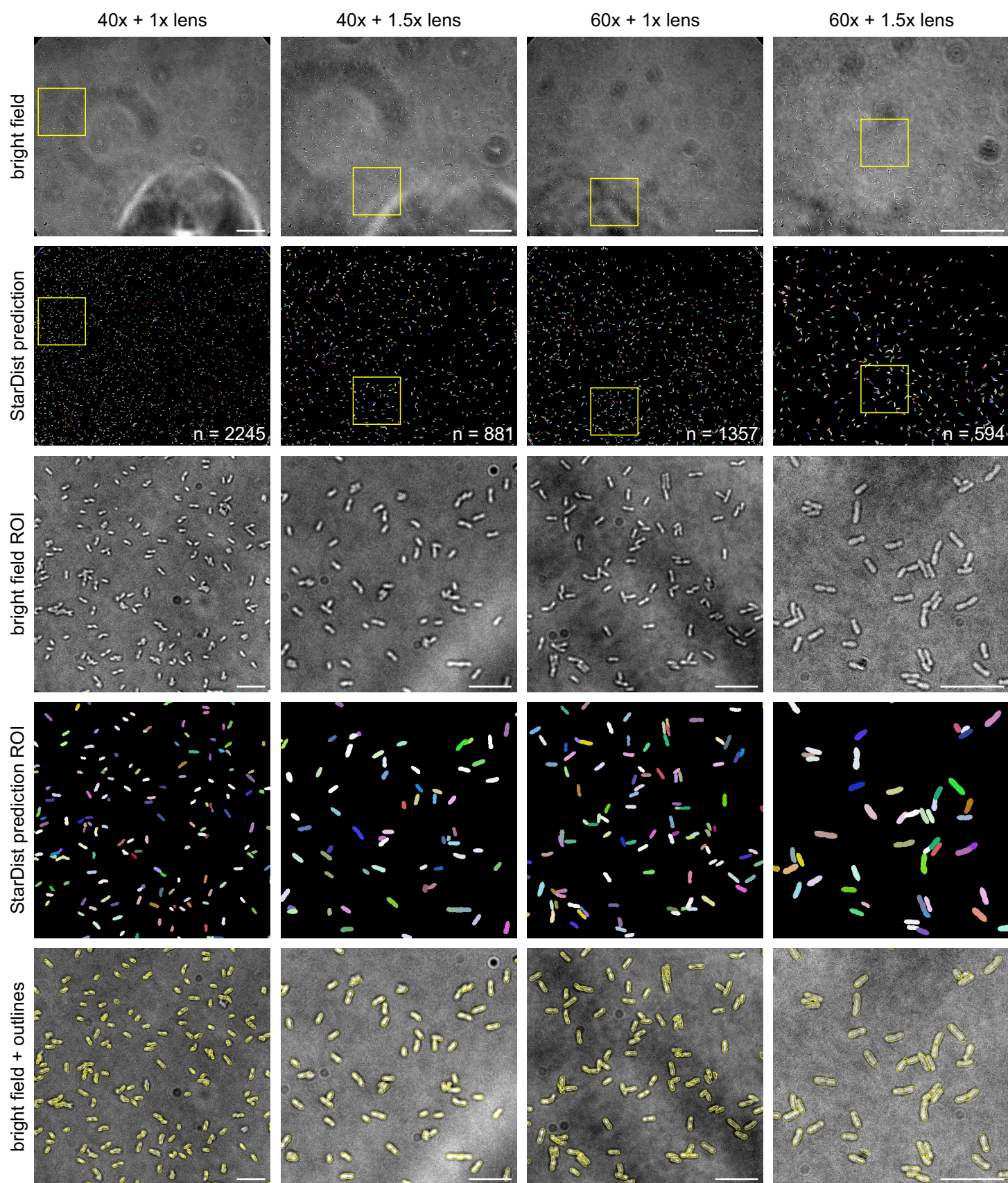

Supplement: Supplementary file 15 — Supplementary Data 1 [file 42003_2022_3634_MOESM15_ESM.zip › Figure_S4/Figure_S4.pdf]

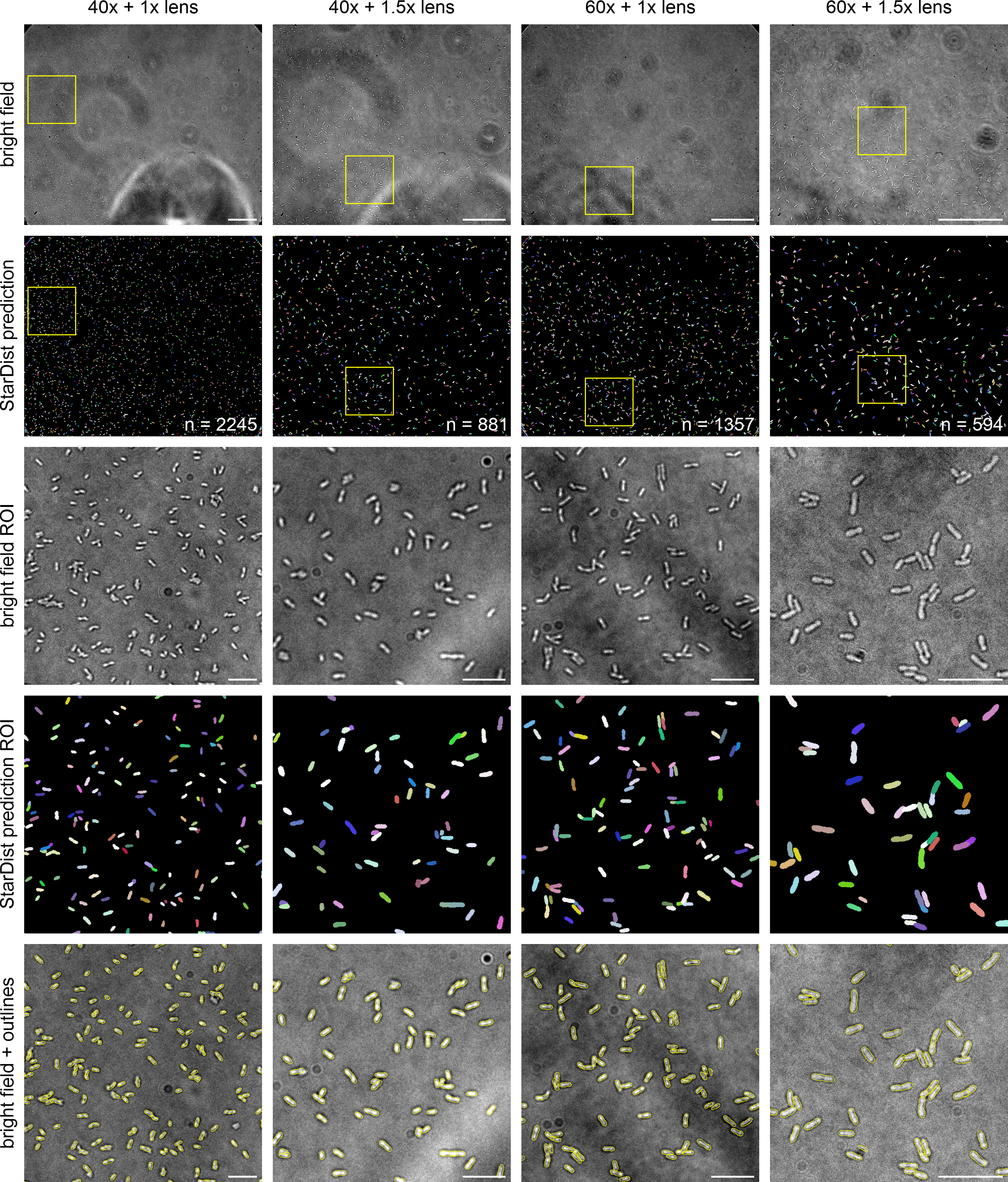

Supplement: Supplementary file 15 — Supplementary Data 1 [file 42003_2022_3634_MOESM15_ESM.zip › Figure_S4/Figure_S4.tif]

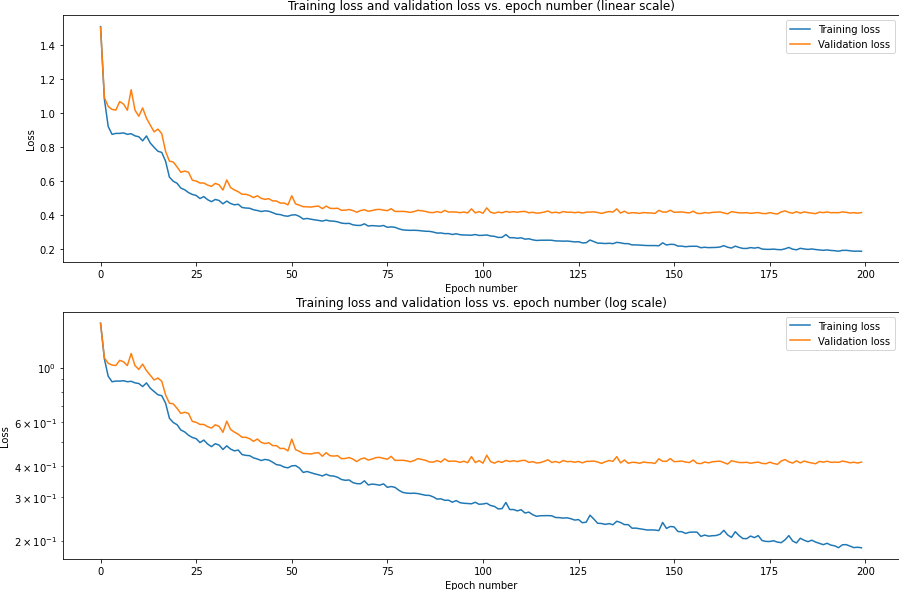

Supplement: Supplementary file 15 — Supplementary Data 1 [file 42003_2022_3634_MOESM15_ESM.zip › Figure_S4/Model_validation/lossCurvePlots.png]

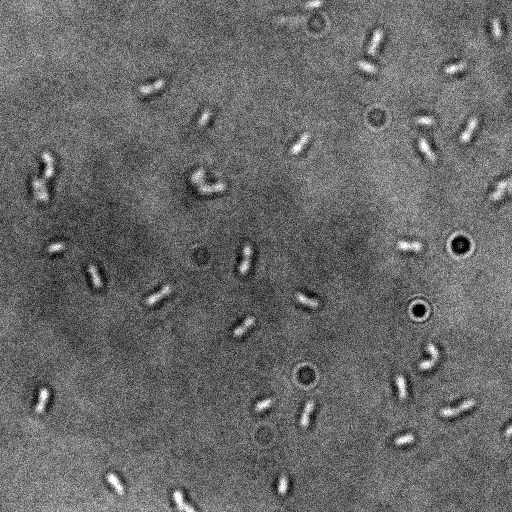

Supplement: Supplementary file 15 — Supplementary Data 1 [file 42003_2022_3634_MOESM15_ESM.zip › Figure_S4/Training_data/Brightfield/At_BF_40X_1,5x_1_ROI_1.tif]

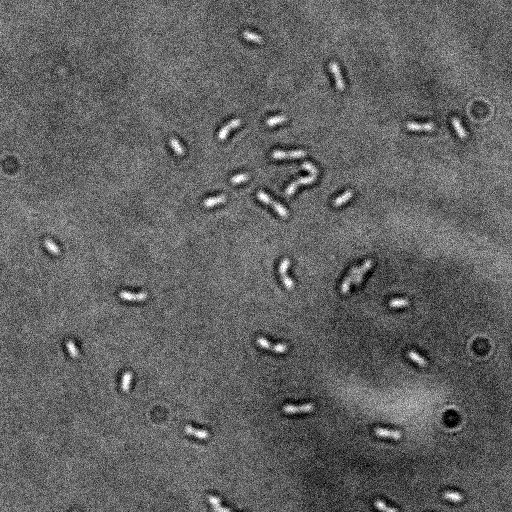

Supplement: Supplementary file 15 — Supplementary Data 1 [file 42003_2022_3634_MOESM15_ESM.zip › Figure_S4/Training_data/Brightfield/At_BF_40X_1,5x_1_ROI_2.tif]

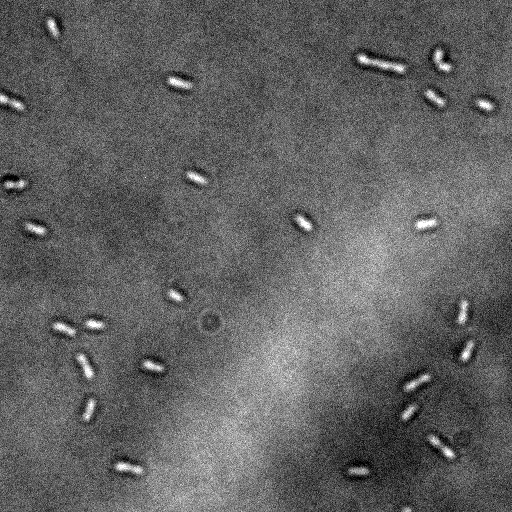

Supplement: Supplementary file 15 — Supplementary Data 1 [file 42003_2022_3634_MOESM15_ESM.zip › Figure_S4/Training_data/Brightfield/At_BF_40X_1,5x_1_ROI_3.tif]

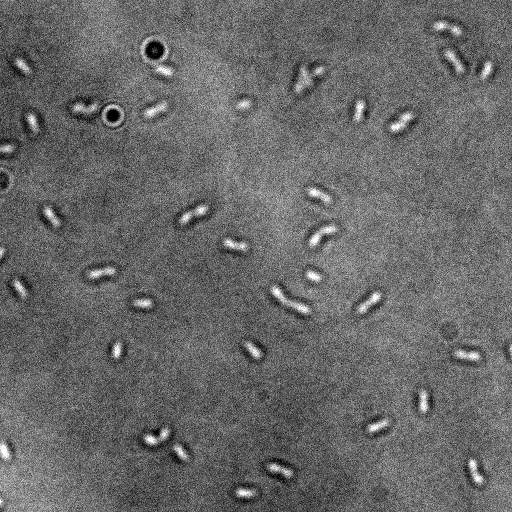

Supplement: Supplementary file 15 — Supplementary Data 1 [file 42003_2022_3634_MOESM15_ESM.zip › Figure_S4/Training_data/Brightfield/At_BF_40X_1,5x_2_ROI_1.tif]

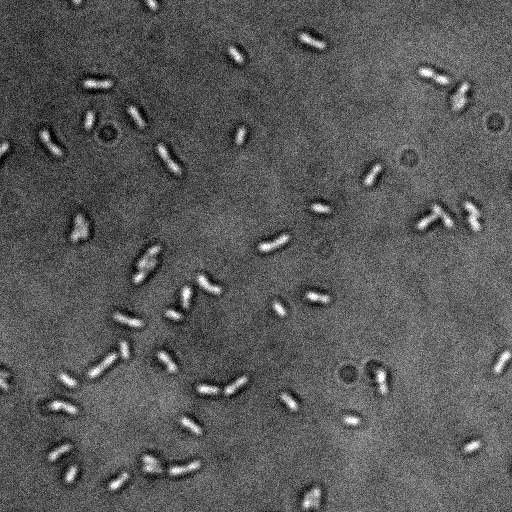

Supplement: Supplementary file 15 — Supplementary Data 1 [file 42003_2022_3634_MOESM15_ESM.zip › Figure_S4/Training_data/Brightfield/At_BF_40X_1,5x_2_ROI_2.tif]

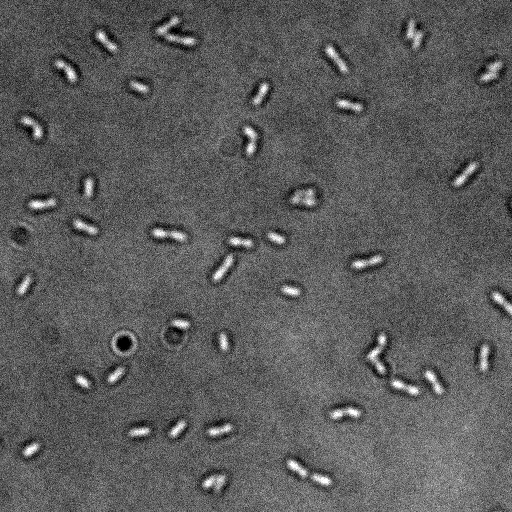

Supplement: Supplementary file 15 — Supplementary Data 1 [file 42003_2022_3634_MOESM15_ESM.zip › Figure_S4/Training_data/Brightfield/At_BF_40X_1,5x_2_ROI_3.tif]

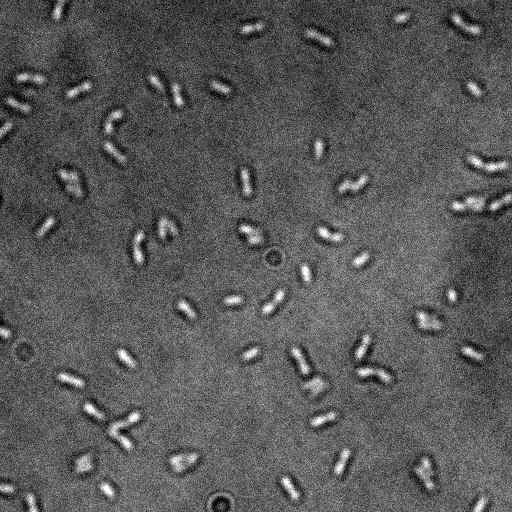

Supplement: Supplementary file 15 — Supplementary Data 1 [file 42003_2022_3634_MOESM15_ESM.zip › Figure_S4/Training_data/Brightfield/At_BF_40X_1,5x_3_ROI_1.tif]

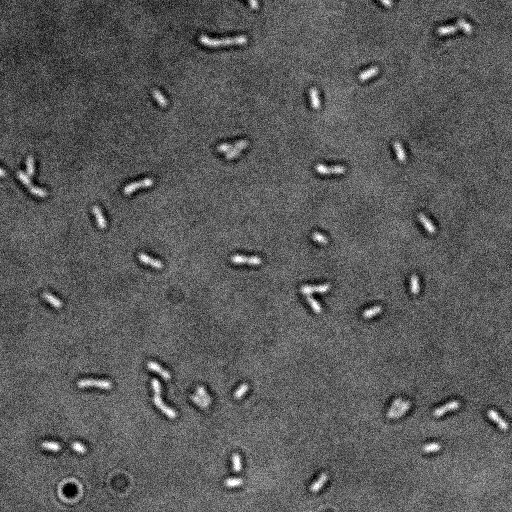

Supplement: Supplementary file 15 — Supplementary Data 1 [file 42003_2022_3634_MOESM15_ESM.zip › Figure_S4/Training_data/Brightfield/At_BF_40X_1,5x_3_ROI_2.tif]

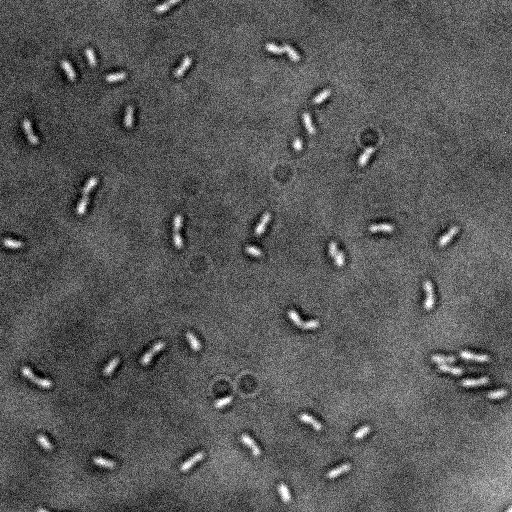

Supplement: Supplementary file 15 — Supplementary Data 1 [file 42003_2022_3634_MOESM15_ESM.zip › Figure_S4/Training_data/Brightfield/At_BF_40X_1,5x_3_ROI_3.tif]

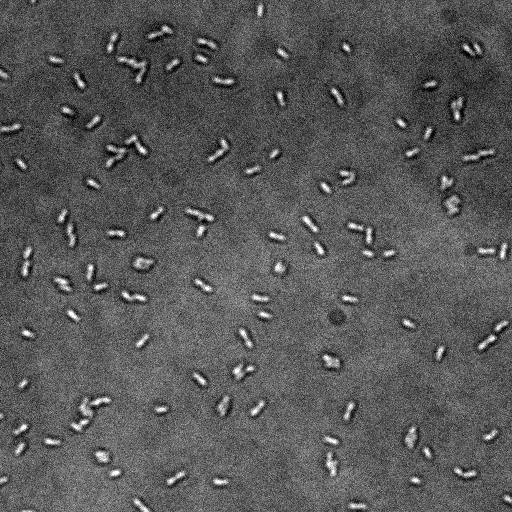

Supplement: Supplementary file 15 — Supplementary Data 1 [file 42003_2022_3634_MOESM15_ESM.zip › Figure_S4/Training_data/Brightfield/At_BF_40X_1x_1_ROI_1.tif]

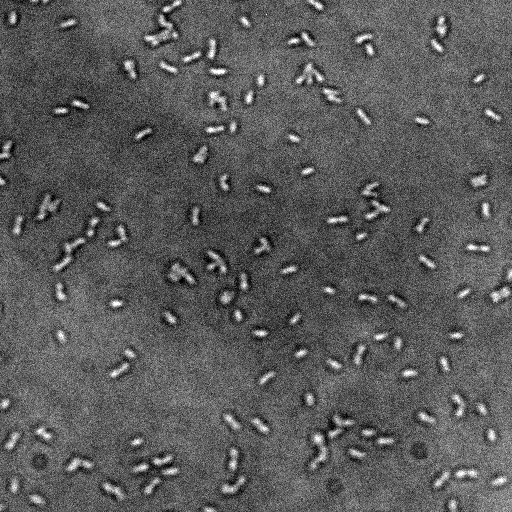

Supplement: Supplementary file 15 — Supplementary Data 1 [file 42003_2022_3634_MOESM15_ESM.zip › Figure_S4/Training_data/Brightfield/At_BF_40X_1x_1_ROI_2.tif]

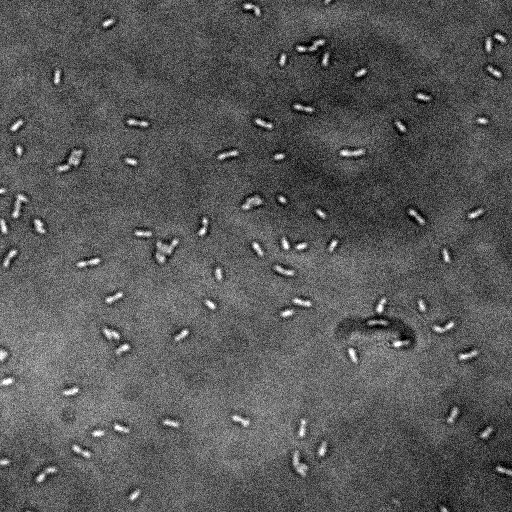

Supplement: Supplementary file 15 — Supplementary Data 1 [file 42003_2022_3634_MOESM15_ESM.zip › Figure_S4/Training_data/Brightfield/At_BF_40X_1x_1_ROI_3.tif]

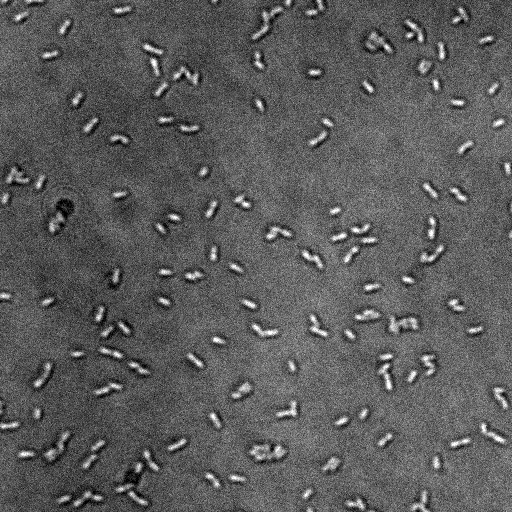

Supplement: Supplementary file 15 — Supplementary Data 1 [file 42003_2022_3634_MOESM15_ESM.zip › Figure_S4/Training_data/Brightfield/At_BF_40X_1x_2_ROI_1.tif]

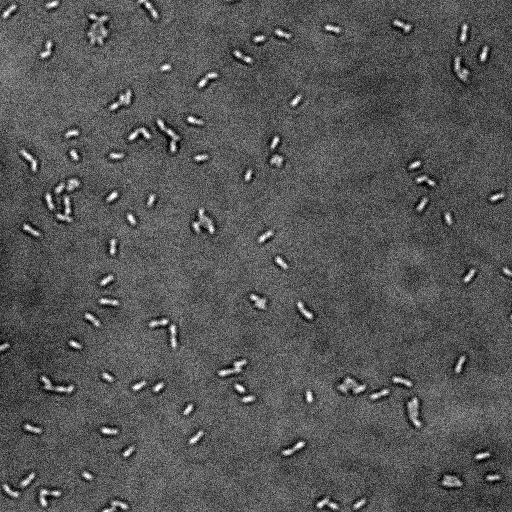

Supplement: Supplementary file 15 — Supplementary Data 1 [file 42003_2022_3634_MOESM15_ESM.zip › Figure_S4/Training_data/Brightfield/At_BF_40X_1x_2_ROI_2.tif]

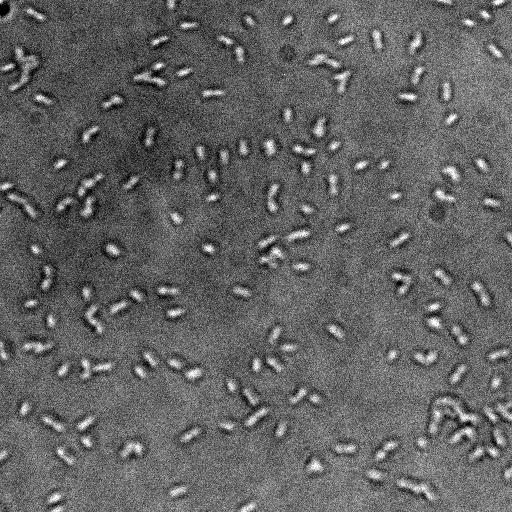

Supplement: Supplementary file 15 — Supplementary Data 1 [file 42003_2022_3634_MOESM15_ESM.zip › Figure_S4/Training_data/Brightfield/At_BF_40X_1x_2_ROI_3.tif]

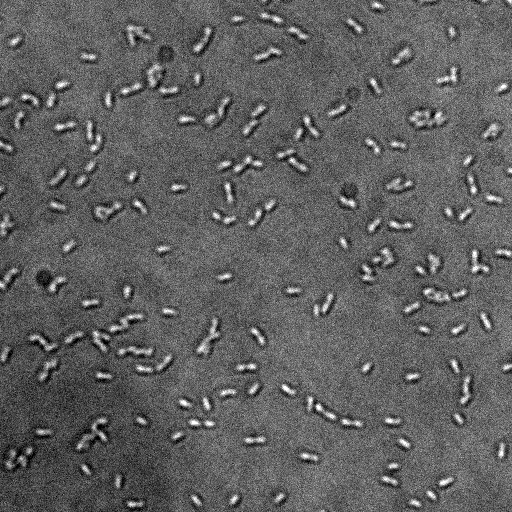

Supplement: Supplementary file 15 — Supplementary Data 1 [file 42003_2022_3634_MOESM15_ESM.zip › Figure_S4/Training_data/Brightfield/At_BF_40X_1x_3_ROI_1.tif]

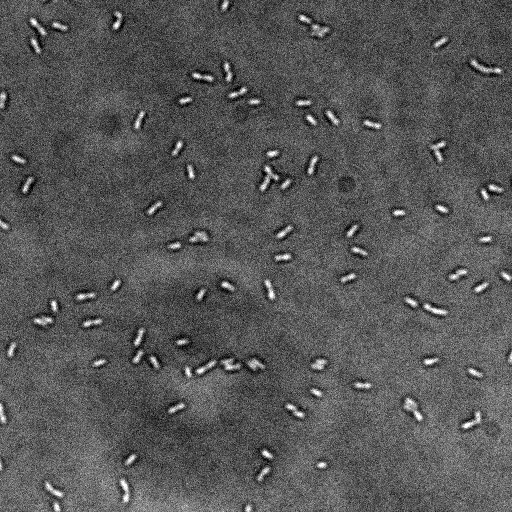

Supplement: Supplementary file 15 — Supplementary Data 1 [file 42003_2022_3634_MOESM15_ESM.zip › Figure_S4/Training_data/Brightfield/At_BF_40X_1x_3_ROI_2.tif]

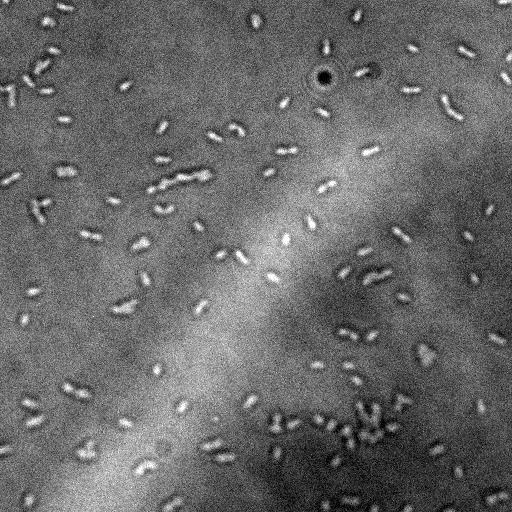

Supplement: Supplementary file 15 — Supplementary Data 1 [file 42003_2022_3634_MOESM15_ESM.zip › Figure_S4/Training_data/Brightfield/At_BF_40X_1x_3_ROI_3.tif]

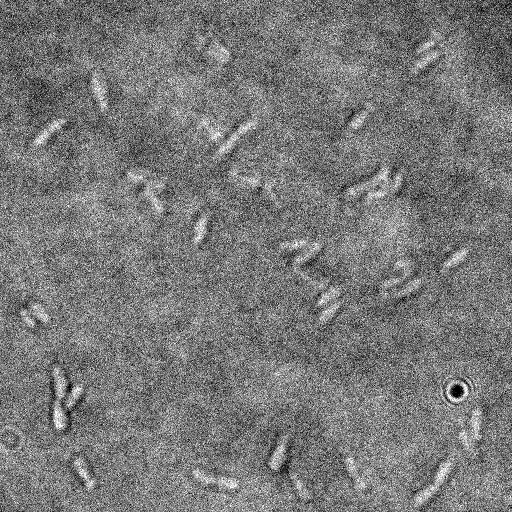

Supplement: Supplementary file 15 — Supplementary Data 1 [file 42003_2022_3634_MOESM15_ESM.zip › Figure_S4/Training_data/Brightfield/At_BF_60X_1,5x_1_ROI_1.tif]

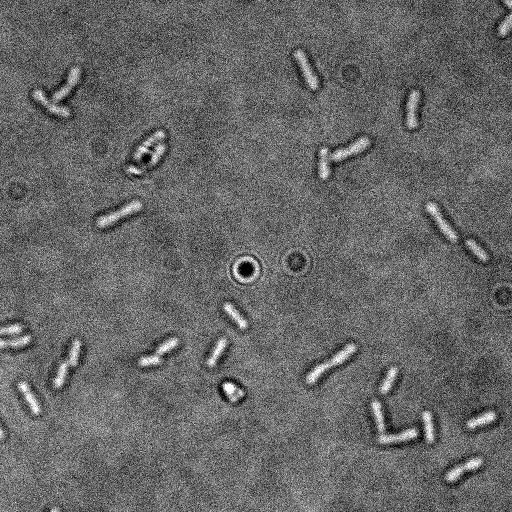

Supplement: Supplementary file 15 — Supplementary Data 1 [file 42003_2022_3634_MOESM15_ESM.zip › Figure_S4/Training_data/Brightfield/At_BF_60X_1,5x_1_ROI_2.tif]

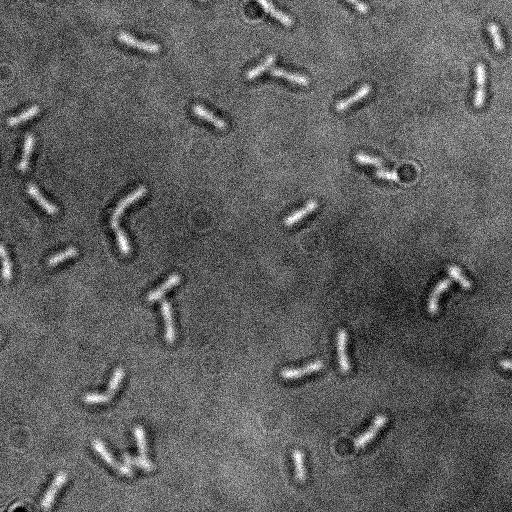

Supplement: Supplementary file 15 — Supplementary Data 1 [file 42003_2022_3634_MOESM15_ESM.zip › Figure_S4/Training_data/Brightfield/At_BF_60X_1,5x_1_ROI_3.tif]

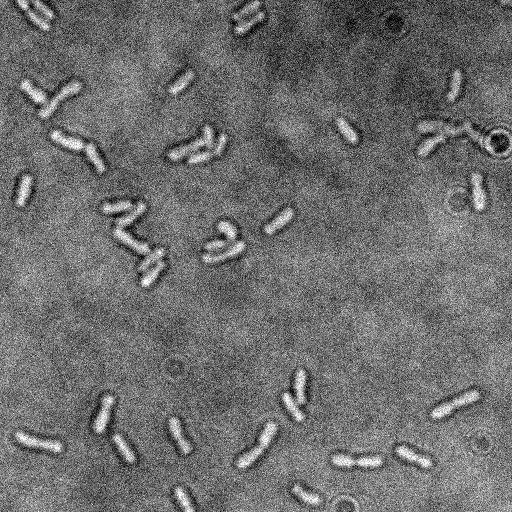

Supplement: Supplementary file 15 — Supplementary Data 1 [file 42003_2022_3634_MOESM15_ESM.zip › Figure_S4/Training_data/Brightfield/At_BF_60X_1,5x_2_ROI_1.tif]

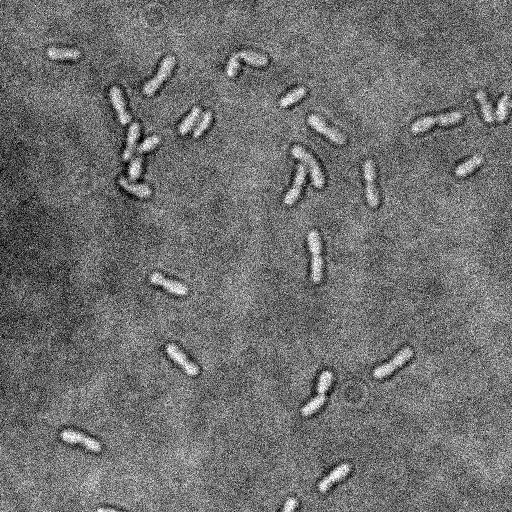

Supplement: Supplementary file 15 — Supplementary Data 1 [file 42003_2022_3634_MOESM15_ESM.zip › Figure_S4/Training_data/Brightfield/At_BF_60X_1,5x_2_ROI_2.tif]

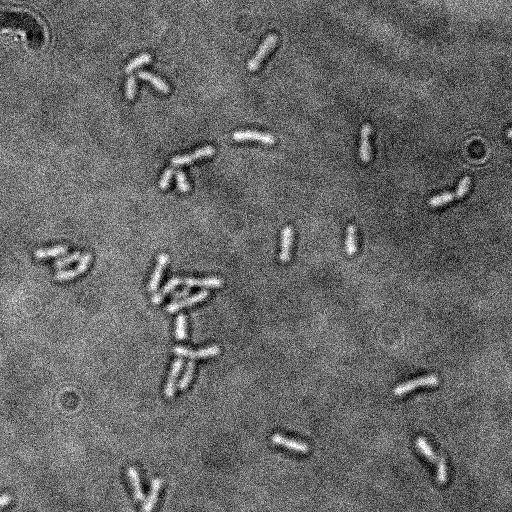

Supplement: Supplementary file 15 — Supplementary Data 1 [file 42003_2022_3634_MOESM15_ESM.zip › Figure_S4/Training_data/Brightfield/At_BF_60X_1,5x_2_ROI_3.tif]

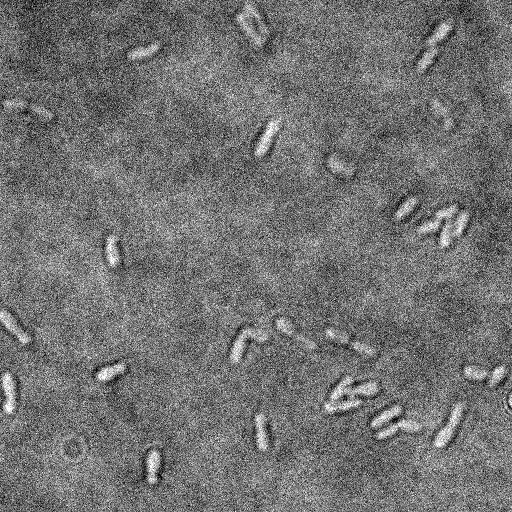

Supplement: Supplementary file 15 — Supplementary Data 1 [file 42003_2022_3634_MOESM15_ESM.zip › Figure_S4/Training_data/Brightfield/At_BF_60X_1,5x_3_ROI_1.tif]

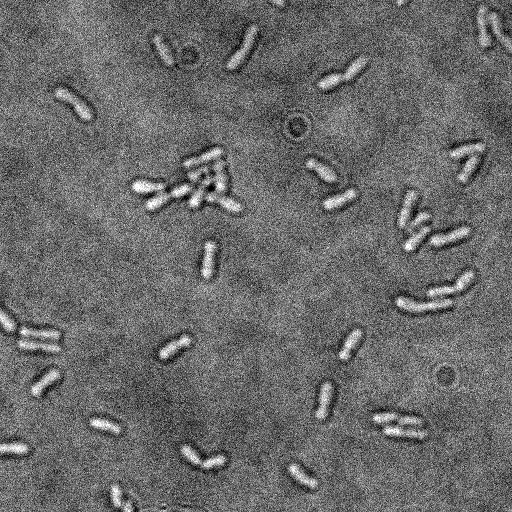

Supplement: Supplementary file 15 — Supplementary Data 1 [file 42003_2022_3634_MOESM15_ESM.zip › Figure_S4/Training_data/Brightfield/At_BF_60X_1,5x_3_ROI_2.tif]

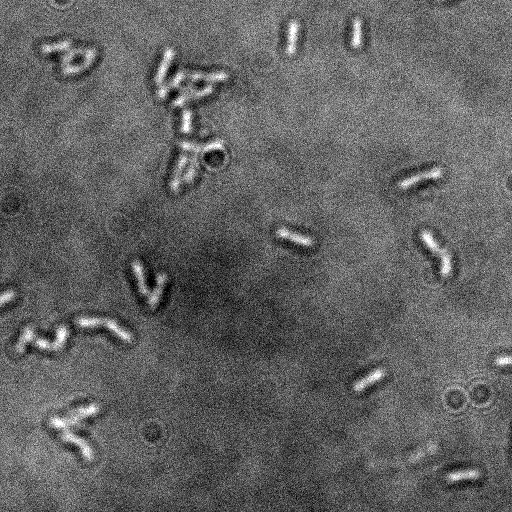

Supplement: Supplementary file 15 — Supplementary Data 1 [file 42003_2022_3634_MOESM15_ESM.zip › Figure_S4/Training_data/Brightfield/At_BF_60X_1,5x_3_ROI_3.tif]

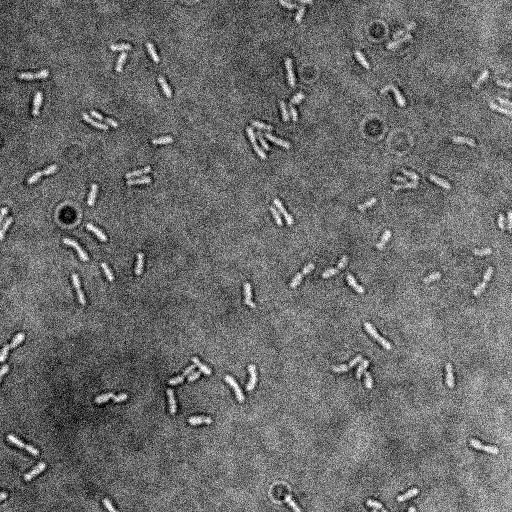

Supplement: Supplementary file 15 — Supplementary Data 1 [file 42003_2022_3634_MOESM15_ESM.zip › Figure_S4/Training_data/Brightfield/At_BF_60X_1x_1_ROI_1.tif]

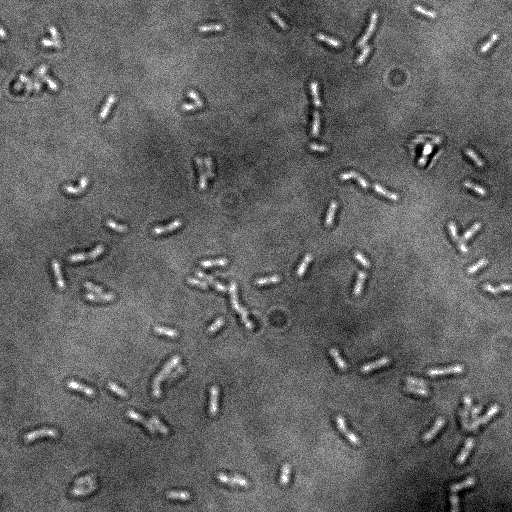

Supplement: Supplementary file 15 — Supplementary Data 1 [file 42003_2022_3634_MOESM15_ESM.zip › Figure_S4/Training_data/Brightfield/At_BF_60X_1x_1_ROI_2.tif]

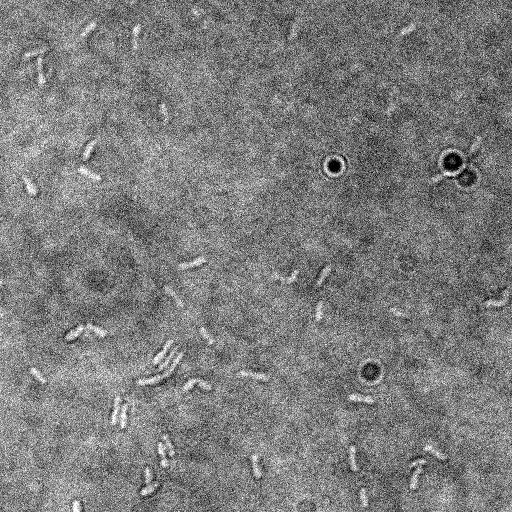

Supplement: Supplementary file 15 — Supplementary Data 1 [file 42003_2022_3634_MOESM15_ESM.zip › Figure_S4/Training_data/Brightfield/At_BF_60X_1x_1_ROI_3.tif]

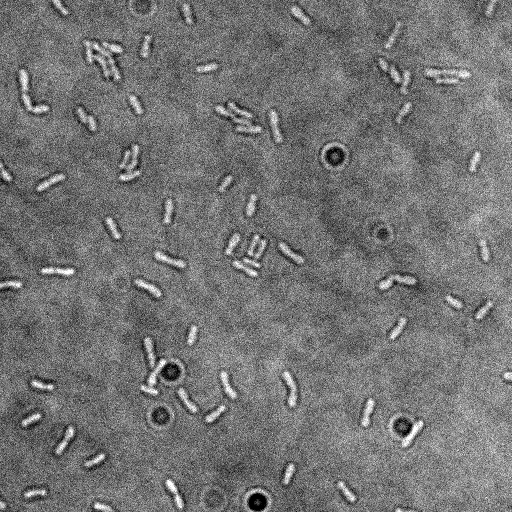

Supplement: Supplementary file 15 — Supplementary Data 1 [file 42003_2022_3634_MOESM15_ESM.zip › Figure_S4/Training_data/Brightfield/At_BF_60X_1x_2_ROI_1.tif]

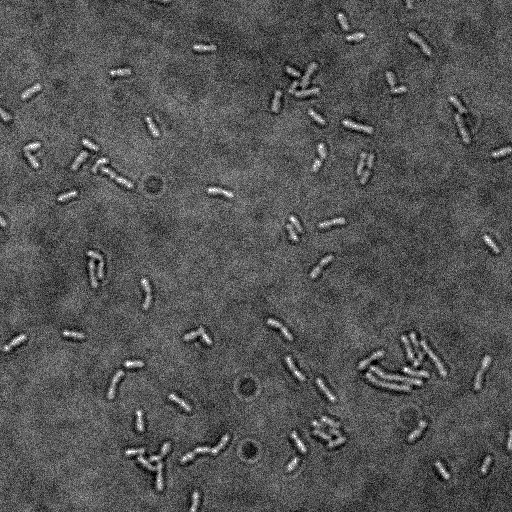

Supplement: Supplementary file 15 — Supplementary Data 1 [file 42003_2022_3634_MOESM15_ESM.zip › Figure_S4/Training_data/Brightfield/At_BF_60X_1x_2_ROI_2.tif]

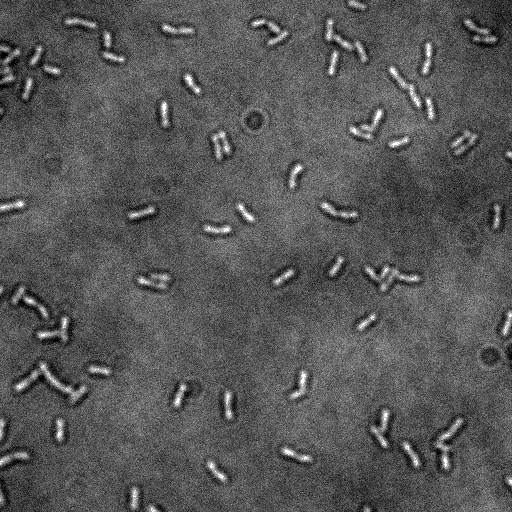

Supplement: Supplementary file 15 — Supplementary Data 1 [file 42003_2022_3634_MOESM15_ESM.zip › Figure_S4/Training_data/Brightfield/At_BF_60X_1x_2_ROI_3.tif]

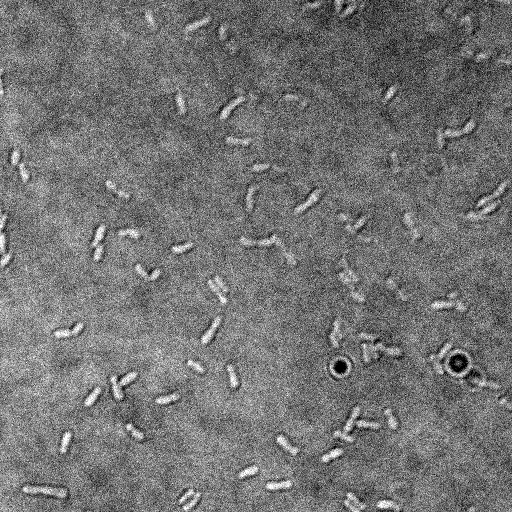

Supplement: Supplementary file 15 — Supplementary Data 1 [file 42003_2022_3634_MOESM15_ESM.zip › Figure_S4/Training_data/Brightfield/At_BF_60X_1x_3_ROI_1.tif]

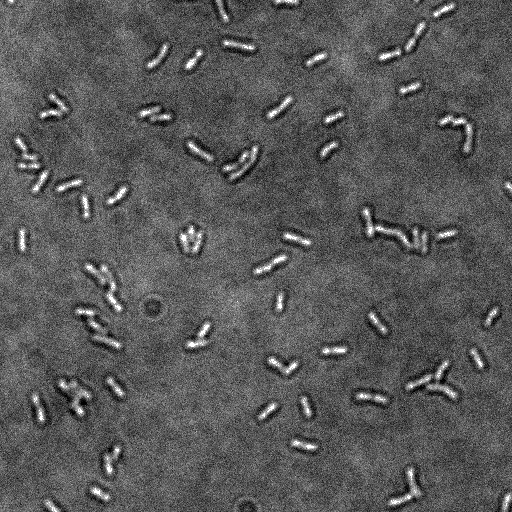

Supplement: Supplementary file 15 — Supplementary Data 1 [file 42003_2022_3634_MOESM15_ESM.zip › Figure_S4/Training_data/Brightfield/At_BF_60X_1x_3_ROI_2.tif]

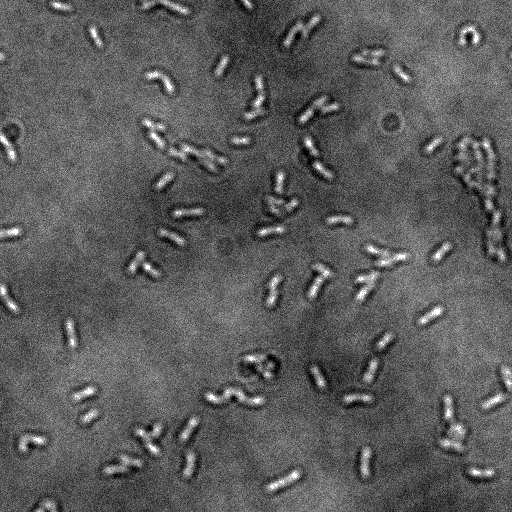

Supplement: Supplementary file 15 — Supplementary Data 1 [file 42003_2022_3634_MOESM15_ESM.zip › Figure_S4/Training_data/Brightfield/At_BF_60X_1x_3_ROI_3.tif]
