# Supplementary material for: DeepBacs for multi-task bacterial image analysis using open-source deep learning approaches
Source: Commun Biol. 2022 Jul 9;5:688. doi: 10.1038/s42003-022-03634-z (PMC9271087; doi:10.1038/s42003-022-03634-z)
Supplement: Supplementary file 16 — Reporting Summary [file 42003_2022_3634_MOESM16_ESM.pdf]

nature portfolio

Corresponding author(s): Dr. Christoph Spahn
Prof. Dr. Mike Heilemann
Prof. Dr. Ricardo Henriques

Last updated by author(s): Jun 17, 2022

Reporting Summary

Nature Portfolio wishes to improve the reproducibility of the work that we publish. This form provides structure for consistency and transparency in reporting. For further information on Nature Portfolio policies, see our Editorial Policies and the Editorial Policy Checklist.

Statistics

For all statistical analyses, confirm that the following items are present in the figure legend, table legend, main text, or Methods section.

n/a	Confirmed	
☐	☒	The exact sample size (n) for each experimental group/condition, given as a discrete number and unit of measurement
☐	☒	A statement on whether measurements were taken from distinct samples or whether the same sample was measured repeatedly
☒	☐	The statistical test(s) used AND whether they are one- or two-sided<br>*Only common tests should be described solely by name; describe more complex techniques in the Methods section.*
☒	☐	A description of all covariates tested
☒	☐	A description of any assumptions or corrections, such as tests of normality and adjustment for multiple comparisons
☐	☒	A full description of the statistical parameters including central tendency (e.g. means) or other basic estimates (e.g. regression coefficient) AND variation (e.g. standard deviation) or associated estimates of uncertainty (e.g. confidence intervals)
☒	☐	For null hypothesis testing, the test statistic (e.g. F, t, r) with confidence intervals, effect sizes, degrees of freedom and P value noted<br>*Give P values as exact values whenever suitable.*
☒	☐	For Bayesian analysis, information on the choice of priors and Markov chain Monte Carlo settings
☒	☐	For hierarchical and complex designs, identification of the appropriate level for tests and full reporting of outcomes
☒	☐	Estimates of effect sizes (e.g. Cohen's d, Pearson's r), indicating how they were calculated

Our web collection on statistics for biologists contains articles on many of the points above.

Software and code

Policy information about availability of computer code

Data collection	Data was collected using different microscope systems from various companies. DIC and fluorescence images of live S. aureus and E. coli, as well as SIM images thereof were acquired on a commercial DeltaVision OMX SR system using the AcquireSR software (v4.4.9800-1). Bright field time-lapse images of E. coli cells were acquired using a custom-built setup (Nikon Ti-E body) controlled by µManager v.1.4.14. Live-cell HNS-mScarlet and MreB-sfGFP data was recorded using a Leica SP8 confocal microscope using the LAS-X software provided by Leica Microsystems (v3.5.7.23225). PAINT super-resolution data of fixed E. coli cells was acquired using a commercial N-STORM setup controlled by NIS Elements and µManager (v.1.4.22). Confocal images of drug-treated E. coli cells were acquired on a commercial Zeiss LSM710 confocal microscope using the provided Zen software. B. subtilis FtsZ data was acquired using a custom-built setup (see https://doi.org/10.1038/s41467-021-22526-0).
Data analysis	Pre- and post processing of images for preparation of training datasets or subsequent analysis was performed using the image post-processing platform Fiji v1.53. Pre- and postprocessing steps are described in the methods section of the manuscript, the dataset description on Zenodo (see https://zenodo.org/communities/deepbacs/) and the DeepBacs wiki page (https://github.com/HenriquesLab/DeepBacs/wiki). Deep Learning networks were used within the ZeroCostDL4Mic platform mainly using notebook versions 1.12 - 1.13. All notebooks are available via the ZeroCostDL4Mic GitHub repository (https://github.com/HenriquesLab/ZeroCostDL4Mic).<br>Annotation of images for object detection was performed using the online platform makesense.ai or LabelImg v.1.8.5 (https://github.com/tzutalin/labelImg).<br>SIM reconstruction was performed using the Applied Precision's softWorx software (AcquireSRsoftWoRx v7.0.0 release RC6).<br>Trained networks were either used using the ZeroCostDL4Mic environment or corresponding Fiji plugins (StarDist, CSBDeep, Noise2Void, PureDenoise). Multi-label U-Net datasets and predictions were pre-/postprocessed using custom Fiji macros provided via the DeepBacs GitHub repository.<br>Single-molecule super-resolution data was analyzed using the Picasso software from the Jungmann group (v.0.2.8 and v.0.3.3).<br>Error estimation for SIM reconstructions and prediction of SIM images was performed using the NanoJ SQUIRREL plugin in Fiji.<br>Tracking of individual cells or MreB filaments was performed using TrackMate v7.0.7 and v7.6.1 and procedures are described in the methods

sections.

For manuscripts utilizing custom algorithms or software that are central to the research but not yet described in published literature, software must be made available to editors and reviewers. We strongly encourage code deposition in a community repository (e.g. GitHub). See the Nature Portfolio guidelines for submitting code & software for further information.

Data

Policy information about availability of data

All manuscripts must include a data availability statement. This statement should provide the following information, where applicable:

- Accession codes, unique identifiers, or web links for publicly available datasets
- A description of any restrictions on data availability
- For clinical datasets or third party data, please ensure that the statement adheres to our policy

Datasets and models generated in this work can be downloaded via Zenodo (see Supplementary Table 2), while further documentation on sample preparation, data preprocessing, training parameters and example images can be found on our GitHub repository (https://github.com/HenriquesLab/DeepBacs/wiki). Notebooks can be accessed via the ZeroCostDL4Mic repository (https://github.com/HenriquesLab/ZeroCostDL4Mic/wiki).

Field-specific reporting

Please select the one below that is the best fit for your research. If you are not sure, read the appropriate sections before making your selection.

☒ Life sciences ☐ Behavioural & social sciences ☐ Ecological, evolutionary & environmental sciences

For a reference copy of the document with all sections, see nature.com/documents/nr-reporting-summary-flat.pdf

Life sciences study design

All studies must disclose on these points even when the disclosure is negative.

Sample size	No a priori dataset size was determined. A varying number of images was recorded for the different deep learning tasks, as they have different requirements in sample sizes. Detailed information about the number of images and their parameters are found in Supplementary Table 1.
Data exclusions	As the use of deep learning requires high-quality data, datasets were manually curated. For prediction of SIM images, low-quality reconstructions (visible artifacts, wrong focal plane) were removed from the training dataset. For artificial labeling of membranes, image patches with high-quality PAINT reconstructions were used for training. The quality was assessed visually based on a continuous bacterial membrane. Images annotated for segmentation and object detection tasks were randomly drawn.
Replication	Multiple images or time series for the different DL networks were recorded in the same imaging session. It was ensured that the acquired data is representative by the different expert laboratories included in this work. For object detection (drug treated cells) and artificial labeling, data from 2-3 different experiments were included in the dataset.
Randomization	Test images were randomly separated from training images. Randomization of data during the training of the DL networks was automatically performed by the different networks.
Blinding	No blinding was required for this study.

Reporting for specific materials, systems and methods

We require information from authors about some types of materials, experimental systems and methods used in many studies. Here, indicate whether each material, system or method listed is relevant to your study. If you are not sure if a list item applies to your research, read the appropriate section before selecting a response.

Materials & experimental systems

n/a	Involved in the study	
☒	☐	Antibodies
☒	☐	Eukaryotic cell lines
☒	☐	Palaeontology and archaeology
☒	☐	Animals and other organisms
☒	☐	Human research participants
☒	☐	Clinical data
☒	☐	Dual use research of concern

Methods

n/a	Involved in the study	
☒	☐	ChIP-seq
☒	☐	Flow cytometry
☒	☐	MRI-based neuroimaging
