# Supplementary material for: DeepBacs for multi-task bacterial image analysis using open-source deep learning approaches
Source: Commun Biol. 2022 Jul 9;5:688. doi: 10.1038/s42003-022-03634-z (PMC9271087; doi:10.1038/s42003-022-03634-z)
Supplement: Supplementary file 15 — Supplementary Data 1 [file 42003_2022_3634_MOESM15_ESM.zip › Figure_S6/Figure_S6.pdf]

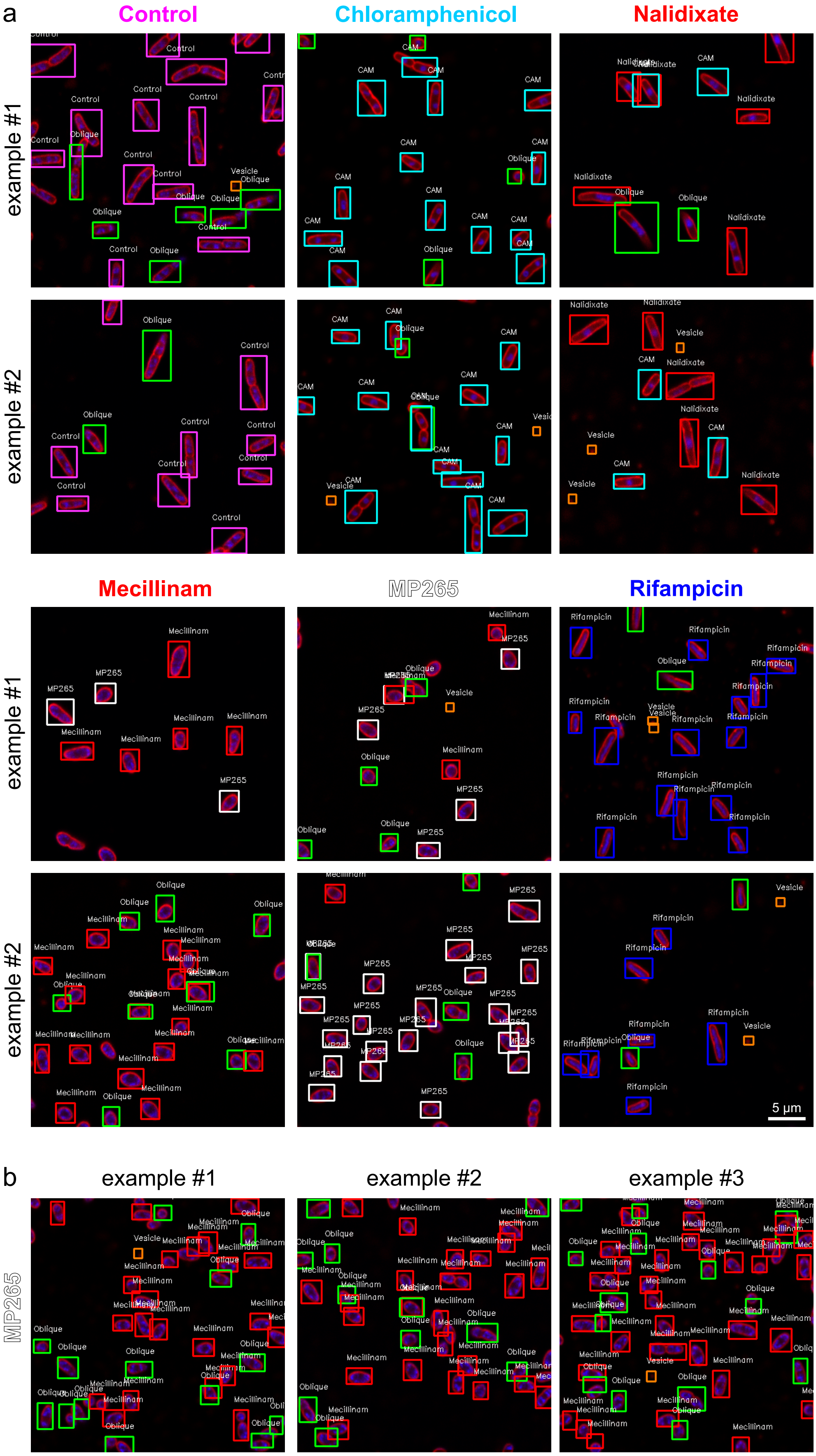

a
Control
Chloramphenicol
Nalidixate
example #1
example #2
Mecillinam
MP265
Rifampicin
example #1
example #2
5 µm
b
example #1
example #2
example #3
MP265
