# Supplementary material for: DeepBacs for multi-task bacterial image analysis using open-source deep learning approaches
Source: Commun Biol. 2022 Jul 9;5:688. doi: 10.1038/s42003-022-03634-z (PMC9271087; doi:10.1038/s42003-022-03634-z)
Supplement: Supplementary file 15 — Supplementary Data 1 [file 42003_2022_3634_MOESM15_ESM.zip › Figure_S7/Figure_S7.pdf]

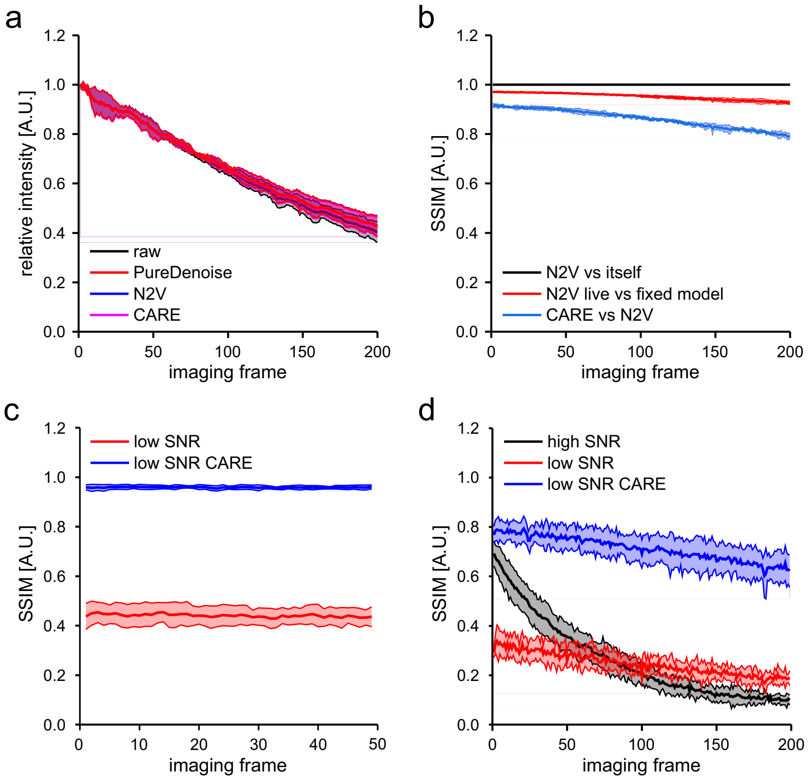

a
relative intensity [A.U.]
imaging frame
raw
PureDenoise
N2V
CARE
b
SSIM [A.U.]
imaging frame
N2V vs itself
N2V live vs fixed model
CARE vs N2V
c
SSIM [A.U.]
imaging frame
low SNR
low SNR CARE
d
SSIM [A.U.]
imaging frame
high SNR
low SNR
low SNR CARE
