# Supplementary material for: DeepBacs for multi-task bacterial image analysis using open-source deep learning approaches
Source: Commun Biol. 2022 Jul 9;5:688. doi: 10.1038/s42003-022-03634-z (PMC9271087; doi:10.1038/s42003-022-03634-z)
Supplement: Supplementary file 15 — Supplementary Data 1 [file 42003_2022_3634_MOESM15_ESM.zip › Figure_S8/Figure_S8.pdf]

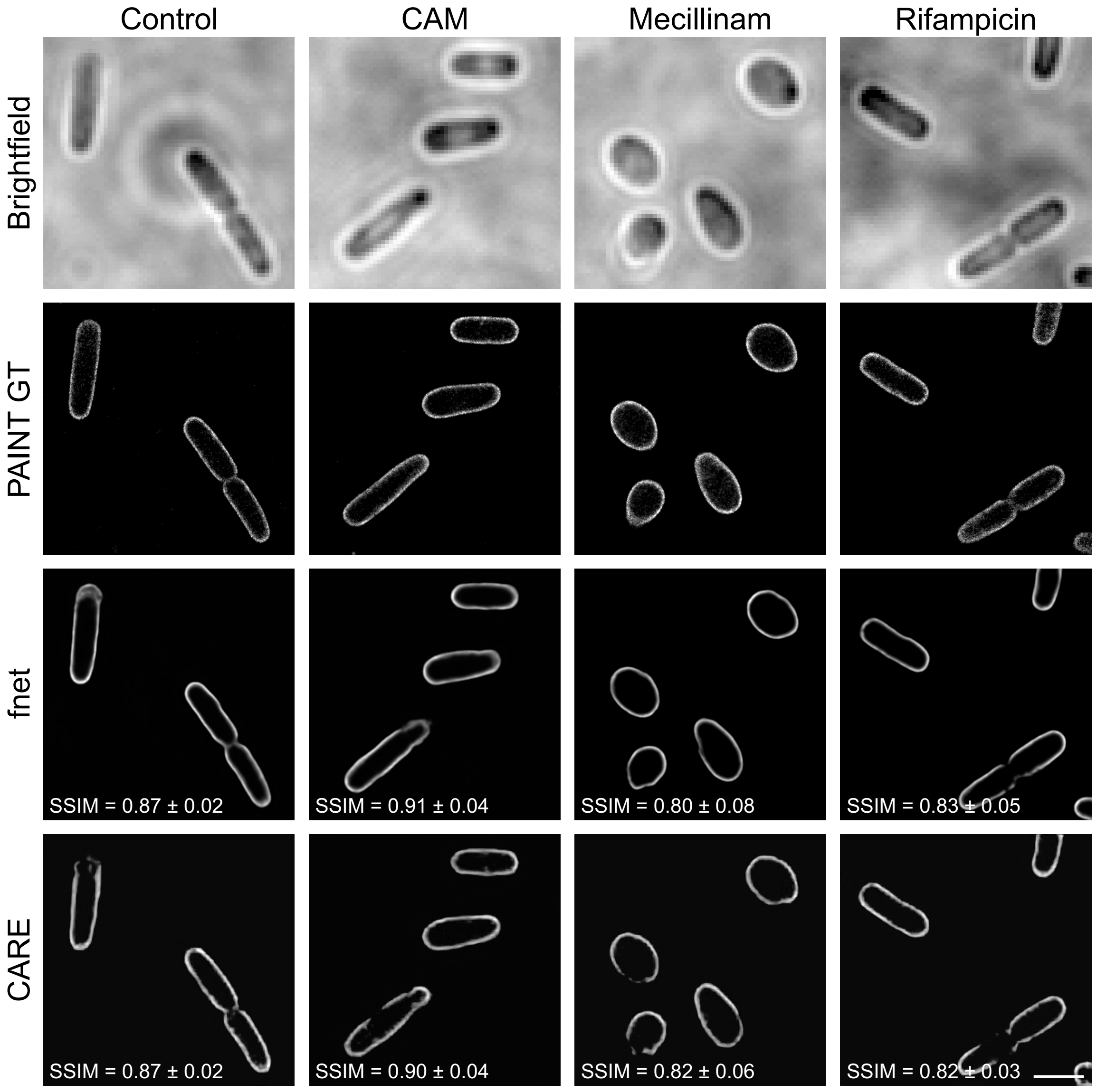
Control
CAM
Mecillinam
Rifampicin
Brightfield
PAINT GT
fnet
CARE
SSIM = 0.87 ± 0.02
SSIM = 0.91 ± 0.04
SSIM = 0.80 ± 0.08
SSIM = 0.83 ± 0.05
SSIM = 0.87 ± 0.02
SSIM = 0.90 ± 0.04
SSIM = 0.82 ± 0.06
SSIM = 0.82 ± 0.03
