# Supplementary material for: DeepBacs for multi-task bacterial image analysis using open-source deep learning approaches
Source: Commun Biol. 2022 Jul 9;5:688. doi: 10.1038/s42003-022-03634-z (PMC9271087; doi:10.1038/s42003-022-03634-z)
Supplement: Supplementary file 15 — Supplementary Data 1 [file 42003_2022_3634_MOESM15_ESM.zip › Figure_S9/Figure_S9.pdf]

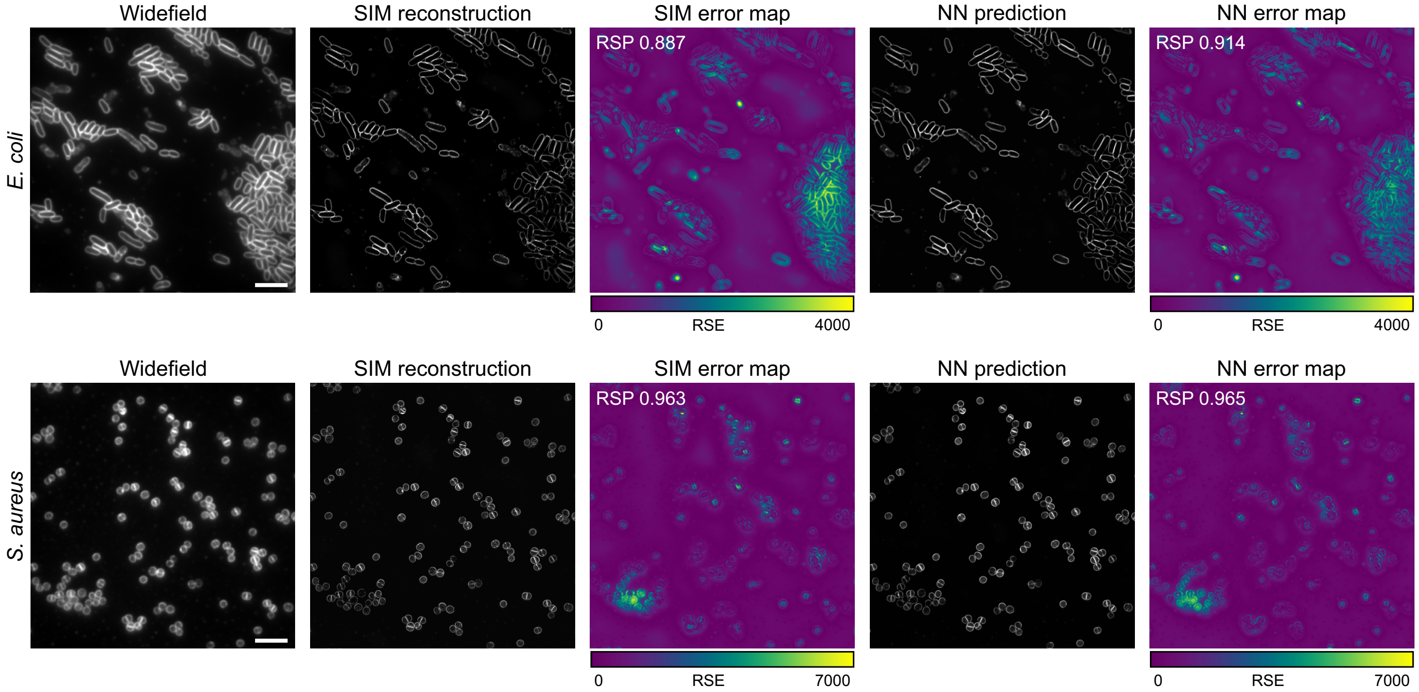
Widefield
SIM reconstruction
SIM error map
NN prediction
NN error map
E. coli
RSP 0.887
RSP 0.914
0
RSE
4000
0
RSE
4000
Widefield
SIM reconstruction
SIM error map
NN prediction
NN error map
S. aureus
RSP 0.963
RSP 0.965
0
RSE
7000
0
RSE
7000
